# CAdir: Joint clustering of cells and genes for single-cell transcriptomics with visualization-driven cluster quality assessment

**DOI:** 10.1371/journal.pcbi.1014418

**Published:** 2026-06-30

**Authors:** Clemens Kohl, Martin Vingron

**Affiliations:** Department of Computational Molecular Biology, Max Planck Institute for Molecular Genetics, Berlin, Germany; Wuhan University, CHINA

## Abstract

Clustering for single-cell RNA-seq aims at finding similar cells and grouping them into biologically meaningful clusters. Many available clustering algorithms however do not not provide the cluster defining marker genes or are unable to infer the number of clusters in an unsupervised manner as well as lack tools to easily determine the quality of the label assignments. Therefore, clustering quality is commonly evaluated by visually inspecting low-dimensional embeddings as produced by, e.g., UMAP or t-SNE. These embeddings can, however, distort the true cluster structure and are known to produce radically different embeddings depending on the chosen hyperparameters. In order to improve the interpretability of clustering results, we developed CAdir, a clustering algorithm that can infer the number of clusters in the data, determine cluster specific genes and provides easy to interpret diagnostic plots. CAdir exploits the geometry induced by correspondence analysis (CA) to cluster cells as well as cluster associated genes based on their direction in CA space. Using the angle between the cluster directions, it is able to automatically infer the number of clusters in the data by merging and splitting clusters. A comprehensive set of diagnostic and explanatory plots provides users with valuable feedback about the clustering decisions and the quality of the final as well as intermediary clusters. CAdir is scalable to even the largest data set and provides similar clustering performance to other state-of-the-art cell clustering algorithms in our benchmarking. CAdir can be downloaded from GitHub: https://github.com/VingronLab/CAdir.

## Author summary

Clustering single-cell RNA-seq data can be highly subjective due to the lack of means to evaluate the quality of the clustering and the lack of information on why the clustering algorithm decided to group certain cells together. Ultimately, a clustering’s quality is determined by how well it is able to distinguish biologically meaningful sub-groups. In practice, this is often assessed by the expression of marker genes, which have to be determined afterwards through statistical methods. CAdir offers an all-in-one solution that co-clusters cells and genes simultaneously and provides visualizations that not only allow for the determination of the cluster quality but also help identify genes of interest for each cluster. These so-called Association Plots of the clustering results help identify malformed clusters and outliers in the data and also highlight the most highly associated marker genes for each cluster. Because for many single-cell RNA-seq experiments the number of cell types is not known in advance, CAdir can determine the number of clusters without user input and annotates clusters with the best matching cell type based on their co-clustered marker genes. In combination with its fast runtime, CAdir can help to speed up routine tasks and make them more efficient.

## Introduction

Single-cell RNA sequencing (scRNA-seq) has allowed researchers to study the heterogeneity present in their data and to better understand how different cell types behave. Yet, without prior knowledge and experimental validation, it is often difficult to determine which cell types are present in the data. This is further compounded by the fact that there is no clear definition of a cell type and researchers are often interested in different levels of granularity. Clustering algorithms aim to solve this by unbiasedly grouping cells into biologically meaningful clusters based on their gene expression profiles, thereby helping to discover previously unknown cell types.

Many clustering approaches were developed, including k-means, hierarchical clustering, community detection, and density estimation based approaches [1]. Despite the large number of clustering algorithms available, only a fraction is capable of determining the cluster defining marker genes or estimating the number of cell types in the data [1,2]. Given the importance of the task, a number of different approaches has been developed to determine the number of clusters, the majority of which use approaches based on intra-/inter-cluster similarity, modularity, stability metrics or eigenvector metrics [2]. Popular approaches for scRNA-seq such as Seurat [3] or Monocle3 [4] use k-Nearest-Neighbor graphs to unfold the high dimensional manifold on which the data lies and then employ graph clustering approaches, e.g., Leiden [5], to find communities in the data without the need to specify the number of clusters *a priori*. While this modularity based approach is able to robustly deal with large data sets, its decision-making process is often opaque to the user and lacks intuitive ways to evaluate the assigned cluster labels.

Spectral clustering [6] is a another popular clustering approach that has also been successfully applied to transcriptomics data. SIMLR [7] and SinNLRR [8] built upon Spectral clustering to perform clustering on scRNA-seq. Because Spectral clustering is graph-based, similarly to the modularity-based approaches, careful construction and pruning of the underlying similarity graph is required for optimal performance. In recent years, deep learning methods such as scDeepCluster [9] and scG-cluster [10] have been proposed that use flexible neural networks to identify cell clusters. Due to the number of learnable parameters deep learning methods have the potential to identify clusters that would otherwise be difficult to differentiate from each other. However, training and running deep learning methods requires large amounts of computational resources as well as specialized hardware and can suffer from overfitting to the data and poor generalizability in the case of pre-trained models. Similarly, newly emerging single-cell foundation models (scFMs) such as Geneformer [11] that have been trained on millions of cells encompassing many different cell types from different tissues enable new forms of cell type discovery. The cell embeddings obtained from such scFMs however ultimately still require the application of more traditional clustering algorithms to identify similar cells [12]. Other clustering approaches for example include hierarchical clustering or density-based methods such as DBSCAN [1].

Cell types are typically defined through patterns of gene expression and the analysis of scRNA-seq data often aims at uncovering biological processes within clusters of cells. Classical clustering however does not provide an answer to which biological process defines a cluster of cells. In contrast, biclustering aims at grouping cells and their defining genes concurrently in order to identify groups of cells as well as the patterns of gene expression that characterize them. This allows for a more comprehensive description of cell communities and avoids statistical pitfalls associated with re-using the same data to test for cluster marker genes after performing cell clustering, a phenomenon called double-dipping [13].

To this end, a number of biclustering algorithms have been developed for microarray and scRNA-seq data. The first biclustering algorithm developed specifically for scRNA-seq data, BackSPIN [14], uses the SPIN [15] algorithm to sort both the cells and genes based on the cell-cell and gene-gene correlations. BackSPIN then recursively splits the correlation matrix into sub-matrices until a maximum number of split cycles or the stopping criteria is reached. QUBIC2 [16] builds on their previously published biclustering algorithm QUBIC [17] and aims to identify functional gene modules (FGMs), in large scale gene expression data. FGMs are groups of genes that together serve a biological function, e.g., a pathway. QUBIC2 finds FGMs by first discretizing the expression matrix using a Gaussian-Mixture-Model and sorting gene-pairs based on how many cells they are co-expressed in. The most promising gene-pairs are then used as a core from which the algorithm adds new genes based on a Kullback-Leibler divergence score [16,18]. Although FGMs can be of great interest and can help elucidate common genetic programs in cell types, they often are not unique to a cell type but instead several cell types might share FGMs. Therefore, FGMs are not well suited in uniquely identifying a cell cluster.

Other biclustering algorithms, such as BiSNN-Walk [19], scDBic [20] and CAbiNet [21] use a Shared-Nearest-Neighbors graph (SNN-graph) to identify biclusters. BiSNN-Walk iterates between an inner and outer loop, where in the outer loop candidate cell clusters are identified using the Walktrap algorithm [22] on the SNN-graph. Using the most highly expressed genes for each cell cluster to reduce the expression matrix, the sub-matrices are then refined in the inner loop until convergence [18,19]. The recently published deep learning method scDBic [20] first learns cell embeddings using an autoencoder architecture, which are then used to construct the SNN-graph. Analogous to BiSNN-Walk, the Walktrap algorithm is used to cluster cells in the SNN-graph. Importantly, both BiSNN-Walk and scDBic select genes based on their expression in a cell cluster, making them fundamentally similar to other methods that determine marker genes based on a cell clustering, such as differential gene expression analysis.

CAbiNet [21] on the other hand, simultaneously clusters cells and genes using a SNN-graph that connects both cells and genes based on their association in correspondence analysis (CA) space. By performing graph clustering using, e.g., Leiden [5] or Spectral Clustering [6] on this cell-gene graph, biclusters of cells and genes can then be obtained. Additionally, CAbiNet can visualize both cells and genes in a single two-dimensional embedding. Using UMAP to embed the cell-gene graph in a so-called biMAP, this approach allows for quick annotation of the cell clusters using the marker genes that are located close to the cells expression the genes in question. However, constructing the dense SNN-graph for both cells and genes requires substantial amounts of memory, making it unsuitable for biclustering on large data sets.

In order to evaluate the quality of a clustering, researchers often rely on two-dimensional embeddings of their data, such as t-distributed stochastic neighbor embedding (t-SNE) [23] or Uniform Manifold Approximation and Projection (UMAP) [24] plots. However, these heuristic methods depend on a large number of hyperparameters and as reported by Chari et al. [25], the prediction of a cell’s label based on its k-Nearest-Neighbors is consistently worse using UMAP coordinates than it is with PCA coordinates. Furthermore, UMAP does not take the local density of points into account, resulting in misleading visualizations [26]. For the interpretability of clustering results, the fact that distances in a UMAP or t-SNE cannot be interpreted is particularly problematic, as it often leads to erroneous conclusions drawn from visualizations of, e.g., two neighboring clusters in a UMAP [25]. This issue also extends to other methods such as, e.g., CAbiNet, whose biMAP is fundamentally still based on UMAP and consequently shares many of its shortcomings, most notably the inability to assess cluster quality based on the embedding.

It is generally recommended not to use UMAP coordinates for clustering due to their stochastic nature and dependency of hyperparameter choices. It therefore stands to reason that they should equally not be used to determine a clustering’s quality, particularly as it can be difficult to understand *why* an embedding created by UMAP, t-SNE or other non-linear methods looks a specific way. If the goal however is to draw a conclusion from the visualization, an interpretable embedding is paramount. Despite their reported shortcomings, UMAP and related methods such as t-SNE have become staple tools used for determining the quality of a clustering due to a lack of easy to use alternatives.

**Fig 1:**
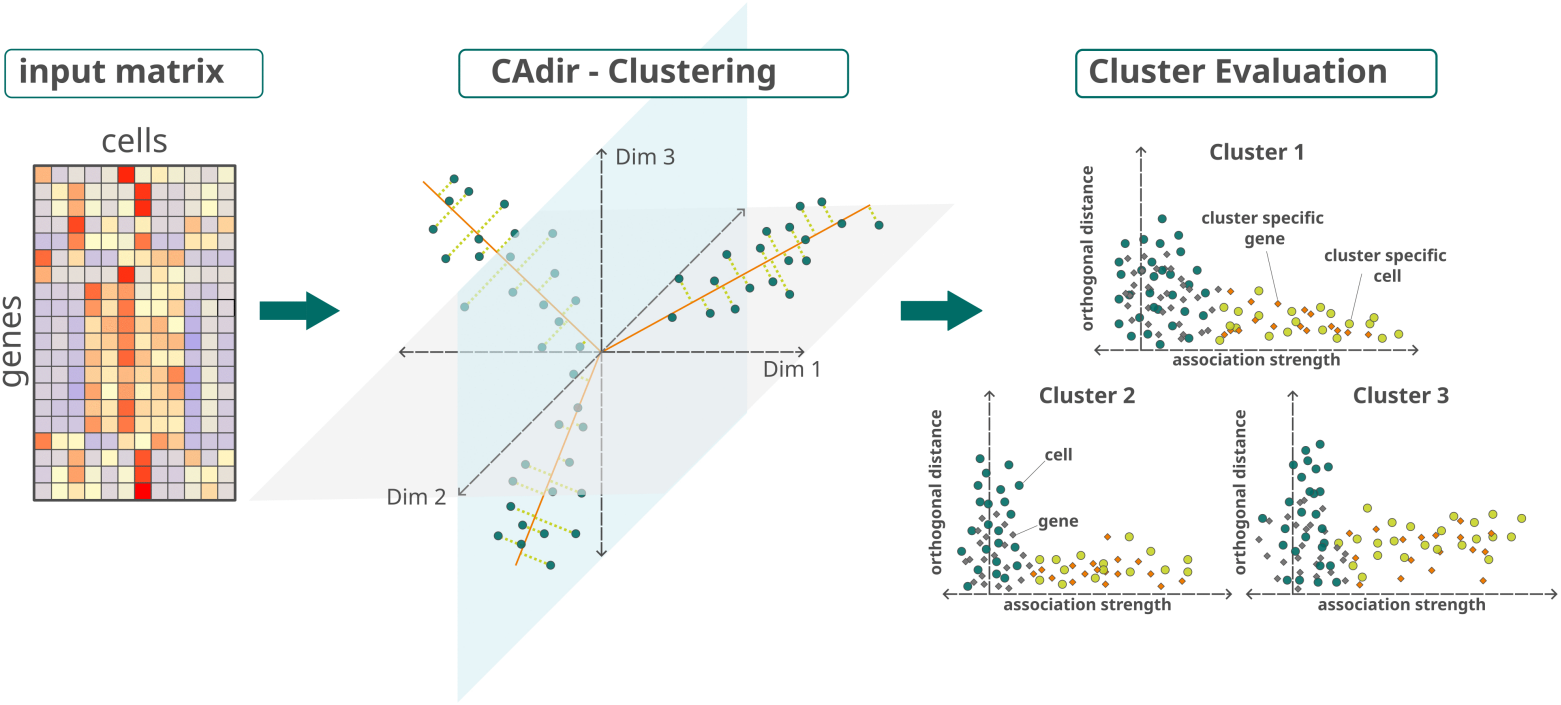
CAdir workflow. Starting from a count matrix, CAdir first performs correspondence analysis (CA) to embed cells and genes together in a dimensionality-reduced space, where cells and their marker genes are arranged along shared directions. CAdir then jointly clusters cells and genes by determining the cluster directions for cells and assigning each gene to a cluster, with the number of clusters automatically inferred by splitting and merging directions based on the angles between them. Each cluster is visualized in an Association Plot, which shows how closely cells and genes are associated with the cluster’s direction in CA space and thereby allows for quality assessment of the results. The co-clustered genes are used to annotate each cluster.

Here we present Correspondence Analysis directional clustering (CAdir), a clustering algorithm that co-clusters cells and genes by their direction in correspondence analysis (CA) [27] space. CA arranges points within a simplex and places points with similar properties along the same direction. CAdir exploits this property of CA not only to cluster both the cells and genes, but also to estimate the number of groups present in the data. Using the angle between cluster directions as a measure of similarity, CAdir is able to infer when a cluster should be split or merged with another cluster. This dynamic creation and removal of clusters allows CAdir to determine the number of clusters without any prior knowledge about the data (Fig 1).

Unlike other clustering approaches, CAdir offers an intuitive and easy to interpret approach that enables users to evaluate the clustering performance through diagnostic plots and aids in understanding the intermediate steps that lead to the final result (Fig 1). The co-clustered genes further provide the basis for annotating clusters and identifying cluster defining marker genes. In contrast to other cell clustering and biclustering algorithms, CAdir offers a number of advantages: CAdir is faster than all tested cell clustering algorithms, while additionally providing the co-clustered genes. Despite its fast runtime, CAdir outperforms all biclustering algorithms in our benchmarking, including recent algorithms such as, e.g., CAbiNet. In contrast to other biclustering algorithms, CAdir can additionally visualize the found biclusters succinctly in an interpretable way that allows the identification of the most important co-clustered genes for each bicluster and provides a fast to compute ranking for each co-clustered gene. Lastly, whereas many algorithms require the number of clusters as an input, CAdir can independently estimate the number of clusters in the data.

In this paper we introduce the novel clustering approach of CAdir and show that it’s performance is as good as other state-of-the-art cell clustering algorithms, while providing easy to interpret diagnostic outputs, a clustering of both cells and genes as well as determining the number of clusters. Using CAdir’s powerful plotting functionality, we further show how the output of CAdir can be used to better understand and interpret the clustering results.

## Results

### Clustering cells by direction

CA is used to embed cells and genes into the same dimensionality reduced space. This not only helps to remove noise from the data, but CA also arranges similar points along specific directions. Therefore, cells with comparable expression profiles will fall along the same direction, and the genes characteristic to this cluster of cells will also lie along the same direction. Generally, the further out a cell or gene is positioned along a direction, the more strongly it exhibits cluster specific qualities, e.g., the expression of cluster specific marker genes. CAdir makes use of these unique geometric properties of CA to cluster the data.

**Fig 2:**
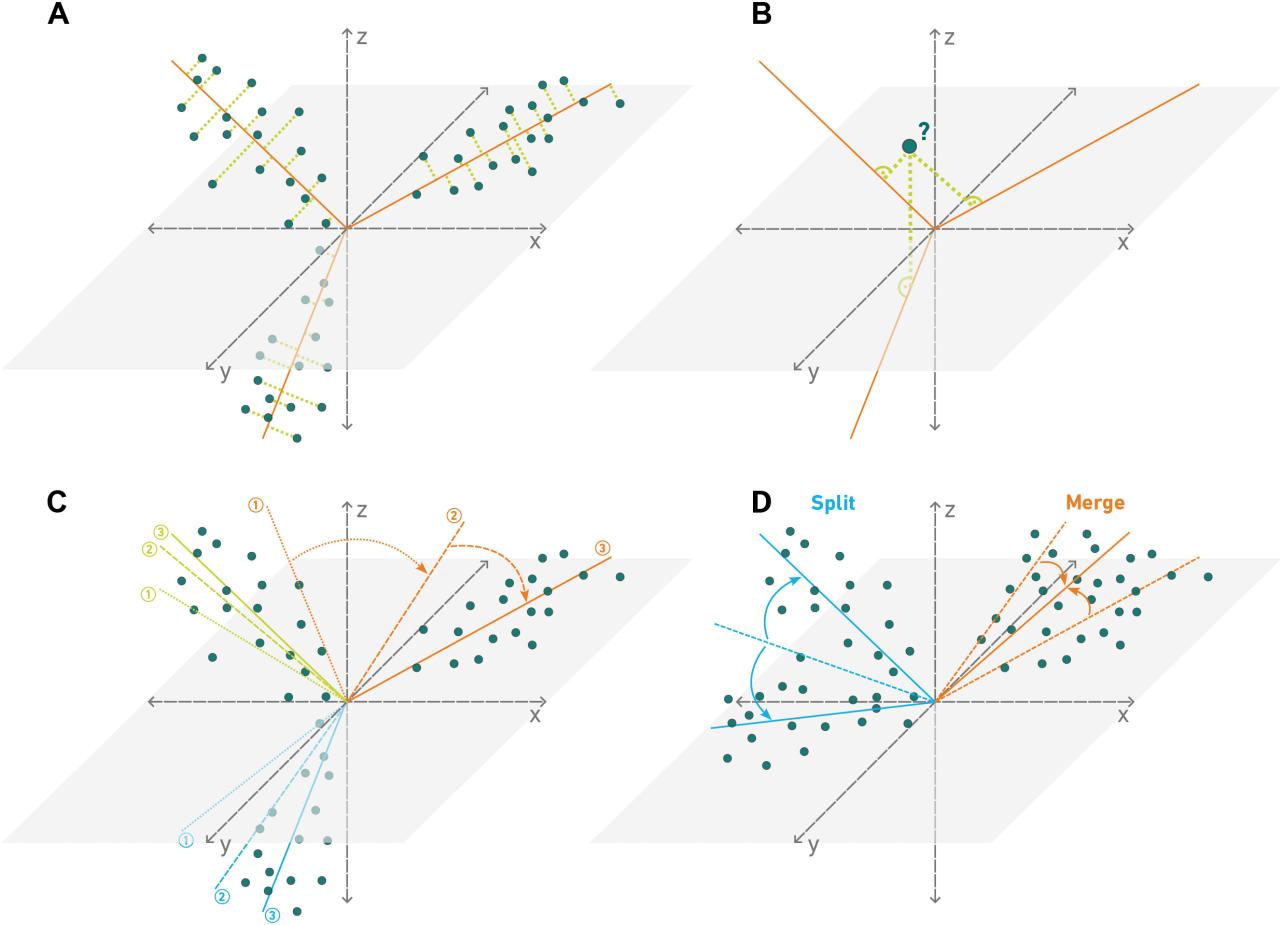
Clustering by direction - Overview. **A**, CAdir clusters cells that lie along a direction in CA space by assigning them to the best fitting line. **B**, Each cell is assigned to the closest cluster direction based on the shortest orthogonal distance of the cell to a direction. The cluster directions are then recomputed based on the changed cluster assignments. **C**, CAdir updates the clusters iteratively: Starting from an initialization (1), cells are assigned to the closest direction and the cluster direction is recomputed (2). This is repeated until the final cluster directions are found (3). **D**, CAdir is further able to independently detect low-quality clusters and thereby infer the number of clusters in the data. Depending on the angle between cluster directions, it either splits a cluster into two new clusters or merges two or more directions into a single cluster.

Since in CA space clusters of cells and their cluster-specific genes get placed along a direction from the origin, our CAdir algorithm looks for distinct, densely populated directions in CA space. Fig 2 shows the basic steps of CAdir’s clustering approach as explained in the Methods section. After initializing a pre-defined number of directions, CAdir assigns each cell to the closest direction (see Fig 2A and 2B) and re-computes the direction for each cluster through total least squares regression. This is repeated for a set number of iterations until a stable first clustering is reached (Fig 2C). Then, cluster directions with small angles to each other are merged, whereas sub-clusters with large angles within a cluster are split (Fig 2D). CAdir determines by itself the number of clusters in the data based on a cutoff angle inferred from the data. The output of CAdir is a set of Association Plots, each visualizing a cluster of cells with its specific genes. This allows for intuitive interpretation of the individual cluster qualities. Based on the cluster specific genes CAdir provides a mapping of clusters to cell types from a given gene set library. A more detailed description of the clustering algorithm, the automated cell type annotation as well as Association Plots can be found in the Materials and Methods and in the Supporting Information S1 Appendix.

#### Tabula Muris Limb Muscle data.

**Fig 3:**
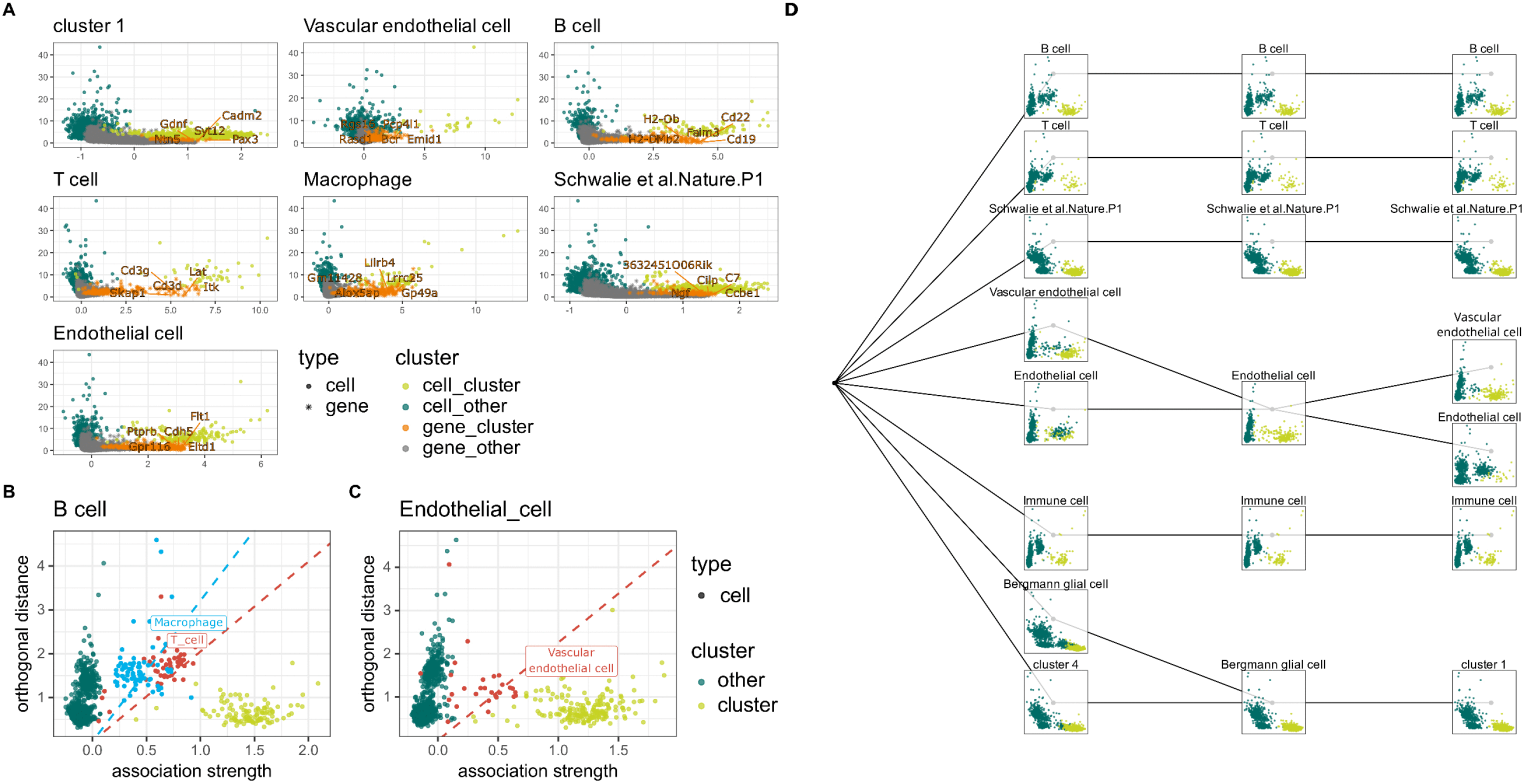
Clustering of Tabula Muris Limb Muscle data. **A**, Association Plots of all clusters determined by CAdir with cluster annotations obtained by gene set over-representation analysis. Clustered cells are colored in lime green and co-clustered genes in orange. Other cells and genes are colored in dark green and grey respectively. For each cluster, the top 5 most highly ranked genes by Sθ-score are labelled. Association plots for **B**, the B cell cluster with the directions of Macrophages (blue) and T cells (red) and the associated cells highlighted and **C**, Association Plot for the Endothelial cell cluster with the direction and the cells of the Vascular endothelial cell cluster highlighted in red. **D**, Graph of the splits and merges performed by CAdir. Each row in the graph corresponds to a single iteration of the algorithm (iterations without splits and merges are removed) and each node to a cluster. At each node an association plot of the cells (principal coordinates) is shown with clustered cells in lime green and all other cells in dark green. The clusters are annotated by the automatic cell type annotation using the co-clustered genes.

We clustered the Tabula Muris Limb Muscle (LM) data [28] with 1882 cells using CAdir and used the co-clustered genes to annotate the cells using the automated cell type annotation. As can be seen in Fig 3A, CAdir finds 7 directions that summarize the data and correspond well to the provided ground truth clustering (S1C Fig).

CAdir provides visualization tools to comprehensive judge the quality of the obtained clusters: A well-separated cluster of cells forms an elongated cloud along a direction in CA space. Cluster specific genes, as well as cells more representative of the cluster, are found further out in the same direction. Association Plots are ideally suited to visualize the clusters obtained through CAdir, as they allow us to visualize how closely points are associated with a direction in CA space. In an Association Plot, the points’ coordinates are determined by their projection onto a given direction, as well as their distance to that direction, which perfectly aligns with the clustering mechanics of CAdir. The Association Plots in Fig 3A are drawn for each cluster, with clustered cells in lime green, co-clustered genes in orange and the remaining cells and genes in dark green and gray respectively. Generally, in a well separated cluster the direction-determining cells have a large x-coordinate in the Association Plot, which corresponds to their projection onto the line. Simultaneously, a low y-coordinate (distance to the line) is desirable, as it indicates that the cluster is not very dispersed and is not, or only mildly, correlated with other directions. For example, the T-cell cluster in Fig 3A is apparently well-defined and, interestingly, so is the unannotated Cluster 1. Although well-defined as a cluster, the automatic annotation is undecided here but can be resolved with respect to the annotation of the experiment (S1C Fig).

The angles between cluster directions can also be seen in an Association Plot and this feature can be used to better understand the relationship between clusters. For example, a zoom into the B cell Association Plot displaying cells and directions in principal coordinates shows a low angle between it and the Macrophages and T cell cluster (Fig 3B), as would be expected based on their largely similar expression profiles. Similarly, the projection of the Vascular endothelial cell cluster direction into the Association Plot has a lower angle than other cell types to the Endothelial cells (Fig 3C). The angle between clusters can therefore be used as an indicator of similarity between clusters. Importantly, as S1D and S1E Fig (cells in standard coordinates) shows, the angle between the directions is only conserved when plotting the cells in principal coordinates. The heat map in S1A Fig shows the angle between cluster directions and S1B Fig displays this graphically in terms of the directions of a cluster in another cluster’s Association Plot. There one sees, e.g., that there is some relationship between endothelial and vascular endothelial cells, but no similarity between those and the immune cells.

For many applications it can be important to know why a cluster was merged or split. The graph in Fig 3D visualizes the clustering decisions of the algorithm: Starting from the unclustered data as the tree root, each level represents a split or merge performed by CAdir. The intermediate clustering steps as well as the final clustering were annotated using the method described in Section “Gene assignment.” To further improve the interpretability, an Association Plot depicting the state of a cluster at a given iteration gives valuable feedback about why it was split, merged, or left as is.

Throughout the iterative process, CAdir combined two clusters into cluster 1 and additionally merged and subsequently re-split the two endothelial cell clusters (Vascular endothelial cell and Endothelial cell respectively). This seemingly redundant step can help to find a better direction for both clusters if the initial clustering included cells from both cell type. By first merging the clusters, CAdir can break out of a local minimum and attempt to find directions that better explain the data.

#### Identifying low-quality clusters.

**Fig 4:**
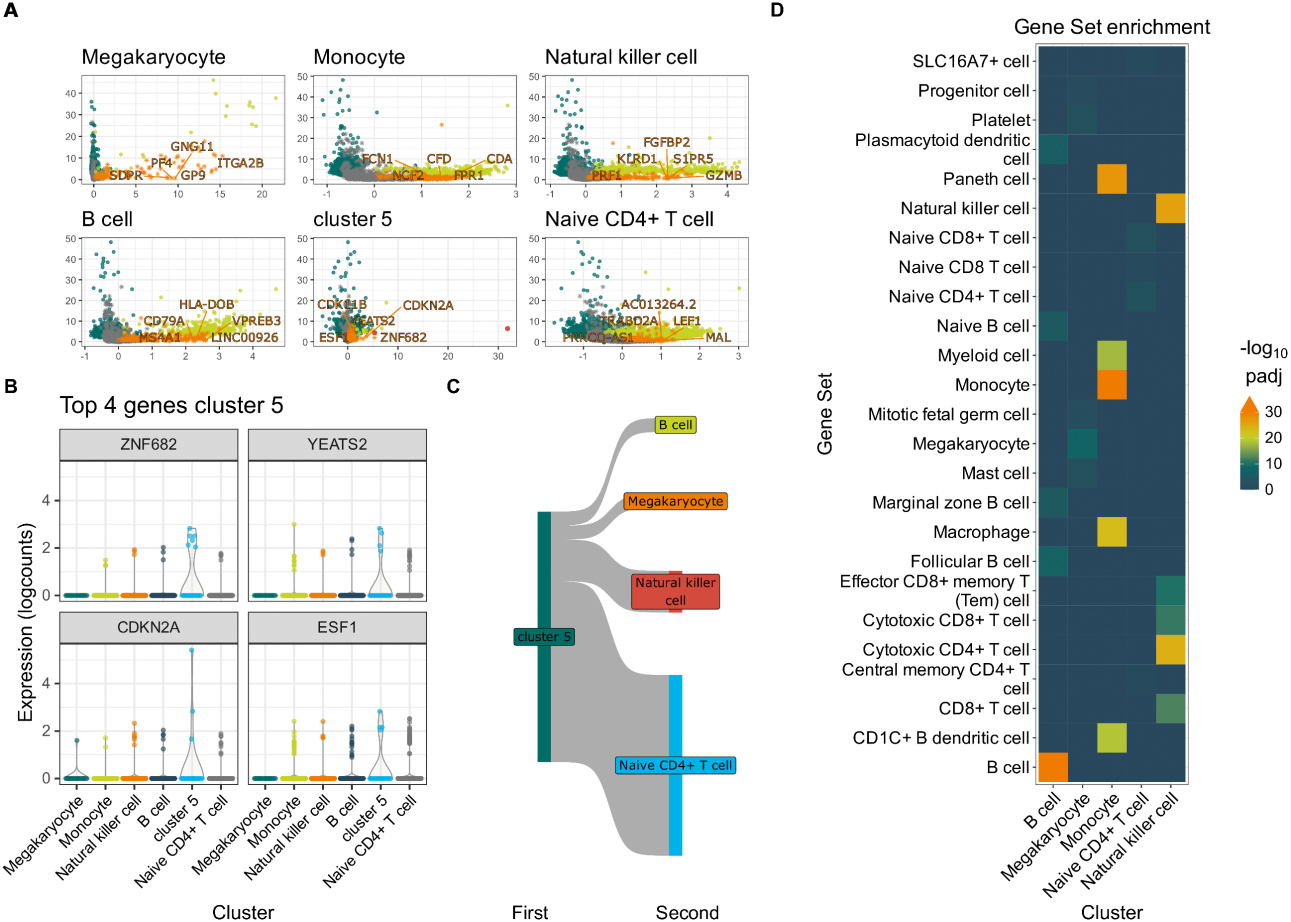
Clustering results for PBMC3k data. **A**, Association Plots for the 6 clusters obtained with CAdir. Clustered cells are colored in lime green, co-clustered genes in orange. Other cells are colored in dark green and the remaining genes in grey. The 5 genes with the highest Sθ-score for each cluster are labelled. The two cells identified as outliers in cluster 5 are marked in red in the respective association plot. **B**, Violin plots of the log-expression of the four genes with the highest Sθ-score for cluster 5. **C**, Sankey plot showing the re-assignment of the remaining cells from cluster 5 after re-clustering to other clusters. **D**, Results of the gene set over-representation analysis used to annotate clusters. The heatmap shows the negative log_10_ adjusted p-values. In order to provide better visual interpretability of the results, the color scale is capped at 30 and gene sets with an adjusted p-value lower than 10^−30^ are therefore all colored in orange.

To demonstrate the use of CAdir for quality control and how to assess cluster quality, we clustered the PBMC3k data set [29] with CAdir parameters chosen on purpose to result in a low-quality clusters. The clustering results are shown in Fig 4A. While most clusters show a large number of cluster specific cells and genes, cluster 5 is dominated by only a small number of cells and genes towards the right of the plot (Fig 4A). The majority of other cells and genes in cluster 5 do not seem to be strongly associated with the cluster and can be found close to the origin. Cluster 5 consists of only a small number of cells (20 cells), and could not be annotated by our automatic cell type annotation.

Ranking the genes using Sθ-scores [30], we identified the 4 genes that are most highly associated with cluster 5: *ZNF682*, *YEATS2*, *CDKN2A* and *ESF1* (see Fig 4B). As can be seen in Fig 4B, most of these genes are expressed only moderately even in cluster 5. *ZNF682*, which is considerably higher expressed in cluster 5, is predicted to be involved in transcription regulation (https://www.ncbi.nlm.nih.gov/gene/91120, accessed on 2025.02.18), but not known to be indicative of a cell type. The gene *CDKN2A*, however, codes for two proteins, p16^INK4a^ and p14^ARF^, both of which cause cell cycle arrest and generally lead to apoptosis or senescence [31,32]. *CDKN2A* is particularly highly expressed in some cells of cluster 5, making it likely that the cluster is mostly based on apoptotic and outlier cells.

Ranking the cells in cluster 5 similarly to the genes, we removed all cells and genes with Sθ-scores above 0. In total this includes 2 cells and 13 genes. The removed cells are also marked in red in the Association Plot for cluster 5 in Fig 4A. After re-clustering the data with the outliers removed, we obtained the same 5 clusters as shown in Fig 4A, excluding cluster 5 (see also S2A Fig). The remaining cells that were originally clustered in cluster 5 are merged into the Naive CD4^+^ T cell, Natural killer cell, Megakaryocyte and B cell clusters (Fig 4C), further confirming that cluster 5 was dominated by outliers. The final clustering generally corresponds well with the clusters from the annotation (Sankey plot S2B Fig), but letting CAdir pick the cutoff angle leads to a slightly more conservative clustering. As can be seen in S2B Fig, the automatic cell type annotation is able to correctly identify the majority cell type of the clusters based on the co-clustered genes.

One exception is the Megakaryocyte cluster, which is labelled as Platelet cells in the ground truth annotation. This can potentially be attributed to the fact that Megakaryocytes produce Platelet cells, which, as they do not have a nucleus, inherit the mRNA from their parent cell. In cases such as this where two cell types share a large number of expressed genes, the automatic cell type annotation can sometimes mislabel clusters. The results from the gene set over-representation analysis presented in Fig 4D show that the second most significant gene set (tied with Mast cells) for the Megakaryocyte cluster is in fact Platelet cells. Similarly, Fig 4D shows that some clusters, such as B cells, have a very clear annotation with one gene set having a much lower adjusted p-value, and other clusters such as Naive CD4^+^ T cell could have two or more gene sets with very similar p-values. This is also reflected in the Association Plot for the cluster: The plot for the Naive CD4^+^ T cell cluster shows that the clustered cells are less separated from the other cells compared to, e.g., the B cell or Monocyte cluster. Inspecting the cell type annotation results can therefore give additional insights about how well defined a cluster is. An in depth-discussion and validation of the marker genes recovered by CAdir can be found in the Supporting Information S1 Appendix Section “Validation of Marker Genes,” as well as S3 Fig and Table B in S1 Appendix.

We re-clustered the PBMC3k data set with appropriate parameters for the PBMC3k data (see Section “Discussed Data” in Methods) and compared the co-clustered genes with those obtained by using scran’s scoreMarkers [33] (for more details see Supplementary Results Section “Comparison to Scran Marker Gene Detection” in S1 Appendix). As shown in S4 Fig, CAdir’s co-clustered genes are typically uniquely expressed in the cluster, whereas scran often also ranks genes high that expressed in several cell types, albeit at a lower level. CAdir is therefore better in identifying marker genes that delineate the clusters from each other. Scran on the other hand can be used when the main interest lies in finding differentially expressed genes. Overall, for most clusters the overlap between the genes identified by CAdir in scran is between 30–60 % (see S5 Fig).

### Benchmarking

In order to test the performance of CAdir we benchmarked it against 10 other clustering algorithms. Namely, we tested it against k-means [34], CAbiNet [21], RaceID [35,36], Seurat [3], Monocle3 [4], SC3 [37], SIMLR [7], DivBiclust [38] and the two deep learning based methods scDeepCluster [9] and scG-cluster [10]. Because both CAdir and RaceID can either be supplied parameters that determine the number of clusters or be run in an automatic mode in which they infer the number of clusters by themselves, we split it into CAdir/RaceID and CAdir_auto/RaceID_auto to better differentiate between the two modes.

The tested clustering algorithms differ substantially in their function: CAdir, CAbiNet, RaceID, Seurat, Monocle3 and DivBiclust are able to determine the number of clusters by themselves, whereas k-means, SC3, SIMLR, scDeepCluster and scG-cluster output a pre-specified number of clusters. In most real world scenarios, the number of clusters *k* is not known *a priori*, and algorithms that automatically determine the number of clusters are therefore often preferred. Additionally, during benchmarking it is not possible to test all possible values of *k*, which can impact the performance of the affected algorithms in the benchmarking.

**Fig 5:**
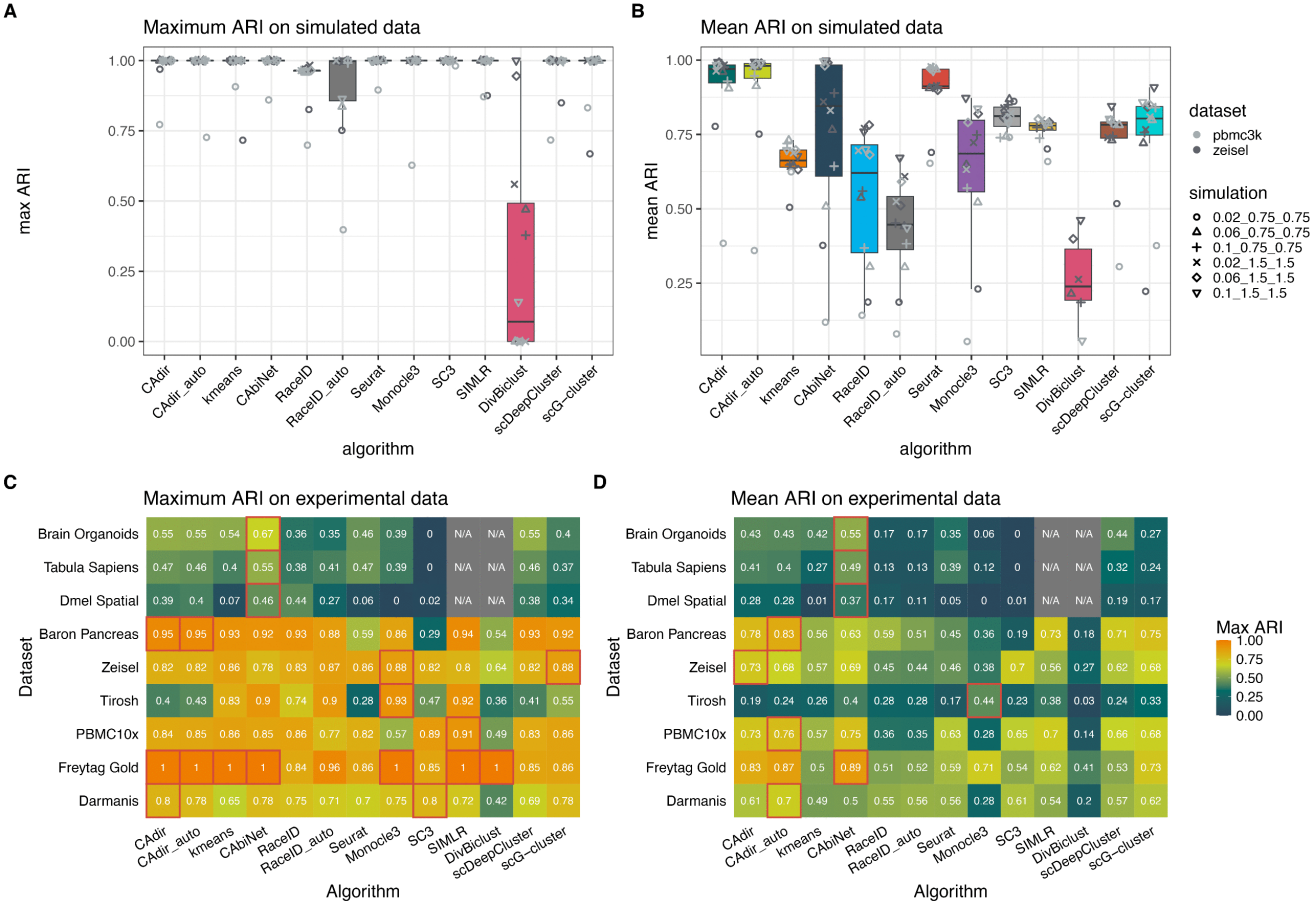
Cell clustering benchmarking. **A**, Highest achieved (maximum) and **B**, mean Adjusted Rand Index (ARI) over all tested parameter combinations for each algorithm on simulated data. Equivalently, for experimental data the **C**, maximum and **D**, mean ARI are shown with the best performing algorithms highlighted in red. Only successfully completed runs are considered. A more detailed description of the simulation parameters can be found in the Methods section.

To minimize bias in our benchmarking process, we evaluated each algorithm across 36 parameter combinations. Additionally, we used the 2000, 4000 and 6000 most highly variable genes in our benchmarking, resulting in a total of 108 runs per algorithm and dataset. From these runs we evaluated the best parameter combination (Fig 5A and 5C) in order to further minimize the effect from the choice of parameters.

In order to evaluate the clustering performance on a known ground truth, we simulated 12 data sets of differing clustering difficulty using Splatter [39]. The base parameters of the data sets were based on either the PBMC3k or Zeisel Brain Data, and the probability for a gene to be differentially expressed in a cell as well as the magnitude of the differential expression was varied for different simulations. A more detailed description of the simulations can be found in the Methods Section “Simulated Data.” On simulated data, CAdir performs similarly to other clustering algorithms and even outperforms both RaceID modes and DivBiclust, which only shows good clustering on the two easiest simulated data sets (Fig 5A). For the majority of simulated data sets, CAdir obtains an ARI equal to or close to 1, indicating almost perfect correspondence with the ground truth labels. Determining the number of clusters automatically is a challenging task, but while the automated version of RaceID (RaceID_auto) performs considerably worse than the version with fixed *k*, CAdir is equally performant when determining the cutoff angle automatically (Fig 5A). The performance of CAdir, as well as all other tested algorithms, however drops on the two most challenging simulated data sets. Notably, CAdir is extremely robust to the choice of parameters: Using the mean ARI (Fig 5B) and NMI (S6A Fig) over all parameter combinations, CAdir on average outperforms all other algorithms except Seurat.

On experimental data, CAdir is consistently among the best performing algorithms, and on three data sets (Darmanis, FreytagGold and BaronPancreas) even achieves the highest ARI (Fig 5C). On the majority of data sets, CAdir shows similar performance to the deep learning based clustering methods scG-cluster and scDeepCluster. However, these methods require much larger computational resources to run and train (see S7A Fig). In terms of the mean ARI, CAdir outperforms most other clustering on the smaller to medium-sized data sets, on which CAdir_auto obtains the best clustering result for 3 out of 6 data sets. CAdir on average also shows similar, if slightly lower, mean ARI scores compared to the best scoring algorithm CAbiNet on the three larger data sets (Fig 5D). Notably, only CAdir, CAbiNet, Seurat, kmeans and scDeepCluster achieve acceptable clustering results for the two largest data sets, Brain Organoids and Tabula Sapiens. CAdir is able to robustly cluster these large data sets, whereas they often lead to crashes for the algorithms SIMLR, SC3, RaceID and DivBiclust (S6B Fig). In fact, CAdir is the fastest of the tested clustering algorithms that clusters all cells, and is able to cluster 600 000 cells in approximately 30 min (S7A Fig, see also Supplementary Section “Runtime Comparison” in S1 Appendix). If CAdir tries to infer the cutoff angle from the data (CAdir_auto), the maximum achieved ARI is approximately equal to the runs with a predefined angle (Fig 5C). In some cases, such as the Tirosh data set, it even exceeds the version with a fixed angle.

Although CAdir does not achieve the highest ARI on the three largest data sets (Brain Organoids, Tabula Sapiens and Dmel Spatial) as well as the Tirosh data set, it is still one of the best performing algorithms on the three larger data sets. We further investigated why CAdir’s performance dropped on these 4 data sets. As can be seen in S8A, S9A and S9B Figs, CAdir clusters the Tirosh data into 5 clusters without any splits or merges of the initial cluster number. In comparison to the ground truth annotation, CAdir finds one cluster less. More specifically, CAdir clusters the related cancer-associated fibroblast and endothelial cells clusters together, but divides the largest cluster, the T cells, into two separate clusters (cluster 3 and 4). The very small NK cluster in the reference annotation is subsumed partially by both cluster 3 and 4 (see S9A and S9B Fig). As the majority of cells in the data set are T cells, this leads to the poor clustering performance as measured by the ARI (0.43) in our benchmarking. S10A and S10B Fig show the 4 genes with the highest $S_{\theta }$-score for cluster 3 and 4. The highest ranked genes show large differences in their expression between the two clusters, and in particular cluster 3 shows a higher expression of both *CD8A* and *CD8B* compared to cluster 4. Cluster 4 on the other hand shows much higher expression of *CD4* compared to cluster 3 (S10C Fig), indicating that the two sub-clusters identified by CAdir are in fact CD8^+^ and CD4^+^ T cell populations. This is further supported by the expression of the co-clustered *IL7R* in cluster 4 (see S10B Fig), which is typically found in higher levels on CD4^+^ T cells compared to non-memory CD8^+^ T cells [40]. Although this clustering does not match with the reference annotation, it is clear from the co-clustered genes and the expression profiles that they are in fact CD4^+^ and CD8^+^ T cell subpopulations, demonstrating that CAdir can resolve fine-grained cluster structures in the data that might otherwise be missed.

The Brain Organoids data is clustered into 14 distinct clusters by CAdir, compared to the 17 annotated cell clusters in the reference annotation (S9C and S9D Fig). These final clusters are the result of several merging steps as can be seen in S8B Fig. Starting out from only 5 initial clusters, CAdir first splits the clusters into 24 sub-clusters to separate out the different cell types. These clusters are then merged into similar clusters. For the Schwann cells and Neuroendocrine cells, CAdir repeatedly splits off groups of cells which are then merged to the same cluster in order to correctly sort the cells. Although the overall cluster structure seems to follow the clusters in the reference annotation (S9C and S9D Fig), CAdir does not correctly identify the split within the Cortical NSCs that can be observed in the UMAP. Although we followed the analysis suggested in the original publication, this split could potentially be due to uncorrected batch effects in the data (e.g., from the cell line or sequencing library) or a misannotation. Additionally, CAdir groups Cortical IPs and Cortical Neurons together, which fall along the same developmental gradient and therefore lack a clear separation. Similarly to the Tirosh data set, the misclustered cell types are also among the most numerous cell types in the data, leading to an ARI of 0.55.

The Tabula Sapiens data was initialized with 10 directions, which were split and subsequently merged analogously to the Brain Organoids data (S8C Fig). The data set contains 22 cell types, whereas CAdir identified 25 clusters (S9E and S9F Fig). Clustering on such large data sets is exceptionally difficult, as can be seen in the overall lower ARI scores obtained on this data set by other algorithms.

#### Parameter choices.

We studied the sensitivity of CAdir’s performance with respect to the settings of its parameters. An overview over all tunable parameters and their default values can be found in Table A in S1 Appendix (see also Supplementary Section “Tunable Parameters” in S1 Appendix). Like most other single-cell algorithms, CAdir retains a certain number of dimensions by way of dimension reduction. We investigated the influence of the number of CA dimensions retained. As shown in S11A Fig, for the majority of experimental data sets CAdir’s cell clustering performances quickly stabilizes with more than 30 dimensions kept, highlighting CAdir’s robustness to the choice of dimensionality. For a small subset of data sets, CAdir shows optimal performance in a smaller range of optimal dimensions, e.g., 40–55 dimensions for the Baron Pancreas data set. Because almost all tested data sets performed well between 30–50 dimensions, we recommend 50 dimensions as a starting point to an analysis, in line with the number of dimensions retained by default by other popular tools such as Seurat.

The number of clusters found by CAdir depends on the cutoff angle θ between cluster directions, which is set by a quantile cutoff in repeatedly randomized Association Plot data. S11B Fig shows that the precise choice for the quantile cutoff does not affect the clustering performance over a wider range of values. However, the performance slightly increases from the lowest tested value (0.7) up to the 0.99 quantile, for which the majority of data sets show the best performance. If the quantile is increased even further, a drop in performance can be observed for most data sets (Baron Pancreas, Zeisel, Darmanis, Dmel Spatial), indicating that a too restrictive quantile might yield a too small cutoff angle resulting in overclustering (S11B Fig). For this reason, CAdir’s default quantile cutoff is set to 0.99.

In addition to the quantile cutoff, we tested the number of random Association Plot directions required for a robust estimation of the cutoff angle. The inferred cutoff angle quickly stabilizes within only 50–100 random directions computed (S12 Fig and Supplementary Results Sections “Number of Random Directions For Cutoff Inference” in S1 Appendix). As measured by the achieved ARI, in many cases already 20 random directions achieved indistinguishable results to a cutoff angle estimated using more random directions (S12 Fig). To ensure a robust estimation of the cutoff angle, CAdir therefore uses 100 random directions by default. As shown in S11 and S12 Figs, CAdir shows good clustering results over a large range of parameter choices for the dimensionality, the quantile cutoff and number of random directions for the cutoff angle estimation, demonstrating the robustness of CAdir to the choice of parameters. A more detailed discussion can also be found in the Supplementary Results sections “Choice of Dimensionality,” “Choice of Angle Quantile Cutoff” and “Number of Random Directions For Cutoff Inference” in S1 Appendix.

The number of split-merge iterations for CAdir show only a minor influence on the final clustering. Using more than 5 iterations was sufficient for the clustering to converge for nearly all tested data sets, as shown in S13A and S13B Fig and Supplementary Section “Number of iterations” in S1 Appendix. Additional iterations did not improve the clustering results. CAdir by default only iterates until the cluster directions fall within 0.001 ° to the previous iteration and the maximum number of 50 iterations will therefore almost never be reached in practice.

CAdir performs feature selection by only assigning the 80 % of genes with the greatest distance from the origin to clusters. This ensures that housekeeping genes and other unspecific genes are filtered out. To demonstrate how the expression of genes in a cluster at different distances from the origin looks like, we plotted 5 approximately evenly spaced out co-clustered genes for the B cell and Endothelial cell cluster from the Tabula Muris Limb Muscle data. As shown in S14 Fig, the cluster specificity decreases the lower the distance from the origin and $S_{\theta }$-score are. Genes located close to the 80 % cutoff, which corresponds to a vector norm of 1.63 for the Tabula Muris Limb Muscle data set, are not specific for any cluster. CAdir’s cutoff therefore reduces the number of co-clustered genes to a more easy to interpret number, while conserving the cluster specificity of the co-clustered genes. A more in depth discussion can be found in Supplementary Section “Selecting Cluster Specific Genes” in S1 Appendix.

By sampling cell type clusters from the Tabula Muris cell atlas [2,41] we tested the ability of CAdir to recover the correct number of clusters in the data. Independent of the initialization, the number of clusters provided by CAdir follows well the number of sampled cell types up to 20 clusters, when batch effects from the tissue the cells originate from decrease the clustering quality (S7B Fig, see also Supplementary Results). In comparison, Seurat’s graph clustering approach tends to overestimate the number of clusters, especially when less than 20 cell types are sampled and a low number of Nearest Neighbors is used to construct the graph (S15 Fig). CAdir therefore is able to more accurately estimate the number of clusters on small to medium-sized data sets, whereas Seurat obtains better estimates on very large data sets with many cell types. Although neither method perfectly captures the correct number of clusters, CAdir allows the user to inspect the obtained cluster directions in an Association Plot, revealing visible sub-directions and making it easy to assess whether finer or coarser clustering is required. CAdir does not account for batch effects during clustering and therefore any batch effects present have to be removed using the preferred batch effect removal method prior to proceeding to clustering.

### Biclustering benchmarking

To better benchmark the accuracy of the co-clustering of the marker genes, we additionally compared CAdir to 8 other biclustering algorithms, namely CAbiNet, BackSPIN, CCA, Plaid, QUBIC, QUBIC2, s4vd and NMF [14,16,17,21,42–45]. Even though they are not biclustering algorithms in the traditional sense, we additionally added Seurat and Monocle3 to the benchmarking in order to better compare to other popular analysis workflows for scRNA-seq data in R. For the purpose of our benchmarking, we treated the clustering by Seurat and Monocle3 with subsequent marker gene detection as a biclustering. Because we know the differentially expressed genes for each cluster in the simulated data, we can evaluate how well the biclusters consisting of cells and genes correspond to the ground truth biclustering using the clustering error (CE) [46,47]. The benchmarking conditions were kept the same as for the comparison between cell clustering algorithms, but the results were additionally evaluated to assess the gene clustering.

**Fig 6:**
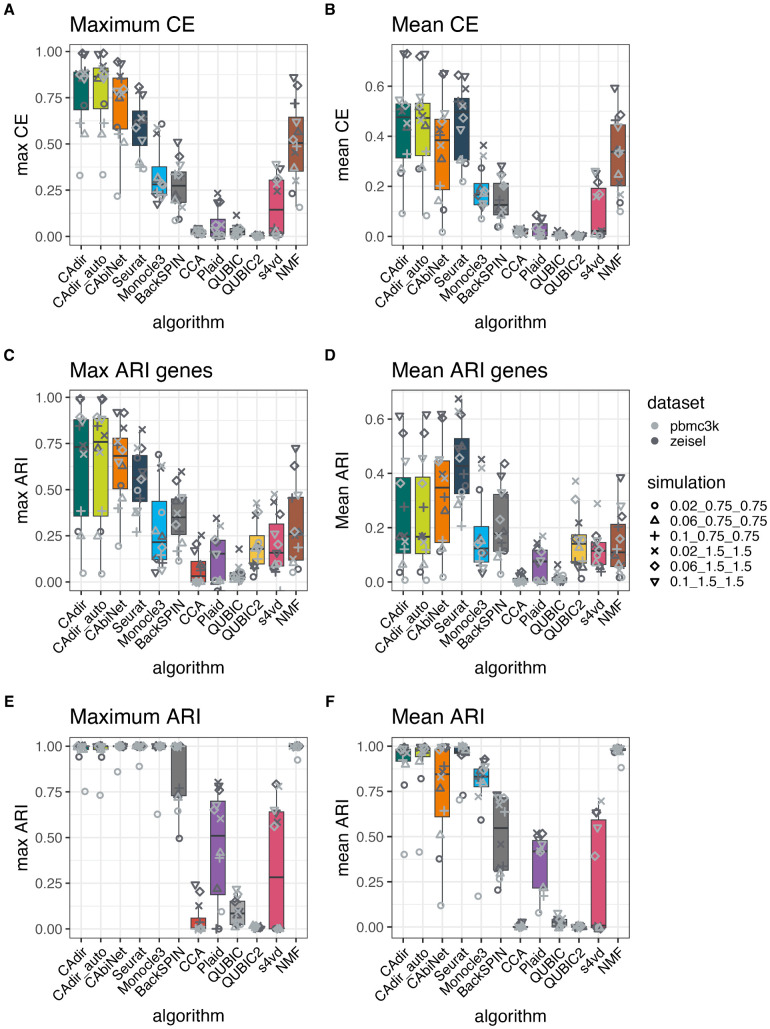
Biclustering benchmarking. Biclustering results for the simulated data sets as measured by the maximum **A**, Clustering Error (CE) and **C**, Adjusted Rand Index (ARI) of the gene clustering as well as the mean **B**, CE and **D**, ARI of genes. The cell clustering results are evaluated using the **E**, maximum and **F**, mean ARI.

Compared to the other biclustering algorithms, CAdir achieves both the best maximum and average CE (Fig 6A and 6B). Although their achieved CE scores are lower compared to CAdir, both CAbiNet and NMF are able to achieve good biclustering results (Fig 6A and 6B). However, when also taking cell clustering algorithms into account, on average Seurat obtains slightly better CE scores than CAdir, while CAdir outperforms Seurat on the two data sets with the strongest differences between clusters. Similarly, among the biclustering algorithms CAdir achieves the highest maximum Adjusted Rand Index (ARI) for the gene clustering (Fig 6C). Seurat and CAbiNet on average obtain a better ARI for the genes over all tested parameter combinations, while BackSPIN achieves similar results to CAdir on average (Fig 6D). This can be partially attributed to the fact that both Seurat and CAbiNet include methods to remove unspecific genes from the biclustering, whereas CAdir will always assign a gene to a cluster.

Lastly, we evaluated the cell clustering accuracy of the tested algorithms. As shown in our cell clustering, CAdir, CAbiNet, Seurat, Monocle3 and NMF achieve almost perfect maximum ARI scores (Fig 6E), whereas most other biclustering algorithms do not perform well. The only biclustering algorithm that exhibits similarly good performance to CAdir is NMF, while BackSPIN also shows good results, albeit lower than CAdir. When evaluating the mean ARI over all benchmarking runs (Fig 6F), CAdir, Seurat and NMF are the top performing algorithms. Overall, this demonstrates the ability of CAdir to recover the differentially expressed marker genes simultaneously to clustering the cells.

We additionally tested whether increased sparsity in the data has an effect on the clustering performance of CAdir. To this end we increased the sparsity of the simulated data by increasing the probability of genes to be a dropout (see S16 Fig and Supplementary Section “Benchmarking on Simulated Data with Increased Sparsity” in S1 Appendix). Although the cell clustering and biclustering performance of all tested algorithms dropped markedly, the overall picture remains the same. The benchmarking results for the cell clustering on the sparser simulated data are shown in S17A and S17B Fig. The maximum and mean ARI is overall noticeably lower for all algorithms, particularly on more difficult simulated parameter combinations. DivBiclust is particularly affected by the increased sparsity and failed to cluster any of the data sets. Importantly, despite the lower mean ARI scores, CAdir_auto still outperforms all other cell clustering algorithms excluding Seurat. Similarly, for the biclustering the overall CE scores are lower on the sparser simulated data (S17C and S17D Fig), but the automatic version of CAdir is still the best performing biclustering algorithm as measured using the maximum and mean CE.

## Discussion

CAdir is a versatile clustering algorithm that aims to provide actionable information on clustering decisions and the quality of the results. Unlike many other popular scRNA-seq clustering algorithms, CAdir can be run in a fully automatic mode in which CAdir infers the cutoff angle θ by itself to determine the number of clusters instead of relying on user defined parameter choices. The angle between the cluster directions is useful beyond just determining whether a cluster should be split or merged: Unlike distances between clusters, the angles between clusters provide an interpretable indicator of similarity that helps to identify similar cell types. Combined with its competitive clustering performance and its fast runtime, CAdir is ideally suited for the analysis of single-cell RNA-seq data. While CAbiNet achieves better cell clustering results than CAdir on large experimental data sets, it is unable to cluster data sets with more than 80 000 cells due to memory constraints. In comparison, CAdir clusters 600 000 cells in the same time required for CAbiNet to cluster 80 000 cells. CAdir is therefore able to cluster very large data sets in a fraction of the time required for other popular tools such as CAbiNet, SC3 or SIMLR.

The concept of clustering by direction intuitively lends itself to visualizing the clustering results in Association Plots, which provide a two-dimensional visualization of the cluster based on its direction in CA space. This method allows for a more direct evaluation of a cluster’s quality due to its interpretable axis compared to, e.g., UMAP or CAbiNet’s biMAP, which might distort the data in unforeseen ways. Beyond just providing a straightforward and intuitive way to judge a clusters quality, CAdir provides a visualization of the clustering decisions made by the algorithm in the form of a graph tree. The graph view of the splits and merges further facilitates the process of understanding if a cluster appropriately represents a distinct community of cells. Beyond improved visualizations, CAdir also elegantly circumvents a long-standing problem in the clustering field. Because clustering forces separation in the data, subsequent marker gene detection using the same data can find statistically significant genes even in the absence of any true clusters in the data. CAdir solves this problem, colloquially known as data-snooping, by detecting the marker genes simultaneously to the cell clusters without any additional computational cost.

In many cases the ground truth cell type annotation for experimental data is based on a clustering, thereby biasing our benchmarking towards popular methods such as k-means clustering or Seurat. Despite this, CAdir shows excellent performance on experimental data sets, showing similar or better performance to popular tools such as Seurat or k-means.

Unlike other algorithms that only provide a clustering of the cells, CAdir also assigns genes that are highly associated with a group to the same cluster. This not only allows researchers to understand which genes define a specific cluster, but enables fast cell type annotation without the need for differential gene expression analysis or other methods. Compared to other biclustering algorithms, CAdir outperforms all other tested algorithms as measured by the maximum CE and ARI on simulated data. Only Non-negative Matrix Factorization (NMF) achieves a similarly good cell clustering quality, but shows worse performance when taking the gene clustering into account too. Importantly, the majority of genes co-clustered by CAdir are shown to be known cell type marker genes, which allows for automatic cell type annotation based on the biclusters.

As a purely linear method, CAdir can struggle to find well fitting lines in the presence of strong non-linearities in the cluster structure. This could for example be the case when analyzing developmental data with many differentiating cells. In our benchmarking we have two such data sets, Brain Organoids and the Drosophila spatial data (Dmel Spatial) but in our analysis, CAdir performs en par or even outperforms other clustering algorithms such as Seurat or CAbiNet. Future iterations of the algorithm could be improved by allowing for non-linear cluster shapes through, e.g., kernel methods.

In summary, CAdir is a fast and capable clustering algorithm that provides interpretable output and can automatically infer the number of clusters from the data. The R package CAdir can be installed from GitHub: https://github.com/VingronLab/CAdir.

## Materials and methods

### Correspondence analysis

Correspondence analysis (CA) is a matrix embedding method similar to PCA. As described by Greenacre [27], the contingency table **P**, which represents the empirical probability that a gene *i* is expressed in cell *j*, is derived by dividing each element of the preprocessed m×n input matrix *X* with *m* genes and *n* cells by the total sum *n*_++_ of all entries:


$$\begin{array}{cc} n_{++} & =\sum_{i}\sum_{j}X_{i,j}\\ 𝐏 & =\frac{1}{n_{++}}X \end{array}$$ (1)

Then, the Pearson residuals **S** are calculated as the deviation of the expected probability $e_{ij}$ from the observed probability $p_{ij}$:


$$\begin{array}{c} r_{i}=\sum_{j}p_{ij},\;c_{j}=\sum_{i}p_{ij} \end{array}$$ (2)

$$\begin{array}{c} e_{ij}=r_{i}c_{j} \end{array}$$ (3)

$$\begin{array}{c} s_{ij}=\frac{p_{ij}-e_{ij}}{\sqrt{e_{ij}}} \end{array}$$ (4)

The Pearson residuals are then decomposed using Singular Value Decomposition (SVD):


$$\begin{array}{c} 𝐒=UD_{\alpha }{𝐕}^{T} \end{array}$$ (5)

#### Scaling of the coordinates.

In order to obtain interpretable relationships between cells and genes in CA, it is necessary to scale the singular vectors correctly [21]. The standard coordinates are the singular vectors weighted by the row or column masses. With ${𝐃}_{r}=diag(r)$ and ${𝐃}_{c}=diag(c)$ the standard coordinates for genes Φ and cells Γ are therefore:


$$\begin{array}{cc} \Phi & ={𝐃}_{r}^{-\frac{1}{2}}U,\\ \Gamma & ={𝐃}_{c}^{-\frac{1}{2}}V \end{array}$$ (6)

Scaling the standard coordinates furthermore by the singular values, we obtain the principal components **G** of cells and **F** of genes:


$$\begin{array}{cc} 𝐅 & =\Phi {𝐃}_{\alpha },\\ 𝐆 & =\Gamma {𝐃}_{\alpha } \end{array}$$ (7)

Scaling the cells in standard coordinates Γ and genes in principal coordinates **F** provides us with a so-called *asymmetric map*, a joint display of cell and gene points with interpretable distances. Using this scaling, the closer a gene is located to a cell the more associated, or specific, it is for a given cell [21].

### Clustering by directions

Before clustering the data with CAdir, it must first be processed using correspondence analysis (CA). Typically, after performing CA, only the first *d* dimensions with the highest inertia are retained to minimize noise and reduce the data set’s size. A key property of CA that CAdir is built upon, is that CA arranges cells belonging to similar groups along a direction in the dimension reduced space. CAdir capitalizes on this feature by clustering together cells and genes that lie along the same direction (see Fig 2A). The algorithm consists of three discrete steps that are alternatingly performed during clustering: Dirclust, Split and Merge. After the initial clustering (Dirclust), CAdir attempts to improve the number of clusters in the data based on the angles between clusters during the Split and Merge steps. The cutoff angle required for the split and merge steps is automatically inferred from the data by CAdir, but can alternatively also be set to a user defined value. CAdir repeats the cluster/split/merge loop several times to ensure that the clustering directions do not change anymore. Lastly, based on the final cluster directions the cluster specific genes are similarly assigned to the closest direction, allowing for cell type discovery and annotation of the clusters. A short description of how to use CAdir can be found in the Supplementary Section “Overview over Software Functionality” in S1 Appendix.

#### Detailed description of the algorithm.

First during the Dirclust step the n×d matrix **G** of principal cell coordinates in the *d* dimensional CA space is clustered into *k* initial clusters based on their directions in CA space (see Fig 2A). The *k* cluster directions thereby form a $L_{k\times d}$ matrix, indicating the directions along which the clusters lie in the dimension reduced space. In order to find the shortest orthogonal distance to a direction, the n×k distance matrix *D* is calculated (Alg. 1, line 8) and each cell is assigned to the closest direction $L_{k•}$ if the projection of cell $G_{i•}$ onto direction $L_{k•}$ is larger or equal to 0 (Alg. 1, line 12 and in Fig 2B). The cluster assignments are stored in the vector *c*.

Given the new cluster members, the cluster directions need to be updated. Total least squares regression is used to find the line that minimizes the orthogonal distance of the cells to the line (Fig 2A). This can be achieved efficiently by performing SVD on the cells in a cluster. The best fitting line corresponds to the first principal axis $V_{1}^{T}$ (Alg. 1, line 15 and following line) [48]. Importantly, in order to ensure that points lying on the same line, but opposite directions are not clustered together, the sign of the principal axis is flipped to point into the same direction as the majority of cells in the cluster if the weighted sum *s* in Equation 8 is smaller than 0 [49].


$$\begin{array}{c} s=\sum_{n}\langle G_{n•},L_{k•}^{T}\rangle^{2}sign(\langle G_{n•},L_{k•}^{T}\rangle ) \end{array}$$ (8)

In the pseudocode this is referred to as the flip_sign function. The cluster directions are updated until the angle between the directions of the current and the previous iteration do not differ by more than 0.001 ° (check_convergence in Algorithm 1 line 18), or alternatively for a user specified number of repetitions (max_r in Algorithm 1 line 3, usually around 10 iterations) (see Fig 2C).

**Algorithm 1 Dirclust**



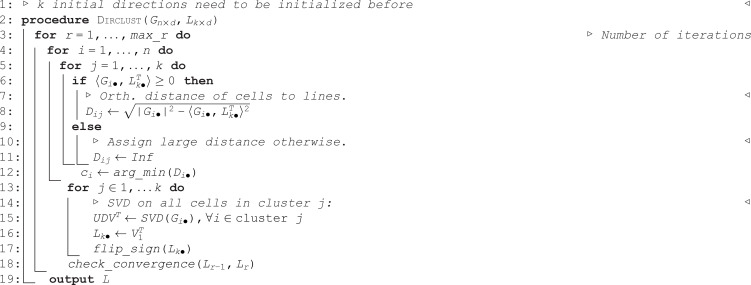



During the Split step each cluster of cells is split into two sub-clusters using Dirclust with *k* set to two (line 6 in Alg. 2). If the angle of the directions of the sub-clusters is above the pre-defined cutoff angle θ, the original cluster is split and the two new sub-clusters are used subsequently, otherwise the original cluster is kept (see Fig 2D). In order to ensure that a cluster can be split multiple times, the Split function is called recursively after each successful split until all possible splits have been performed (Alg. 2, line 10). Then, the newly split clusters are refined again by calling Dirclust until convergence.

**Algorithm 2 Split**



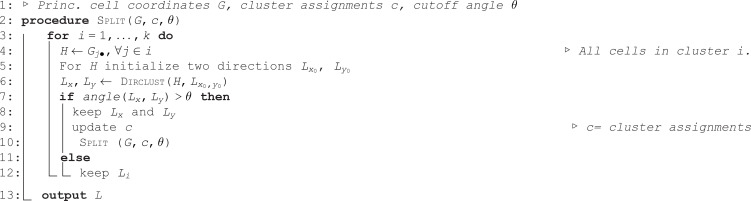



Given, that some of the split-off clusters might actually be part of a larger cluster, CAdir merges clusters during the Merge step. First, the pairwise angles between all directions in *L* are calculated (Alg. 3, line 6). If two or more directions have an angle smaller or equal to the cutoff θ, the cells are grouped in a single cluster and a new cluster direction is calculated by determining the first principal axis through SVD (Alg. 3, line 9–14 and Fig 2D). Similar to the Split step, Merge is called recursively after each successful merge to ensure all necessary merges are performed. Finally, the thus newly created clusters are then again refined by calling Dirclust.

**Algorithm 3 Merge**



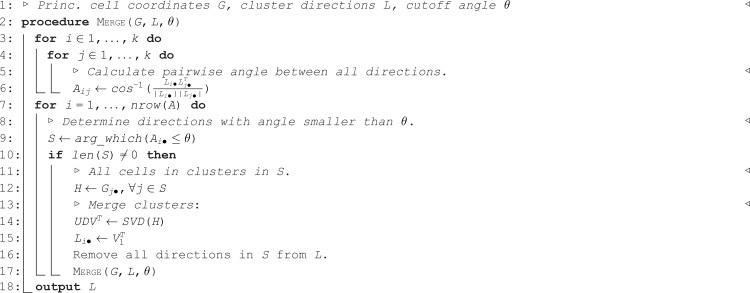



In summary, CAdir can be written as alternating Dirclust, Split and Merge steps as shown in Alg. 4. The initialization (Alg. 4, line 3) of the initial directions *L*_0_ is done by default through k-means_++_ initialization [50], but the directions can alternatively be initialized randomly. An evaluation of the effect of the initialization methods on the final clustering can be found in the Supporting Information S1 Appendix Section “Effect of the Initialization Method” and S18 Fig. After an initial clustering with Dirclust, the directions are split and merged for *r* repetitions until convergence. CAdir iterates until the all angles between the directions of the previous iteration and the new directions are equal to or smaller than a threshold (0.001 °) or up to a maximum number of max_n=50 cycles (Algorithm 4 line 5 and line 10). Similar to the main algorithm, each Dirclust sub-step iterates until convergence or until the maximum number of max_r iterations is reached. For the initial Dirclust step max_r is set to a maximum of 50 iterations. After each Split and Merge step, an additional Dirclust step is added, with a maximum of only 10 iterations, to refine the cluster directions and to ensure a good fit. As shown in S13C and S13D Fig, Dirclust converges quickly and typically no more than 10 iterations are required. In our testing, for all tested data sets no more than 5 iterations were needed for CADIR to achieve a stable clustering results (see also S13A and S13B Fig and Supplementary Results Section “Number of Iterations” in S1 Appendix). The convergence is checked both for each Dirclust step individually, and after each split-merge iteration. The final result is the matrix *L*, whose number of rows (directions) is determined by the algorithm. The cluster assignments are then simply the closest direction for each cell. An overview over the tunable parameters of CAdir can be found in Table A in S1 Appendix.

**Algorithm 4 CAdir**



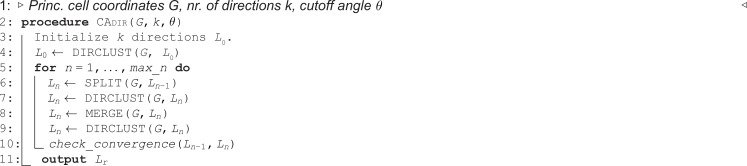



### Automatic cutoff angle inference

CAdir determines the cutoff angle θ for splitting and merging of clusters directly from the data. The angle is inferred by determining the angle above which the majority of cells unrelated to a cluster would fall in an Association Plot [51]. In this publication, this number is determined by many times randomizing the entries in each row (genes) of the input matrix *G* and determining the angle that delineates 99 % of the data. Since this is very time-consuming, we have implemented a heuristic which closely approximates the result of this randomization. We select randomly at least 100 directions from the origin and compute the corresponding (random) Associations Plots. Note that due to the random choice of direction we do not expect any meaningful cluster to lie in this direction. The cutoff angle is then set to the angle above which 99 % of the data lie. Typically, this angle will be between 60 ° and 65 °. In the Association Plot one can sometimes observe that related clusters lie within a much smaller angle. As shown in S19 Fig, the difference between the angle inferred by the permutation method and the method based on randomized directions is negligible. CAdir therefore uses by default the random directions method in order to speed up the inference.

#### Association plots.

In CA space the cells of a cluster and their specific genes lie along a direction and can be visualized in the form of an Association Plot [30]. The Association Plot is a two-dimensional plot summarizing the position of cells and genes with respect to a direction in space, typically the direction representing the cell cluster. A point represents either a cell or a gene. The x-coordinate (axis labelled as association strength) of a point in the Association Plot is the distance from the origin to the orthogonal projection of the point onto the direction. The y-coordinate (axis labelled as orthogonal distance) is simply the orthogonal distance of the point to the direction. For illustration refer to Fig 2A. There the points in the (high-dimensional) CA space are shown together with their orthogonal projections onto the direction of each of the three clusters. One Association Plot would describe one cluster, with the respective distance to the projection on the x-axis and the orthogonal distances in the y-axis.

Association Plots are constructed as described by Gralinska et al. [30]. The Association Plot x-coordinate of a point *a* is calculated as the scalar projection of its vector $\vec{b}$ in CA space onto the cluster direction $\vec{d}$, whereas the y-coordinate is the distance of the point to the direction:


$$\begin{array}{cc} a_{x} & =\frac{\vec{b}\vec{d}}{\left\|\vec{d}\right\|},\\ a_{y} & =\sqrt{\|\vec{b}{\|}^{2}-a_{x}^{2}} \end{array}$$ (9)

The value of Association Plots lies in the interpretability of cluster quality and the relationship between clustered cells and their specifically expressed genes. A well-defined cluster of cells will form a cloud of points extending near the x-axis to the right. Likewise, the cluster specific genes will lie towards the right and close to the x-axis. This is a reflection of the feature of correspondence analysis whereby a cluster of cells with its specific genes extends along a direction away from the origin. Conversely, when a cluster is of low quality, its cells will remain close to the origin and will not be pointed to the right, or they will point upwards rather than follow the x-axis. Correspondingly, there will be few genes to the right and close to the x-axis. Generally, the higher the y-coordinate, the less specific for a cluster the cell or gene is.

Since CAdir clusters the data into directions with associated cells and genes, we utilize the respective Association Plots to visualize clustering results. When CAdir produces *k* clusters, we output *k* Association Plots. Importantly, because the cluster directions are determined using the principal coordinates, they are best visualized in an Association Plot together with the cells using the principal coordinates. However, when visualizing cells and genes simultaneously, a better interpretability of the Association Plot is achieved when displaying cells in standard coordinates and genes in principal coordinates. This choice of coordinates can lead to slight differences in the visualizations. CAdir offers many additional customization options when plotting the cluster Association Plots. In S20 Fig we demonstrate some of the available parameters.

### Gene assignment

In CA, cluster specific cells and genes are located along the cluster direction in an asymmetric map, and they can therefore be assigned to the cluster based on the orthogonal distance to the cluster direction. The most specific genes lie far away from the origin while around the origin all the unspecific genes are located. Therefore, we filter out those genes that are within a sphere of radius *r* around the origin. The radius *r* is chosen to filter out 80% of genes. Only the remaining genes get assigned to the cluster direction with the shortest distance of the gene’s standard coordinates to its projection on the line. We found that the genes are best filtered based on their principal coordinates, but the actual cluster assignment is done in standard coordinates in order to ensure interpretable distances.

### Gene scoring

After performing a clustering of cells and genes, it is often of interest which genes are the most specific for a cluster. However, only ranking genes by their association to a cluster would only consider the x-axis of an Association Plot and therefore ignore potential associations to other clusters. In order to quantitatively score how specific genes are for a cluster, the $S_{\theta }$-score, introduced by Gralinska et al. [30,51] can be used.

Given a point $c_{i}$ with Association Plot x-coordinates $x_{c_{i}}$ and y-coordinate $y_{c_{i}}$, the $S_{\theta }$-score can be calculated as follows:


$$\begin{array}{c} S_{\theta }(x_{c_{i}},y_{c_{i}})=x_{c_{i}}-\frac{y_{c_{i}}}{\tan\theta } \end{array}$$ (10)

Because θ determines the angle of the line above which 99 % of the cluster unspecific genes fall, a $S_{\theta }$-score of 0 would mean the gene falls exactly on this line. Points to the right and below of this line will have a positive score, whereas points to the left and above will have a negative score. Shifting this line in parallel by one unit on the x-axis, gives the line for points with a score of 1 and similarly shifting it by two units gives the line for points with a score of 2. A positive score larger than 0 therefore indicates cluster specificity, as the points fall to the right of the line above which 99 % of cluster unspecific genes fall. S20E and S20F Fig show Association Plots with the line defined by the cutoff angle plotted.

### Cell type annotation

The co-clustered genes can then be used as input for a gene set overrepresentation analysis using the CellMarker 2.0 [52] gene set to annotate the clusters. Using the CellMarker 2.0 [52] gene set, each cluster is annotated as follows: The gene sets are filtered to only contain sets with a minimum of 10 and a maximum of 500 genes and only genes that are present in the single-cell data set and could therefore be assigned to a cluster are kept. A hypergeometric test is then performed for all co-clustered genes in a cluster and all gene sets. Obtained p-values are adjusted to correct for multiple testing and the adjusted log-p-values are used to construct a cost matrix containing the clusters and all enriched gene sets. If a cluster’s genes were not found to be enriched in a specific cluster, its cost gets set to 0. Using the R package RcppHungarian (https://doi.org/10.32614/CRAN.package.RcppHungarian), we applied the Hungarian method [53] to solve the assignment problem, such that each cluster is getting uniquely assigned a cell type, even if clusters have similar enriched gene sets. If, however, the assigned gene set has an adjusted hypergeometric p-value smaller than 0.05 the cluster is left unannotated.

Alternatively to the Cell Marker gene set, the cells can also be annotated using the PanglaoDB [54] gene set. The CAdir R package also includes the option to annotate the data by using the $S_{\theta }$-scores of the genes to perform gene set enrichment analysis (GSEA) [55,56] as an alternative to gene set overrepresentation analysis.

### Data processing

Simulated and experimental data sets used for the benchmarking were pre-processed as follows: Using the functions perCellQCMetrics and perCellQCFilters from the R package scuttle [57], we removed cells that are identified as outliers based on the number of detected genes, the sum of counts per cell and the percent of mitochondrial reads. Additionally, we removed genes that are expressed in less than 1 % of cells. Afterwards, cells were normalized and counts were transformed to log-counts using the functions quickCluster, computeSumFactor and logNormCounts from the packages scran [33] and scuttle [57].

The Brain Organoids data was pre-processed similar to the other data sets, but as was also done in the original publication [58], only cells with less than 40 % mitochondrial counts were kept.

### Simulated data

Simulated data was generated using the Bioconductor package Splatter [39], which can generate new single-cell RNA-seq data with arbitrary number of clusters based on parameters estimated from experimental data. We used two different data sets, the Zeisel Brain Data [14] (Zeisel) obtained through the Bioconductor package scRNA-seq [59] and PBMC3k data from 10x Genomics obtained through the Bioconductor R package TENxPBMCData, [29] to estimate parameters concerning the mean gene expression, outliers, library size, the biological coefficient of variation and dropouts.

We used each real data set (Zeisel and PBMC3k) as the basis for 6 different simulated data sets for which we varied the probability for a gene to be differentially expressed as well as the shape and mean parameter of the log-normal distribution that defines the magnitude of the differential expression, resulting in a total of 12 data sets (see Table 1). Each simulated data set was generated with 1,000 cells, 10,000 genes and 6 six clusters. The generated clusters contained 30 %, 25 %, 20 %, 10 %, 10 % and 5 % of the total number of cells respectively.

**Table 1:** Parameter combinations for simulated data used for benchmarking. The 12 simulated data sets used in benchmarking the cell clustering are either based on the PMBC3k or Zeisel data set. For each reference data set we estimated the base parameters and then varied the mean of the log-normal distribution (DE factor mean), the variance of the log-normal distribution (DE factor var.) and the probability for a gene to be differentially expressed (DE prob.).

DE prob.	DE factor mean	DE factor var.	Name
0.02	0.75	0.75	(pbmc3k / zeisel)_0.02_0.75_0.75
0.06	0.75	0.75	(pbmc3k / zeisel)_0.06_0.75_0.75
0.1	0.75	0.75	(pbmc3k / zeisel)_0.1_0.75_0.75
0.02	1.5	1.5	(pbmc3k / zeisel)_0.02_1.5_1.5
0.06	1.5	1.5	(pbmc3k / zeisel)_0.06_1.5_1.5
0.1	1.5	1.5	(pbmc3k / zeisel)_0.1_1.5_1.5

### Experimental data

Experimental data sets were downloaded directly from the relevant publications or alternatively obtained through R data repositories such as the scRNAseq and TENxPBMCData Bioconductor packages. In our benchmarking we used the following data sets: Freytag Gold [60], Tabula Muris [41], Tirosh [61], PBMC10x [62], Dmel Spatial [63], Tabula Sapiens [64] and Brain Organoids [58] data sets were downloaded from the resources specified in the respective publication. Using the Bioconductor package scRNAseq [59] we downloaded the following data sets: Zeisel Brain [14], Darmanis [65] and Baron Pancreas [66]. The Tabula Muris LM (Tabula Muris subsetted to Limb Muscle tissue) [41] data was downloaded using the TabulaMurisData R Bioconductor package [28] and the PBMC3K data was downloaded using the TENxPBMCData Bioconductor package [29]. See also Table 2 for an overview of the data sets. All data sets were pre-processed as described in the Section Data Processing.

**Table 2:** Overview over the used data sets. The name listed under Data Set is used as the shorthand name for the data set. Cell and gene counts refer to the pre-processed data before feature selection.

Data Set	cells × genes	Description
Darmanis	461 × 17 533	Human adult cortical tissue
Freytag Gold	914 × 19 973	Human lung adenocarcinoma cell lines
Tabula Muris LM	1882 × 13 204	Mouse limb muscle tissue
Zeisel Brain	2874 × 14 508	Mouse cortex and hippocampus
PBMC3k	2700 × 32 738	Human PBMCs
Tirosh	2880 × 16 347	Human melanoma
PBMC10x	3176 × 11 881	Human PBMCs
Baron Pancreas	8569 × 12 238	Human Pancreas
Dmel Spatial	14 808 × 7 178	*D. melanogaster* embryo
Tabula Sapiens	32 393 × 15 674	Human endothelial cells
Brain Organoids	35 291 × 10 640	Human cerebral organoids
Tabula Muris	15 771 × 44 104	Mouse tissue

#### Discussed data.

The Tabula Muris Limb Muscle and PBMC3k data sets discussed in the results were processed as following: We downloaded the Smartseq2 Tabula Muris data with the Bioconductor package TabulaMurisData [28] and subsetted it to the Limb Muscle tissue. It was processed as described above and then subset to the 6000 most highly variable genes using the R package scran, resulting in a data set with 1882 cells and 6000 genes. We performed CA using the package APL and kept the first 30 dimensions. Subsequently, the data was clustered using CAdir with *k* = 8 and the angle cutoff set to 55 degrees. Cells were annotated by gene set overrepresentation analysis using the CellMarker 2.0 gene set.

The PBMC3k data was downloaded from [29] (https://doi.org/10.18129/B9.bioc.TENxPBMCData) and preprocessed according to the Seurat “Guided Clustering Tutorial” [67] (https://satijalab.org/seurat/articles/pbmc3k_tutorial, accessed on 2024-08-23). We retained the 20 % most highly variable genes, performed CA and kept the first 30 dimensions. Clustering with CAdir was performed with *k* = 12 and the cutoff angle was inferred automatically by CAdir. The co-clustered genes were used to annotate the data using the included gene set overrepresentation analysis method. To intentionally create a poor clustering to demonstrate CAdir’s ability to assess the quality of a clustering we clustered the PBMC3k data with *k* = 14 and the cutoff angle set to 55 degrees. This data set was also used for validating the marker genes as described in the Supplementary Section “Validation of Marker Genes” in S1 Appendix. An improved clustering used for the comparison to other marker gene detection methods was performed using the 1000 most highly variable genes, 20 CA dimensions with *k* = 7 initial directions and the cutoff angle θ set to 50. The 30 % least specific genes were filtered out based on their distance from the origin.

### Benchmarking

For the evaluation of the cell clustering performance, we benchmarked CAdir against 10 other clustering algorithms, namely Seurat [3], CAbiNet [21], Monocle3 [4], SIMLR [7], SC3 [37], RaceID [35,36], DivBiclust [38], k-means clustering, scDeepCluster [9] and scG-cluster [10]. Similarly, to evaluate the simultaneous clustering of cells and genes, we tested CAdir against 8 other biclustering algorithms: CAbiNet, BackSPIN, CCA, Plaid, QUBIC, QUBIC2, s4vd and Non-negative Matrix Factorization (NMF) [14,16,17,21,42–45]. Seurat [3] and Monocle3 [4], in conjunction with marker gene detection, were additionally added to the biclustering comparison.

To perform biclustering using NMF we used the RcppML package [45]. The log-count matrix is factorized into two matrices with *k* factors. Each cell and gene is then assigned to the factor with the highest coefficient. Because the factorization is not unique, we perform several restarts and return the best performing model as measured by the mean-squared-error. Additionally, L1-regularization is applied in order to increase the sparsity of the returned matrices. The number of factors *k*, number of restarts and the L1-regularization penalties are varied during the benchmarking (see Table D in S1 Appendix).

In order to test the algorithms under different conditions, we benchmarked them on the simulated and real data sets using the 2000, 4000, and 6000 most highly variable genes as determined by the function modelGeneVar and getTopHVGs from the package scran [33]. To prevent any bias in the choice of parameters, we additionally ran every algorithm with 36 different parameter combinations for a total of 108 runs per algorithm and data set. As the number of parameters differ between algorithms, this can result in many variations of a single parameter or only a few different choices for more parameters. Table C and D in S1 Appendix list the 36 parameter combinations used for the cell clustering (Table C in S1 Appendix) and biclustering (Table D in S1 Appendix) benchmarking respectively. The data was pre-processed in the same way for all algorithms prior to clustering. Algorithms that failed during the first run were rerun with more computational resources. If for example an algorithm required more memory than was provided, it was rerun with 500 Gb of memory, but if it exceeded the run time limits, the algorithm was restarted with a run time limit of 24 hours. For any other failures the algorithms were rerun with double the amount resources as for the original run, with a maximum of 500 Gb of memory and 24 hours of runtime. Algorithms that did not finish the clustering were rerun up to two times.

#### Cell clustering evaluation.

We used the Adjusted Rand Index (ARI) [68] and the normalized mutual information (NMI) [69] as implemented in the R package aricode [70] to compare the cell clusterings to the reference annotations. The Adjusted Rand Index measures the similarity between two clusterings as done by the Rand Index, but is adjusted to correct for coincidental clusterings. The ARI ranges from -1–1, where 0 indicates a random clustering, -1 a clustering worse than expected by chance and 1 that the two clusterings match perfectly. As the name already implies, NMI is a normalized measure for mutual information, and is bounded between 0 and 1. As it measures the dependence between the two groupings, higher values for the NMI indicate a higher association between the two clusterings.

Only successful runs were used for the evaluation, meaning that the summary statics such as mean or the maximum are taken over differing amount of runs, depending on how many effectively finished. We furthermore excluded runs that did not return interpretable results. Some clustering algorithms for example return “NA” or 0 clusters when they do not converge. These results were therefore excluded.

#### Biclustering evaluation.

In order to evaluate the simultaneous clustering of cells and genes, we used the clustering error (CE), introduced by Patrikainen and Meila [46,47]. Unlike the ARI, the CE measures the proportion of cells and genes that are assigned to a wrong cluster in a biclustering *B*, compared to a reference biclustering $\hat{B}$. The clusters in *B* and $\hat{B}$ are optimally matched in advance and therefore the number of confused points between the two biclusterings is minimized. This is achieved using the Hungarian algorithm [53]. The CE is then given by the following formula:


$$\begin{array}{c} \mathrm{CE}(B,\hat{B})=1-\frac{|U|-d_{max}}{\left|U\right|}=\frac{d_{max}}{\left|U\right|}, \end{array}$$ (11)

where $U|=|B\cup \hat{B}|$ represents the number of entries shared between *B* and $\hat{B}$ and $d_{max}$ is the maximal sum of the confusion matrix. The CE ranges from 0 to 1, where a higher value reflects a better biclustering performance.

#### Runtime benchmarking.

Similarly to the benchmarking performed for the cell clustering, we used Splatter to simulate data sets of increasing size. We simulated data sets with 2 000 genes and 1 000, 10 000, 20 000, 40 000, 60 000, 80 000, 100 000, 200 000, 400 000 or 600 000 cells. For all simulated data sets we simulated 6 clusters and set the parameters de.facLoc and de.facScale to 1.5. The probability for a gene to be differentially expressed was set to 0.1. The simulated data sets were normalized using the functions quickCluster and logNormCounts. All tested algorithms were run using a single thread and the runtime including potential dimensionality reduction steps was recorded.

#### Cluster number recovery.

To benchmark how well CAdir recovers the correct number of clusters, we used a scheme similar to the one described by Yu et al. [2]: We subset the pre-processed Tabula Muris cell atlas data to cell types with at least 300 cells, resulting in a data set 15 771 genes × 39 207 cells with 38 cell types from 18 tissues. We then subset the data set to *n* randomly sampled cell types, where *n* is between 4–30 cell types in steps of 2, and again sub-sampled each cluster to 200 cells in order to equalize the size of the clusters. We retained the top 4 000 most highly variable genes, kept the first *n* + 20 dimensions after performing CA and let CAdir automatically infer the cutoff angle below which 99 %, 99.9 % or 99.99 % of cells fall when considering random directions in CA space. To account for the variability introduced by randomly sampling clusters, we repeated each run 10 times.

### Code availability

The latest version of CAdir can be downloaded and installed from GitHub (https://github.com/VingronLab/CAdir). The version of the package used to produce the results presented in this manuscript is available on Zenodo (https://doi.org/10.5281/zenodo.14900874). The code to reproduce the presented results can be found here on GitHub (https://github.com/VingronLab/CAdir_results) and is also deposited on Zenodo (https://doi.org/10.5281/zenodo.14894255).

## Supporting information

S1 Appendix Supplementary Materials and Results. Additional comparisons, analyses and benchmarkings that give additional insights into the performance and functionality of CAdir.
(PDF)

S1 Fig Interpretation of the Tabula Muris Limb Muscle data clustering. A, Matrix of the pairwise angles between the cluster directions. Lower angles indicate higher similarity between clusters. B, Each row shows an Association Plot of the respective cluster in the diagonal and lines that show the direction of the remaining other clusters projected into the Association Plot. C, Sankey plot of the annotated clustering obtained with CAdir (left) and the ground truth annotation (right). Association Plot with cells in standard coordinates for D, B cells and E, Endothelial cells. Closely associated clusters are colored in red and blue.
(PDF)

S2 FigClustering of PBMC3k data - additional plots.A, Association Plots of the corrected clustering of the PBMC3k data after removing outlier cells and genes. Clustered cells are colored in lime green and co-clustered genes in orange. Five genes with the highest Sθ-score are labelled. Other cells and genes are colored in dark green and grey respectively. B, Sankey plot comparing the corrected clustering (left) against the annotation obtained through the Seurat vignette (right).(PDF)

S3 FigProportion of known marker genes among co-clustered genes.Clustering of the PBMC3k data using CAdir. A-E, Proportion of co-clustered genes with Sθ-score > 0 that are also contained in the CellMarker gene set used to annotate the cluster. F, For cluster 6 the gene set corresponding to the corresponding cell type from the annotation was used. G, Sankey plot of the clustering to show correspondence to the reference annotation.(PNG)

S4 FigLog-count expression plots of top ranked genes by CAdir and scran.The violin plots show the expression for the 4 highest ranked genes using the $S_{\theta }$-score for CAdir and the mean AUC for scran. A-B, Expression for the B cell cluster. C-D, Expression for the Monocytes cluster. E-F, Expression for the Cytotoxic CD4^+^ T cell cluster.(PDF)

S5 FigOverlap of marker genes found by CAdir and scran.The overlap between the co-clustered genes found by CAdir and scran’s scoreMarkers is determined by taking the same number of top ranked genes for both methods. A, Overlap between CAdir and scran using all co-clustered genes and B, using only the co-clustered genes with $S_{\theta }>0$.(PDF)

S6 Fig Average NMI benchmarking results and percentage of successful runs per algorithm. A, Mean Normalized Mutual Information over all 108 parameter combinations for each simulated data set. B, Fraction of successful (dark green) runs, runs that produced N/A (not assigned, orange) and crashed runs (red) for each algorithm and tested data set.
(PDF)

S7 Fig Runtime and Cluster Detection. A, Computational runtime on simulated data of increasing size. Both x-axis and y-axis are in log10-scale for better interpretability. SC3 is marked in a dashed line as it is the only benchmarked cluster that does not cluster all cells in a conventional manner but instead only clusters 5000 cells and then attempts to assign the remaining cells to these clusters. B, Deviation of the number of retrieved clusters from the number of randomly sampled cell type clusters from the Tabula Muris cell atlas for different numbers of *k* used to initialize CAdir and different quantile cutoffs for the automatic angle determination (apl_q). Error bars indicate the standard deviation over 10 replicate runs.
(PDF)

S8 Fig Split-merge graph plots. For the cell clustering parameter combination that achieved the best ARI score, split-merge graphs are plotted for the A, Tirosh B, Brain Organoids and C, Tabula Sapiens data set.
(PDF)

S9 Fig UMAPs of CAdir clustering results. Using the best scoring parameter combination of the cell clustering comparison, UMAPs for both the CAdir clustering and the reference annotation are plotted for the A-B, Tirosh, C-D, Brain Organoids and E-F, Tabula Sapiens. These data sets were chosen to further understand why CAdir did not achieve the best clustering results in the benchmarking on these data sets.
(PNG)

S10 FigExpression of co-clustered genes for selected clusters - Tirosh data set.Expression levels of the 4 genes with the highest $S_{\theta }$-score are shown for A, cluster 3 and B, cluster 4 using the best performing parameter combinations from the cell clustering benchmarking of CAdir on the Tirosh data set. C, Expression of *CD4* in cluster 3 and 4.(PDF)

S11 Fig Influence of the choice of dimensionality and Association Plot cutoff quantile on the clustering results. Achieved ARI for A, the chosen number of CA dimensions and B, the Association Plot (APL) cutoff quantiles for experimental data sets.
(PDF)

S12 Fig Effect of the number of random directions on the inferred cutoff angle. A, Number of random directions in comparison to the inferred cutoff angle. B, Number of random directions compared to the achieved ARI. Overall, after approximately 100 iterations the inferred angle is stable and therefore a higher number of random directions does not change the resulting ARI meaningfully.
(PDF)

S13 Fig Effect of the number of CAdir iterations. Obtained cell clustering ARI on A, experimental data and B, simulated data for 1–50 CAdir iterations. The same comparison was also performed for C, experimental and D, simulated data for the Dirclust sub-step.
(PDF)

S14 FigExpression of selected genes with increasing cluster specificity.Association Plots of the A, B cell B, Endothelial cell clusters of the Tabula Muris Limb Muscle data. The selected genes increase in both their vector norm and $S_{\theta }$-score from left to right. The violin plots for the selected genes show the log-normalized expression of the genes in the clusters. As shown, the further out the gene lies from the origin, the more cluster specific they are.(PDF)

S15 Fig Number of recovered clusters using Seurat. Deviation of the number of recovered clusters using Seurat (y-axis) compared to the number of randomly sampled clusters (x-axis) from the Tabula Muris cell atlas. The deviation was measured for different numbers of nearest neighbors (NNs) as well as for different Leiden clustering resolutions.
(PDF)

S16 Fig Splatter simulation gene dropout probability. A, Logistic function with increased dropout probability. The simulation parameter dropout.mid that determines the point at which the probability for a gene to be a dropout is 0.5 is set to 20 and dropout.shape is set to -0.05 for a slower falloff. Logistic functions estimated from the data for b, the Zeisel and B, PBMC3k data set.
(PDF)

S17 Fig Benchmarking results on sparser simulated data. Biclustering benchmarking results for simulated data with increased gene dropout rate. The cell clustering is evaluated by A, the maximum ARI and B mean ARI of the cell clustering and the biclustering using the C, the maximum CE and D, the mean CE over all parameter combinations. Missing results for certain algorithms are because all (bi-)clustering runs for a data set either did not yield any interpretable results or all runs crashed (see, e.g., Plaid and DivBiclust).
(PDF)

S18 Fig Comparison of initialization methods for CAdir. Adjusted Rand Index for clustering using kmeans_++_ (kmeansppp) and random initialization (rand) over 100 repetitions on the Tabula Muris Limb Muscle data for either *k* = 8 (ground truth) or *k* = 5 (kmeanspp_sub and rand_sub).
(PDF)

S19 FigComparison of θ inference methods.For each experimental data set used in the benchmarking, the cutoff angle θ is inferred either using the permutation based method (“perm”) or using randomized directions (“rand”). Panel A shows the inferred angle, whereas B shows the ARI of the clustering result using the inferred angle.(PNG)

S20 FigOverview over plotting functionality.Association Plots for the B cell cluster of the Tabula Muris Limb Muscle data set. A, Cells are colored by their respective cluster and the T cell and Macrophage cluster directions are highlighted. B, Association Plot only showing the genes, with the top 10 most highly associated genes labelled. Depending on the main interest, the size of either C, the genes, or D, the cells can be increased to provide a better overview. Panel E shows the Association plot for the cells in principal coordinates. The red line has the same angle as the inferred cutoff angle θ. The clustered cells are clearly delineated by the cutoff line from other cell types. Similarly in F, cells and genes are plotted together. Genes that fall to the right and below the red cutoff angle line are the most cluster specific.(PNG)

## References

[1] Zhang S, Li X, Lin J, Lin Q, Wong K-C. Review of single-cell RNA-seq data clustering for cell-type identification and characterization. RNA. 2023;29(5):517–30. doi: 10.1261/rna.078965.121 36737104 PMC10158997

[2] Yu L, Cao Y, Yang JYH, Yang P. Benchmarking clustering algorithms on estimating the number of cell types from single-cell RNA-sequencing data. Genome Biol. 2022;23(1):49. doi: 10.1186/s13059-022-02622-0 35135612 PMC8822786

[3] Hao Y, Stuart T, Kowalski MH, Choudhary S, Hoffman P, Hartman A, et al. Dictionary learning for integrative, multimodal and scalable single-cell analysis. Nat Biotechnol. 2024;42(2):293–304. doi: 10.1038/s41587-023-01767-y 37231261 PMC10928517

[4] Cao J, Spielmann M, Qiu X, Huang X, Ibrahim DM, Hill AJ, et al. The single-cell transcriptional landscape of mammalian organogenesis. Nature. 2019;566(7745):496–502. doi: 10.1038/s41586-019-0969-x 30787437 PMC6434952

[5] Traag VA, Waltman L, van Eck NJ. From Louvain to Leiden: Guaranteeing Well-Connected Communities. Scientific Reports. 2019;9(1):5233. doi: 10.1038/s41598-019-41695-z 30914743 PMC6435756

[6] von Luxburg U. A tutorial on spectral clustering. Statistics and Computing. 2007;17(4):395–416. doi: 10.1007/s11222-007-9033-z

[7] Wang B, Ramazzotti D, De Sano L, Zhu J, Pierson E, Batzoglou S. SIMLR: A Tool for Large-Scale Genomic Analyses by Multi-Kernel Learning. Proteomics. 2018;18(2):10.1002/pmic.201700232. doi: 10.1002/pmic.201700232 29265724

[8] Zheng R, Li M, Liang Z, Wu FX, Pan Y, Wang J. SinNLRR: A robust subspace clustering method for cell type detection by non-negative and low-rank representation. Bioinformatics. 2019;35(19):3642–50. doi: 10.1093/bioinformatics/btz139 30821315

[9] Tian T, Wan J, Song Q, Wei Z. Clustering single-cell RNA-seq data with a model-based deep learning approach. Nat Mach Intell. 2019;1(4):191–8. doi: 10.1038/s42256-019-0037-0

[10] Zhang Y, Feng X, Wang Y, Shi K. Deep learning powered single-cell clustering framework with enhanced accuracy and stability. Sci Rep. 2025;15(1):4107. doi: 10.1038/s41598-025-87672-7 39900656 PMC11791198

[11] Theodoris CV, Xiao L, Chopra A, Chaffin MD, Al Sayed ZR, Hill MC, et al. Transfer learning enables predictions in network biology. Nature. 2023;618(7965):616–24. doi: 10.1038/s41586-023-06139-9 37258680 PMC10949956

[12] Baek S, Song K, Lee I. Single-Cell Foundation Models: Bringing Artificial Intelligence into Cell Biology. Experimental & Molecular Medicine. 2025;57(10):2169–81. doi: 10.1038/s12276-025-01547-5 41028523 PMC12586647

[13] Zhang JM, Kamath GM, Tse DN. Valid Post-clustering Differential Analysis for Single-Cell RNA-Seq. Cell Syst. 2019;9(4):383-392.e6. doi: 10.1016/j.cels.2019.07.012 31521605 PMC7202736

[14] Zeisel A, Muñoz-Manchado AB, Codeluppi S, Lönnerberg P, La Manno G, Juréus A, et al. Brain structure. Cell types in the mouse cortex and hippocampus revealed by single-cell RNA-seq. Science. 2015;347(6226):1138–42. doi: 10.1126/science.aaa1934 25700174

[15] Tsafrir D, Tsafrir I, Ein-Dor L, Zuk O, Notterman DA, Domany E. Sorting points into neighborhoods (SPIN): data analysis and visualization by ordering distance matrices. Bioinformatics. 2005;21(10):2301–8. doi: 10.1093/bioinformatics/bti329 15722375

[16] Xie J, Ma A, Zhang Y, Liu B, Cao S, Wang C, et al. QUBIC2: a novel and robust biclustering algorithm for analyses and interpretation of large-scale RNA-Seq data. Bioinformatics. 2020;36(4):1143–9. doi: 10.1093/bioinformatics/btz692 31503285 PMC8215922

[17] Li G, Ma Q, Tang H, Paterson AH, Xu Y. QUBIC: a qualitative biclustering algorithm for analyses of gene expression data. Nucleic Acids Res. 2009;37(15):e101. doi: 10.1093/nar/gkp491 19509312 PMC2731891

[18] Lan C, Tang X, Liu C. A survey of biclustering and clustering methods in clustering different types of single-cell RNA sequencing data. Brief Funct Genomics. 2025;24:elaf010. doi: 10.1093/bfgp/elaf010 40795763 PMC12342763

[19] Shi F, Huang H. Identifying Cell Subpopulations and Their Genetic Drivers from Single-Cell RNA-Seq Data Using a Biclustering Approach. J Comput Biol. 2017;24(7):663–74. doi: 10.1089/cmb.2017.0049 28657835 PMC5510693

[20] Tang X, Liu C, Lan C. scDBic: a novel deep learning-based biclustering algorithm for analyzing scRNA-seq data. Bioinformatics. 2026;42(3):btag095. doi: 10.1093/bioinformatics/btag095 41746287 PMC13012890

[21] Zhao Y, Kohl C, Rosebrock D, Hu Q, Hu Y, Vingron M. CAbiNet: joint clustering and visualization of cells and genes for single-cell transcriptomics. Nucleic Acids Res. 2024;52(13):e57. doi: 10.1093/nar/gkae480 38850160 PMC11260446

[22] Pons P, Latapy M. Computing communities in large networks using random walks (long version). 2005.

[23] van der Maaten L, Hinton G. Visualizing data using t-SNE. Journal of Machine Learning Research. 2008;9(86):2579–605.

[24] McInnes L, Healy J, Melville J. UMAP: Uniform Manifold Approximation and Projection for Dimension Reduction. In: 2020. https://arxiv.org/abs/1802.03426

[25] Chari T, Pachter L. The specious art of single-cell genomics. PLoS Comput Biol. 2023;19(8):e1011288. doi: 10.1371/journal.pcbi.1011288 37590228 PMC10434946

[26] Narayan A, Berger B, Cho H. Assessing single-cell transcriptomic variability through density-preserving data visualization. Nat Biotechnol. 2021;39(6):765–74. doi: 10.1038/s41587-020-00801-7 33462509 PMC8195812

[27] Greenacre M. Correspondence Analysis in Practice. 3rd ed. Chapman & Hall. 2017.

[28] Soneson C. TabulaMurisData: 10x and SmartSeq2 data from the Tabula Muris Consortium. https://bioconductor.org/packages/TabulaMurisData 2024.

[29] Hansen KD, Risso D, Hicks S. TENxPBMCData: PBMC data from 10X Genomics. 2024. https://bioconductor.org/packages/TENxPBMCData

[30] Gralinska E, Kohl C, Sokhandan Fadakar B, Vingron M. Visualizing Cluster-specific Genes from Single-cell Transcriptomics Data Using Association Plots. J Mol Biol. 2022;434(11):167525. doi: 10.1016/j.jmb.2022.167525 35271868

[31] Takahashi A, Ohtani N, Yamakoshi K, Iida S, Tahara H, Nakayama K, et al. Mitogenic signalling and the p16INK4a-Rb pathway cooperate to enforce irreversible cellular senescence. Nat Cell Biol. 2006;8(11):1291–7. doi: 10.1038/ncb1491 17028578

[32] Eymin B, Leduc C, Coll J-L, Brambilla E, Gazzeri S. p14ARF induces G2 arrest and apoptosis independently of p53 leading to regression of tumours established in nude mice. Oncogene. 2003;22(12):1822–35. doi: 10.1038/sj.onc.1206303 12660818

[33] Lun ATL, McCarthy DJ, Marioni JC. A step-by-step workflow for low-level analysis of single-cell RNA-seq data with Bioconductor. F1000Research. 2016;5(2122). doi: 10.12688/f1000research.9501.2 PMC5112579 27909575

[34] W FE. Cluster analysis of multivariate data : efficiency versus interpretability of classifications. Biometrics. 1965;21:768–9.

[35] Herman JS, Sagar GD. FateID infers cell fate bias in multipotent progenitors from single-cell RNA-seq data. Nat Methods. 2018;15(5):379–86. doi: 10.1038/nmeth.4662 29630061

[36] Grün D, Lyubimova A, Kester L, Wiebrands K, Basak O, Sasaki N, et al. Single-cell messenger RNA sequencing reveals rare intestinal cell types. Nature. 2015;525(7568):251–5. doi: 10.1038/nature14966 26287467

[37] Kiselev VY, Kirschner K, Schaub MT, Andrews T, Yiu A, Chandra T, et al. SC3: consensus clustering of single-cell RNA-seq data. Nat Methods. 2017;14(5):483–6. doi: 10.1038/nmeth.4236 28346451 PMC5410170

[38] Fang Q, Su D, Ng W, Feng J. An Effective Biclustering-Based Framework for Identifying Cell Subpopulations From scRNA-seq Data. IEEE/ACM Trans Comput Biol Bioinform. 2021;18(6):2249–60. doi: 10.1109/TCBB.2020.2979717 32167906

[39] Zappia L, Phipson B, Oshlack A. Splatter: simulation of single-cell RNA sequencing data. Genome Biol. 2017;18(1):174. doi: 10.1186/s13059-017-1305-0 28899397 PMC5596896

[40] Zaunders JJ, Lévy Y, Seddiki N. Exploiting differential expression of the IL-7 receptor on memory T cells to modulate immune responses. Cytokine Growth Factor Rev. 2014;25(4):391–401. doi: 10.1016/j.cytogfr.2014.07.012 25130296

[41] Schaum N, Karkanias J, Neff NF, May AP, Quake SR, Wyss-Coray T, et al. Single-Cell Transcriptomics of 20 Mouse Organs Creates a Tabula Muris. Nature. 2018;562(7727):367–372. doi: 10.1038/s41586-018-0590-4 30283141 PMC6642641

[42] Cheng Y, Church GM. Biclustering of Expression Data. In: Proceedings - International Conference on Intelligent Systems for Molecular Biology, 2000. 93–103. 10977070

[43] Lazzeroni L, Owen A. Plaid models for gene expression data. Stat Sin. 2002;12(1):61–86.

[44] Sill M, Kaiser S, Benner A, Kopp-Schneider A. Robust biclustering by sparse singular value decomposition incorporating stability selection. Bioinformatics. 2011;27(15):2089–97. doi: 10.1093/bioinformatics/btr322 21636597

[45] DeBruine ZJ, Melcher K, Triche TJ. Fast and robust non-negative matrix factorization for single-cell experiments. 2021.

[46] Padilha VA, Campello RJGB. A systematic comparative evaluation of biclustering techniques. BMC Bioinformatics. 2017;18(1):55. doi: 10.1186/s12859-017-1487-1 28114903 PMC5259837

[47] Patrikainen A, Meila M. Comparing subspace clusterings. IEEE Trans Knowl Data Eng. 2006;18(7):902–16. doi: 10.1109/tkde.2006.106

[48] Gloub GH, Van Loan CF. Matrix Computations. 3rd ed. Johns Hopkins University Press. 1996.

[49] Bro R, Acar E, Kolda TG. Resolving the sign ambiguity in the singular value decomposition. Journal of Chemometrics. 2008;22(2):135–40. doi: 10.1002/cem.1122

[50] Arthur D, Vassilvitskii S. K-Means: The Advantages of Careful Seeding. In: Proceedings of the Eighteenth Annual ACM-SIAM Symposium on Discrete Algorithms, 2007. 1027–35.

[51] Gralinska E, Vingron M. Association Plots: visualizing cluster-specific associations in high-dimensional correspondence analysis biplots. Journal of the Royal Statistical Society Series C: Applied Statistics. 2023;72(4):1023–40. doi: 10.1093/jrsssc/qlad039

[52] Hu C, Li T, Xu Y, Zhang X, Li F, Bai J, et al. CellMarker 2.0: an updated database of manually curated cell markers in human/mouse and web tools based on scRNA-seq data. Nucleic Acids Res. 2023;51(D1):D870–6. doi: 10.1093/nar/gkac947 36300619 PMC9825416

[53] Kuhn HW. The Hungarian method for the assignment problem. Naval Research Logistics. 1955;2(1–2):83–97. doi: 10.1002/nav.3800020109

[54] Franzén O, Gan L-M, Björkegren JLM. PanglaoDB: a web server for exploration of mouse and human single-cell RNA sequencing data. Database. 2019;2019. doi: 10.1093/database/baz046 PMC6450036 30951143

[55] Subramanian A, Tamayo P, Mootha VK, Mukherjee S, Ebert BL, Gillette MA, et al. Gene set enrichment analysis: a knowledge-based approach for interpreting genome-wide expression profiles. Proc Natl Acad Sci U S A. 2005;102(43):15545–50. doi: 10.1073/pnas.0506580102 16199517 PMC1239896

[56] Korotkevich G, Sukhov V, Budin N, Shpak B, Artyomov MN, Sergushichev A. Fast Gene Set Enrichment Analysis. 2021.

[57] McCarthy DJ, Campbell KR, Lun ATL, Wills QF. Scater: Pre-processing, quality control, normalization and visualization of single-cell RNA-seq data in R. Bioinformatics. 2017;33(8):1179–86. doi: 10.1093/bioinformatics/btw777 28088763 PMC5408845

[58] Rosebrock D, Arora S, Mutukula N, Volkman R, Gralinska E, Balaskas A, et al. Enhanced cortical neural stem cell identity through short SMAD and WNT inhibition in human cerebral organoids facilitates emergence of outer radial glial cells. Nat Cell Biol. 2022;24(6):981–95. doi: 10.1038/s41556-022-00929-5 35697781 PMC9203281

[59] Risso D, Cole M. scRNAseq: Collection of public single-cell RNA-seq datasets. https://bioconductor.org/packages/scRNAseq

[60] Freytag S, Tian L, Lönnstedt I, Ng M, Bahlo M. Comparison of clustering tools in R for medium-sized 10x Genomics single-cell RNA-sequencing data. F1000Res. 2018;7:1297. doi: 10.12688/f1000research.15809.2 30228881 PMC6124389

[61] Tirosh I, Izar B, Prakadan SM, Wadsworth MH 2nd, Treacy D, Trombetta JJ, et al. Dissecting the multicellular ecosystem of metastatic melanoma by single-cell RNA-seq. Science. 2016;352(6282):189–96. doi: 10.1126/science.aad0501 27124452 PMC4944528

[62] Ding J, Adiconis X, Simmons SK, Kowalczyk MS, Hession CC, Marjanovic ND, et al. Systematic comparison of single-cell and single-nucleus RNA-sequencing methods. Nat Biotechnol. 2020;38(6):737–46. doi: 10.1038/s41587-020-0465-8 32341560 PMC7289686

[63] Wang M, Hu Q, Lv T, Wang Y, Lan Q, Xiang R, et al. High-resolution 3D spatiotemporal transcriptomic maps of developing Drosophila embryos and larvae. Dev Cell. 2022;57(10):1271-1283.e4. doi: 10.1016/j.devcel.2022.04.006 35512700

[64] The Tabulasapiens Consortium. The Tabula Sapiens: A multiple-organ, single-cell transcriptomic atlas of humans. Science. 2022;376(6594):eabl4896. doi: 10.1126/science.abl4896 PMC9812260 35549404

[65] Darmanis S, Sloan SA, Zhang Y, Enge M, Caneda C, Shuer LM, et al. A survey of human brain transcriptome diversity at the single cell level. Proc Natl Acad Sci U S A. 2015;112(23):7285–90. doi: 10.1073/pnas.1507125112 26060301 PMC4466750

[66] Baron M, Veres A, Wolock SL, Faust AL, Gaujoux R, Vetere A. A single-cell transcriptomic map of the human and mouse pancreas reveals inter- and intra-cell population structure. Cell Systems. 2016;3(4):346-360.e4. doi: 10.1016/j.cels.2016.08.011 PMC5228327 27667365

[67] Satija R, Satija Lab and Collaborators. Seurat - guided clustering tutorial. https://satijalab.org/seurat/articles/pbmc3k_tutorial 2025.

[68] Hubert L, Arabie P. Journal of Classification. 1985;2(1):193–218. doi: 10.1007/BF01908075

[69] Danon L, Díaz-Guilera A, Duch J, Arenas A. Comparing community structure identification. J Stat Mech. 2005;2005(09):P09008–P09008. doi: 10.1088/1742-5468/2005/09/p09008

[70] Chiquet J, Rigaill G, Sundqvist M. Aricode: Efficient computations of standard clustering comparison measures. 2018.

