# Supplementary material for: CAdir: Joint clustering of cells and genes for single-cell transcriptomics with visualization-driven cluster quality assessment
Source: PLoS Comput Biol. 2026 Jun 30;22(6):e1014418. doi: 10.1371/journal.pcbi.1014418 (PMC13349309; doi:10.1371/journal.pcbi.1014418)
Supplement: S1 Appendix — Additional comparisons, analyses and benchmarkings that give additional insights into the performance and functionality of CAdir. (PDF) [file pcbi.1014418.s001.pdf]

Supporting Information - S1 Appendix
CAdir: Joint clustering of cells and genes for single-cell transcriptomics with visualization-driven cluster quality assessment

Clemens Kohl[1] and Martin Vingron[1,*]

[1] Department of Computational Molecular Biology, Max Planck Institute for Molecular Genetics, Berlin, Germany
[*] Corresponding author. Email: vingron@molgen.mpg.de

Supplementary Results

Runtime Comparison

Unlike other clustering algorithms, such as SIMLR, Monocle3 or RaceID, which show a significant drop in performance for larger data sets in comparison to the top performing algorithms such as CAbiNet (Fig 5C and 5D), CAdir provides highly robust clustering results for both small and large data sets. This feature of CAdir is of particular interest due to the continuously increasing size of single-cell RNA-seq data.

Not all clustering algorithms are able to capitalize on their clustering performance when applied to very large data sets such as e.g. tissue atlas data sets, due to long runtimes. CAdir is highly scalable (S7A Fig) and can cluster a data set with 600 000 cells in approximately 30 min. In our comparison, CAdir is the fastest algorithm tested, even surpassing k-means and Seurat. In particular, SIMLR is unable to cluster any data set with more than 1000 cells.

Although SC3 has even shorter runtimes, it achieves this by only clustering a small subset of 5000 cells and uses them to train a Support Vector Machine (SVM) to assign the cluster labels to the remaining cells [1]. Due to the small number of cells sampled, this approach however does not adequately represent the diversity present in large scRNA-seq data sets. This is also evident from the poor clustering results for the moderately large Brain Organoids and Tabula Sapiens data sets reported in Fig 5C and 5D.

Validation of Marker Genes

The co-clustered genes provided by CAdir can help in annotating the cells and can improve downstream analysis. For this reason, we validated the co-clustered genes for the PBMC3k data set based on known marker genes that are cell type specific. S3A, S3B, S3C, S3D and S3E Fig show the proportion of co-clustered genes with an S_θ-score of more than 0 that are also contained in the CellMarker [2] gene set used to annotate the cluster. For the majority of clusters (3 out of 5) more than 50 % of the co-clustered marker genes are part of the CellMarker gene set (S3A, S3B and S3D Fig), and for another (Monocytes) 48 % of the genes are contained in the gene set. As already discussed in the Results in the main text, the Megakaryocytes cluster is also comprised of Platelet cells, which share most of their RNA with Megakaryocytes. The only cluster that does not have any cell type specific marker genes is cluster 6 (S3F Fig), which

also could not be annotated by CAdir's automatic cell type annotation. However, from the ground truth annotation (S3G Fig) it is clear that the cluster consists of CD4+ T cells. Looking at the proportion of co-clustered genes in cluster 6, none are contained in the CD4+ T cells gene set, explaining why it could not be annotated. One possible explanation for this is the fact that CD8+ T cells share many of the same marker genes, which due to its smaller size exhibits smaller variation in its expression patterns, making marker gene assignment easier. However, because CAdir uniquely assigns genes to a single cluster, no T cell marker genes remain for cluster 6.

The top 3 genes with the highest S_θ-score for each cluster are shown in Table B. Looking more closely at the co-clustered genes for the B cell cluster, the highest ranked gene is *CD79A*, also known as "B-Cell Antigen Receptor Complex-Associated Protein Alpha Chain", which is exclusively expressed by B cells. Ranked second is *HLA-DOB*, which is expressed by antigen presenting cells such as B cells. Similarly, *ASGR1*, Asialoglycoprotein receptor 1, the top ranked gene for the Monocyte cluster, is well known to be expressed in peripheral blood monocytes, although its exact function is still being studied [3]. The gene ranked second, *FCGR1A*, is again expressed by antigen presenting cells and is primarily expressed in the monocyte lineage. Overall, this demonstrates that the co-clustered genes by CAdir are biologically meaningful.

Comparison to Scran Marker Gene Detection

In order not to rely on the previously used intentionally poor clustering of the PBMC3k data, we re-clustered the PBMC3k data for a better clustering result (see Section Discussed Data for details). Because we want to compare the overall quality of the co-clustered genes, we decreased the quantile cutoff for assigning genes to 0.3, which filters out only the 30 % least specific genes before assigning the remainder to clusters. This results in 7 clusters, which are annotated by CAdir as Naive CD4+ T cell, Monocyte, Dendritic cell, CD8+ T cell, Cytotoxic CD4+ T cell, B cell and Megakaryocyte and achieves an overall ARI of 0.63 for the cell clustering. Table E shows the resulting number of cells and genes for each bicluster.

Compared to other methods for marker gene detection such as scran's `scoreMarkers` [4], which typically perform pairwise statistical tests between clusters, CAdir assigns genes to a cluster if they are highly expressed only in a single cluster. Therefore, co-clustered genes are typically only expressed in that cluster and can be used to differentiate between the different cell types in the data. S4 Fig shows the 4 genes with the highest S_θ-score for the B cell, Monocyte and Cytotoxic CD4+ T cell clusters and compares it to the 4 genes with the highest mean Area Under the Curve (AUC) over all pairwise comparison as obtained from scran's `scoreMarkers`. For the purpose of this comparison, the AUC can be interpreted as the probability that the expression of a gene in a randomly sampled cell from a cluster is higher than in cells outside the cluster. For the B cells (S4A and S4B Fig) both scran and CAdir highly rank genes that are almost uniquely expressed in the B cell cluster. In fact, both *CD79A* and *MS4A1* appear are among the top 4 genes for both methods. However, unlike CAdir, scran also includes *CD37* which is expressed in other cell types too, albeit at a lower level. This can also be observed for the Monocyte cluster (S4C and S4D Fig), where CAdir's top 4 genes are only expressed in the Monocyte cluster, whereas scran's 4 highest ranked genes are also expressed in other cell types at lower levels. While CAdir is therefore better in identifying genes that are unique to a cluster, scran's `scoreMarkers` can be used to identify small, but consistent changes in gene expression between clusters.

CAdir's co-clustered genes can also be used to differentiate between related cell types. For example, for the Cytotoxic CD4+ T cells scran identifies genes that are also substantially expressed in the CD8+ T cells, such as *NKG7* or *CTSW* (S4F Fig). CAdir on the other hand only co-clusters genes that are not substantially expressed in the CD8+ T cells (S4E Fig). Both

approaches have their advantages and disadvantages, depending on the goals of the analysis. It can therefore be beneficial to perform both co-clustering with CAdir and to detect marker genes with scran to get a comprehensive picture of the data.

We further compared the overall overlap of genes found between scran's `scoreMarkers` and CAdir's co-clustered genes. To this end, we performed pairwise comparisons using `scoreMarkers` on the cell clusters obtained by CAdir. Because ranking the genes by the mean AUC does not provide a clear cutoff, we selected the same number of top ranked genes as the number of genes co-clustered by CAdir for a specific cluster, as long as the mean AUC was above 0.5. S5A Fig shows the overlap of genes found by both methods. For most clusters the overlap ranges from approximately 40-60 % with the highest overlap being 68 % for the Megakaryocyte cluster, but for the CD8+ T cells it only reaches 23 %. When selecting only those genes that have an S_θ-score > 0 (see S5B Fig), and therefore reducing the overall number of genes in the comparison, the overlap increases for the CD8+ T cells to 38 %, but is reduced for others, such as Cytotoxic CD4+ T cells (35 %) and Naive CD4+ T cells (11 %), indicating that the genes more highly ranked for these clusters by scran are not exclusive to the cluster.

Selecting Cluster Specific Genes

CAdir assigns the genes with the shortest orthogonal distance to a cluster direction to each cluster. While this process can in principle assign each gene to some cluster, this assignment is meaningful only for those genes that actually show a certain degree of specificity. Therefore, by default the program filters out 80 % of the co-clustered genes that are closest to the origin, based on the vector norm, thereby only providing genes that are likely to be truly specific for a cluster. Downstream analyses such as cell type annotation often do not profit from reporting a too extensive list of co-clustered genes and filtering removes genes that are more likely to be less indicative of a cell type. Naturally, the number of genes reported using the 80 % quantile cutoff depend on the number of genes provided to the clustering algorithm. In our experience, approximately 100-500 genes per cluster are typically more than sufficient to identify a cell type, and adding more cluster unspecific genes is not beneficial. Because users might want to perform different downstream analyses or perform clustering on data sets with substantially more or less genes, CAdir allows changing the cutoff to better suit the needs of the user.

Importantly, changing the cutoff does not change the clustering results, but only the number of reported cluster specific genes, and different cutoffs can therefore be explored for the same clustering results. For exploratory analysis, the cutoff can be set to 0, thereby keeping all genes, and the genes can subsequently be ranked for specificity by their S_θ-score. Filtering based on the distance to the origin is unbiased with respect to the clustering, whereas the S_θ-score helps to understand which genes are most interesting for a specific cluster after assignment.

To demonstrate how the cluster specificity changes with decreasing vector norm and S_θ-score, we show two examples in S14 Fig. Because the length of the gene vectors is preserved in the Association Plot [5], we can observe the change in cluster specificity by looking at the gene expression at different distances from the origin. Typical B cell marker genes such as *Cd19* (S_θ-score: 2.66, vector norm: 4.38) and *Cd79a* (S_θ-score: 2.48, vector norm: 3.81) have high S_θ-scores and, as shown by the violin plots, are exclusively expressed in the B cell cluster (S14A Fig). *Ly6d* (S_θ-score: 1.02, vector norm: 3.09) is already expressed at a low level in other clusters, but can still be regarded as specific for the B cell cluster. However, closer to the origin the genes are increasingly unspecific. *Cd74* (S_θ-score: 1.02, vector norm: 2.32) already shows high expression in the Macrophage cluster besides the B cell cluster, and *Bmf* (S_θ-score: -1.05, vector norm: 1.65), with a negative S_θ-score, is not specific for any cluster (S14A Fig).

For the Endothelial cell cluster, *Cdh5* (S_θ-score: 2.78, vector norm: 3.1) and *Pecam1* (S_θ-

score: 1.89, vector norm: 2.57) are expressed in virtually all cells of the Endothelial cell cluster, making them excellent marker genes (S14B Fig). To a lesser degree, the two genes are also expressed in a small amount in the Vascular endothelial cell cluster, which is unsurprising as they occupy a similar direction (see main text Fig 3C). *Kitl* (S_θ-score: 1.36, vector norm: 2.28) also shows a high expression in the Endothelial cluster, but the expression in the other clusters is also increased by a small amount compared to the two higher ranked genes. Going even closer to the origin, the co-clustered genes are already expressed to a higher degree in other clusters. *Itpkb* (S_θ-score: 0.02, vector norm: 1.89), although expressed in the Endothelial cell cluster, is also meaningfully expressed in the T cell cluster, but the overall expression is low. Lastly, *Dlg2* (S_θ-score: -1.56, vector norm: 1.73) is not meaningfully expressed in any of the clusters (S14B Fig).

The two discussed example clusters demonstrate, that the cluster specificity of a gene indeed improves with increasing distance to the origin. In the above examples, only genes larger than the 80 % quantile cutoff were assigned to a cluster, which corresponds to a vector norm of 1.63. As the two genes close to the threshold, *Bmf* and *Dlg2*, show, genes on the lower end of this cutoff already tend not to be cluster specific and therefore an even more stringent cutoff could theoretically be applied.

Estimating the Number of Clusters

We furthermore tested the ability of CAdir to correctly identify the number of clusters in the data. Similarly to Yu et al. [6], we sampled increasing amounts of cell type clusters from the Tabula Muris cell atlas [7] and tested if CAdir is able to identify the correct number of clusters when estimating the cutoff angle by itself. Up to approx. 20 clusters CAdir provides cluster estimations very close to the ground truth (S7B Fig). While CAdir is able to recover the correct amount of clusters at the beginning, the deviation from the sampled number of clusters grows with an increasing number of cell types.

Notably, the Tabula Muris data consists of cells from 18 different tissues, many of which contain similar cell types. This leads CAdir to primarily cluster by tissue instead of by cell type, explaining why it performs best around 15-20 sampled clusters.

We additionally explored the effect of the initial k on the final results. Testing 6 different choices of k to initialize CAdir, the differences between the runs are marginal (S7B Fig). This further confirms the ability of CAdir to determine the cluster number largely independent of the initialization. Similarly, the choice of the cutoff quantile used to automatically determine the angle for splits and merges seems to only have a minor effect (S7B Fig). Generally, a more restrictive quantile cutoff, which results in a lower cutoff angle, appears to slightly improve the recovery of the correct number of clusters.

We similarly tested the number of clusters recovered using Seurat. To this end, we evaluated it using different numbers of nearest neighbors (NNs) for the SNN graph and also evaluated three settings for the resolution parameter when performing Leiden clustering. As shown in S15 Fig, Seurat generally overestimates the number of clusters, in particular if a lower number of nearest neighbors is chosen for the SNN graph. Interestingly, the difference between the number sampled and recovered clusters shrinks with increasing number of cell types sampled. Overall, CAdir seems to perform slightly better when less cell types are sampled, whereas Seurat has a clear advantage for data sets with many cell types.

Effect of the Initialization Method

We tested the effect of the two implemented initialization methods for determining the starting directions of CAdir, namely kmeans$_{++}$ and random initialization, on the final clustering results using the Tabula Muris Limb Muscle data. Since the number of initial directions might affect the performance of the initialization method, we initialized CAdir with the number of known ground truth clusters ($k = 8$), as well as a sub-optimal cluster number ($k = 5$). Each clustering was repeated for 100 times to obtain robust results and evaluated by the ARI of the cell clustering. As can be seen in S18 Fig, both kmeans$_{++}$ (`kmeanspp`) and random (`rand`) initialization obtain almost identical results, independent of whether 5 or 8 clusters are requested.

Benchmarking on Simulated Data with Increased Sparsity

To better test the cell clustering and biclustering performance of CAdir on sparser data, we increased the number of dropouts in the simulated data. Using Splatter, the probability of a given gene to be a dropout is governed by a logistic function. To better reflect the nature of dropouts in scRNA-seq data, the probability for a gene to be dropped depends on the gene expression. Lowly expressed genes are consequently assigned a higher probability to be a dropout compared to higher expressed genes. In order to increase the number of 0 values and therefore increase sparsity in the simulated data, we decreased the parameter `dropout.mid`, which sets the expression at which the probability is 0.5, to 20 and decreased the fall-of `dropout.shape` to -0.05. A comparison of the new logistic function can be seen in S16A Fig and the dropout rates estimated by Splatter for both the Zeisel and PBMC3k data sets are shown in S16B and S16C Fig. As can be seen, the new logistic function not only assigns a higher probability to be a dropout to lowly expressed genes, but also increases the probability for highly expressed genes to be dropped. Depending on the chosen simulation parameters, this does not affect each simulated data set to the same degree. On average the sparsity for simulated data based on the Zeisel data set is increased from 84% zeros to 93% zeroes, whereas the percentage of zeroes is increased from 95% to 97% for PBMC3k based data due to its already high sparsity.

Tunable Parameters

An overview over the 8 tunable parameter for CAdir and their default values is given in Table A. As discussed in more detail in Supplementary Section ”Effect of Parameter Choices”, most parameters do not require tuning in the majority of cases. For example, the parameters `max_n` and `max_r` typically do not have to be changed, as the maximum number of iterations is only rarely reached because convergence is achieved earlier. Instead, they can be set if only very limited computational resources are available or if working on extremely large data sets. Similarly, the cutoff angle can be automatically inferred by CAdir, and therefore typically can be left on the default value. Depending on how well cells and genes from different clusters should be separated, the parameters for the APL quantile cutoff can be changed. As shown in Supplementary Results Section ”Number of Random Directions For Cutoff Inference”, if more than 100 random directions are generated to calculate the cutoff angle, a further increase would only lead to extremely minor changes in the inferred angle.

Effect of Parameter Choices

To better demonstrate how the selection of these parameters affects the clustering outcome we performed benchmarkings for the following parameters: Dimensionality, number of iterations,

Association Plot quantile cutoff and the number of random directions for the cutoff angle inference. In order to isolate the effect of a single parameter, for each data set the best performing parameters obtained from the cell clustering benchmarking were used, the initial random directions re-used between iterations for the same data set and only the parameter in question was varied.

Choice of Dimensionality

The APL package [8], on which we build to perform correspondence analysis, offers a number of methods to determine the number of dimensions to retain automatically. One option is to determine the elbow of the scree plot either manually or by performing repeated CA of the permuted data as described in Gralinska et al. [5]. Besides the elbow method, the APL package can determine the number of dimensions that cumulatively explain 80 % of the inertia and provides a scree plot that allows for manual selection of the number of dimensions based on the elbow in the plot.

To test the effect of the chosen number of CA dimensions on the final clustering results, we clustered the experimental data sets using between 5 and 150 dimensions in steps of 5 dimensions. As shown in S11A Fig, for most data sets, namely the Tabula Sapiens, Brain Organoids, Freytag Gold, Dmel Spatial and PBMC10x data sets, the ARI quickly improves over the first 30 dimensions and then plateaus. Other data sets exhibit more complicated behaviors: The Tirosh data set for example is slowly increasing while simultaneously showing a stronger variance over the number of dimensions, whereas the Zeisel data set exhibits a spike in clustering performance at 10 dimensions. The performance on the Baron Pancreas data set on the other hand first decreases and then quickly increases until reaching a temporary plateau after 40 dimensions. Lastly, the clustering performance on the Darmanis data set is largely independent on the chosen dimensionality (S11A Fig).

This shows that although most data sets seem to profit from choosing more than 30 dimensions, the dimensionality should be chosen based on a per data set basis, using for example the APL package's elbow method or a scree plot. As a starting point for an analysis, all tested data sets seem to perform well between 30-50 dimensions. Although a careful choice of the number of dimensions can improve the clustering performance, the difference between the highest and the lowest achieved ARI is smaller than 0.2 for all data sets if more than 30 dimensions are chosen. In fact, on average the maximum difference is only approximately 0.09. This shows, that despite the influence of the dimensionality on the clustering result, CAdir can robustly achieve good clustering results.

Choice of Angle Quantile Cutoff

For most clustering tasks, a 0.99 quantile cutoff is sufficiently specific, which is also supported by the results on the discussed experimental data sets and benchmarking results. The cell clustering benchmarking already included three different quantiles to determine the cutoff angle, 0.99, 0.999 and 0.9999. To understand how CAdir's clustering performance depends on the chosen quantile cutoff, we additionally tested a wide range of quantile cutoffs. Namely, we benchmarked the cell clustering of CAdir on the experimental data sets using an Association Plot (APL) cutoff quantile ranging from 0.7 to 0.9999. As expected, in general the clustering fidelity improves up to the 0.99 quantile (S11B Fig). For the Zeisel data set, the ARI increases from 0.51 for the 0.78 quantile to 0.82 for the 0.98 quantile. Similarly, the Baron Pancreas data set increases from 0.37 for the 0.7 quantile to 0.95 for the 0.99 quantile (S11B Fig). Other data sets do not show quite so strong differences, and in some cases, e.g. the Freytag Gold data set, the quantile cutoff does not seem to influence the clustering result at all, likely due to the fact that

the cell types are easily identifiable in the data set. Interestingly, if a quantile higher than 0.99 is chosen (0.999 or 0.9999), the clustering quality drops for some data sets (Baron Pancreas, Zeisel, Darmanis) but increases for others (Brain Organoids, Tirosh) (S11B Fig). Overall, a quantile cutoff between 0.95-0.99 seems to yield the best clustering results. CAdir therefore uses the 0.99 quantile as the default cutoff.

Cutoff Angle Inference Methods

To more rigorously test the difference between the angle inferred using Association Plots for random directions and permuted data (see also Section ”Automatic Cutoff Angle Inference” in the Methods), we tested the result of both methods on all experimental data sets used in the benchmarking. S19A Fig shows the difference between the angle inferred using the permuted data (”perm”) and by randomly choosing directions (”rand”). For the majority of cases the difference between the two methods is minimal. On average, the permutation method infers an angle 0.2 ° higher than the method using random direction. For the data set with the largest difference, the Baron Pancreas data set, the difference is still only 1.2 °. Consequently, the effect on the clustering results is negligible, as shown in S19B Fig. By default, CAdir therefore uses the faster random directions to estimate the cutoff angle.

Number of Random Directions For Cutoff Inference

Although determining the cutoff angle through Association Plots in random directions is comparatively computationally cheap, we tested how many random directions are required to obtain a robust estimate of the cutoff angle. To this end, we determined the cutoff angle using between 1 and 1000 random directions in intervals of 10 on the experimental data sets. For the majority of data sets the cutoff angle stabilizes quickly within the first 50-100 directions (S12A Fig). Contrary to the other data sets, the Zeisel data set exhibits a larger amount of variance and only stabilizes after approximately 250 directions. For most data sets, 100 random directions should therefore be sufficient to obtain a good estimate of the cutoff angle. As shown in S12B Fig, the cell clustering does not improve if more than 100 directions are used and only minor fluctuations around a stable value can be observed. CAdir's default value is therefore set to 100 random directions.

Number of Iterations

CAdir iterates until the angle of the found cluster directions to the matching directions from the previous iteration is smaller than a cutoff, by default 0.001 °, up to a maximum of 50 iterations. From our observations, the cutoff is typically reached within the first few iterations. To test the influence of the number of iterations on the clustering results, we tested between 1 to 50 split and merge iterations for CAdir. As shown in S13A and S13B Fig for experimental and simulated data respectively, the cell clustering performance, as measured by the ARI, stabilizes quickly within the first 5 iterations for virtually all data sets. Only for a single outlier on simulated data, the most difficult simulated data set, the ARI stabilizes only after 20 iterations. We also tested how fast the DIRCLUST sub-step of CAdir (Algorithm 1 in the main text) converges to a stable clustering result. As can be seen in S13C and S13D Fig, for both experimental and simulated data the clustering converges to a stable result within 10 iterations at most. CAdir checks during each iteration whether the angle between the new cluster directions to the directions from the previous iteration falls below the convergence threshold and as a result it only performs as many

iterations as strictly necessary. The quick convergence to a stable clustering results underlines the robust nature of CAdir.

Overview over Software Functionality

CAdir is designed to be adjustable and provide flexible visualizations that fit the research questions. For clustering, CAdir provides two separate functions: `dirclust`, which clusters the data into a predefined number of clusters, and `dirclust_splitmerge`, which dynamically adjusts the number of clusters based on the angle between them. If desired, the functions offer fine-grained control over the hyperparameters: The parameter `k` controls the number of initial directions and `cutoff` the cutoff angle for splitting and merging clusters. If set to `NULL` CAdir will infer the angle independently. Similarly, the quantile cutoff for clustering the genes can be controlled via `qcutoff`, which by default is set to 0.8, therefore only considering the 80 % most highly associated genes and discarding the 20 % least specific ones.

A quick overview of the clustering results is provided by `plot_clusters`, which outputs an Association Plot for each cluster found by CAdir. Association Plots for individual clusters can be obtained by `cluster_apl`, which uses the same parameters to control the plotting. The Association Plots are highly adaptable, for example the size of the cell and gene points can be individually controlled using the `point_size` and `size_factor` parameters (S20C and S20D Fig). By default, the Association Plots only highlight the cells and genes of the cluster of interest, but cells and genes can be colored by their respective cluster by setting `highlight_cluster` to `FALSE` (S20A Fig).

If desired, only the cells (S20A Fig) or only the genes (S20B Fig) can be displayed using the parameters `show_cells` and `show_genes`. If only cells or only genes are displayed, the principal coordinates are used. When showing both cells and genes simultaneously, the cluster direction and cell coordinates are converted to standard coordinates for better interpretability (see Section "Scaling of coordinates"). Additionally, the directions of other clusters can be projected into the Association Plot by specifying the clusters of interest using the `show_lines` parameter (S20A Fig). By default, CAdir will not display any gene labels to provide a cleaner overview. Turning `label_genes` to `TRUE` will label the top 10 most highly associated genes, but the number can be changed using the `ntop` parameter (S20B Fig). Additionally, a line with the same angle to the cluster direction as the cutoff angle θ can be plotted in the Association Plot (S20E and S20F Fig). This can help to better understand whether clusters are well separated and which genes are the most cluster specific. Cells and genes that fall to the left and below the line are considered associated to the cluster, as also measured by the S_θ-score. To allow for easier exploration of the Association Plots, an interactive plot can be generated by setting `interactive = TRUE` in `cluster_apl`.

CAdir can also record an Association Plot for each split and merge it performed when `make_plots = TRUE` in `dirclust_splitmerge`, which can then later be retrieved from the `plots` slot in the `cadir` object. A better overview of the splits and merges can be obtained using the split-merge graph. The function `sm_plot` plots a graph showing all splits and merges as well as a small Association Plot for the cluster during this iteration. An example for the split-merge graph plot can be found in Fig 3D.

To annotate the obtained biclusters, the function `annotate_biclusters` can be used. Either "CellMarker" or "PanglaoDB" gene sets can be used for annotation through the `set` parameter. Additionally, the cell type annotation can be performed with either gene set overrepresentation ("goa") or gene set enrichment analysis ("gsea") by setting the `method` parameter accordingly.

References

[1] Vladimir Yu Kiselev, Kristina Kirschner, Michael T. Schaub, Tallulah Andrews, et al. “SC3: Consensus Clustering of Single-Cell RNA-seq Data”. In: *Nature Methods* 14.5 (May 2017), pp. 483–486. DOI: 10.1038/nmeth.4236.

[2] Congxue Hu, Tengyue Li, Yingqi Xu, Xinxin Zhang, et al. “CellMarker 2.0: An Updated Database of Manually Curated Cell Markers in Human/Mouse and Web Tools Based on scRNA-seq Data”. In: *Nucleic Acids Research* 51.D1 (Jan. 2023), pp. D870–D876. DOI: 10.1093/nar/gkac947.

[3] Rebecca Louise Harris, Carmen Wilma van den Berg, and Derrick John Bowen. “ASGR1 and ASGR2, the Genes That Encode the Asialoglycoprotein Receptor (Ashwell Receptor), Are Expressed in Peripheral Blood Monocytes and Show Interindividual Differences in Transcript Profile”. In: *Molecular Biology International* 2012 (2012), p. 283974. DOI: 10.1155/2012/283974. PMID: 22919488.

[4] Aaron T. L. Lun, Davis J. McCarthy, and John C. Marioni. “A Step-by-Step Workflow for Low-Level Analysis of Single-Cell RNA-seq Data with Bioconductor”. In: (Oct. 2016). DOI: 10.12688/f1000research.9501.2.

[5] Elzbieta Gralinska and Martin Vingron. “Association Plots: Visualizing Cluster-Specific Associations in High-Dimensional Correspondence Analysis Biplots”. In: *Journal of the Royal Statistical Society Series C: Applied Statistics* 72.4 (Aug. 2023), pp. 1023–1040. DOI: 10.1093/jrsssc/qlad039.

[6] Lijia Yu, Yue Cao, Jean Y. H. Yang, and Pengyi Yang. “Benchmarking Clustering Algorithms on Estimating the Number of Cell Types from Single-Cell RNA-sequencing Data”. In: *Genome Biology* 23.1 (Feb. 2022), p. 49. DOI: 10.1186/s13059-022-02622-0.

[7] Nicholas Schaum, Jim Karkanias, Norma F. Neff, Andrew P. May, et al. “Single-Cell Transcriptomics of 20 Mouse Organs Creates a Tabula Muris”. In: *Nature* 562.7727 (Oct. 2018), pp. 367–372. DOI: 10.1038/s41586-018-0590-4.

[8] Elzbieta Gralinska, Clemens Kohl, Bita Sokhandan Fadakar, and Martin Vingron. “Visualizing Cluster-specific Genes from Single-cell Transcriptomics Data Using Association Plots”. In: *Journal of Molecular Biology* (Mar. 2022), p. 167525. DOI: 10.1016/j.jmb.2022.167525.

Supplementary Tables

Parameter	Description	Default Value
initial k	Number of initial directions	requires user input
cutoff angle	Cutoff angle for splitting and merging. Angle can be inferred automatically	`NULL` (automatic inference)
convergence thr.	Diff. in angle with directions from prev. iterations	0.001 °
max. n	Maximum number of split/merge iterations	50
max. r	Maximum number of DIRCLUST iterations	50 (initial clustering)
APL quantile	Quantile to infer cutoff angle	0.99
APL repetitions	Number of random association plots for cutoff inference	100
gene quantile	Quantile determining the vector norm cutoff	0.8

Table A: Overview over CADIR's tunable parameters.

Cluster	Gene	S_θ
CD8+_T_cell	LAG3	0.91
CD8+_T_cell	GZMK	0.66
CD8+_T_cell	JAKMIP1	0.43
B_cell	CD79A	1.96
B_cell	HLA-DOB	1.86
B_cell	LINC00926	1.82
Megakaryocyte	GP9	9.78
Megakaryocyte	ITGA2B	9.42
Megakaryocyte	GNG11	9.29
Monocyte	ASGR1	0.97
Monocyte	FCGR1A	0.90
Monocyte	CD14	0.83
Natural_killer_cell	AKR1C3	1.90
Natural_killer_cell	GZMB	1.87
Natural_killer_cell	PRSS23	1.85

Table B: Top 3 marker genes for each PBMC3k cluster with $S_\theta > 0$.

Cell clustering		
**Method**	**Parameter**	**Values**
CAdir	dimensions<br>cutoff angle<br>APL quantile<br>initial k<br>gene quantile	10, 30, 60<br>40, 50, 60<br>0.99<br>5, 10, 15, 20<br>0.8
CAdir_auto	dimensions<br>APL quantile<br>initial k<br>gene quantile	10, 30, 60<br>0.99, 0.999, 0.9999<br>5, 10, 15, 20<br>0.8
CAbiNet_igraph	dimensions<br>k NNs<br>overlap<br>graph select<br>SNN type<br>calc. cell-gene graph	40, 80, 150<br>30, 60, 100<br>0.1<br>TRUE, FALSE<br>all<br>TRUE, FALSE
Seurat	resolution<br>dimensions<br>k NNs<br>alpha	1, 1.5<br>10, 30, 60<br>10, 20, 40, 60, 80, 100<br>0.01, 0.05
Monocle3	resolution<br>dimensions<br>k NNs<br>dim-red. method	0.1, 1<br>20, 30, 60<br>20, 40, 80<br>UMAP, PCA
SIMLR	k<br>dimensions<br>tuning k	3, 5, 6, 8, 10, 15<br>10, 20, 30<br>10 30
SC3	k<br>gene filtering<br>min. # eigenvectors<br>max. # eigenvectors	0, 4, 6, 8, 10, 15<br>TRUE, FALSE<br>0.04, 0.05, 0.06<br>0.07, 0.08, 0.09
RaceID	k<br>metric<br>clustering alg.	5, 8, 10, 15<br>euclidean, pearson, logpearson<br>kmedoids, kmeans, hclust
RaceID_auto	cell samples<br>metric<br>clustering alg.	1000, 2000, 3000, 4000<br>euclidean, pearson, logpearson<br>kmedoids, kmeans, hclust
k-means	k<br>dimensions<br>quantile genes	3, 6, 9, 12, 15, 18<br>10, 20, 30, 40, 50, 60<br>0.8
DivBiclust	difference threshold<br>seed size<br>max gene set size<br>fraction missing<br>similarity threshold	0.15, 0.2, 0.25<br>30, 60, 90<br>100, 200<br>0, 0.1<br>0.5
scDeepCluster	# clusters<br># NNs<br>resolution	4, 6, 8, 10, 12, 14<br>20, 50<br>0.8, 1, 1.2
scG-cluster	# clusters<br>graph distance method<br>pretraining epochs<br>training epochs	4, 6, 8, 10, 12, 14<br>ncos, p, heat<br>400, 800<br>300

Table C: Cell clustering benchmarking - Parameter combinations. The table lists the parameter combinations for each algorithm in the cell clustering benchmarking. Parameters that are not manually set, but instead left on the default value, are not listed. When picking a specific method for, e.g. distance calculation, the names used by the algorithm are listed. For parameters for which the parameter can not be sufficiently described in the available space, the parameter name as used by the algorithm is listed.

Biclustering		
**Method**	**Parameter**	**Values**
CAdir	dimensions	10, 30, 60
	cutoff angle	40, 50, 60
	APL quantile	0.99
	initial k	10, 15
	gene quantile	0.7, 0.8
CAdir_auto	dimensions	10, 30, 60
	APL quantile	0.99, 0.999
	initial k	10, 15, 20
	gene quantile	0.7, 0.8
CAbiNet_igraph	dimensions	40, 80, 150
	k NNs	30, 60, 100
	overlap	0.1
	graph select	TRUE, FALSE
	SNN type	all
	calc. cell-gene graph	TRUE, FALSE
Seurat	resolution	1
	dimensions	10, 30, 60
	k NNs	20, 40, 60
	min %	0.15, 0.25
	logfc threshold	0.25
	alpha	0.01, 0.05
Monocle3	resolution	0.1, 1
	dimensions	20, 30, 60
	k NNs	20, 40, 80
	dim-red. method	UMAP, PCA
	# genes/group	50
BackSPIN	# levels	3, 4, 5, 6
	Stop constant	0.5, 0.825, 1.15
	lower threshold	0.1, 0.2, 0.3
CCA	delta	0.5, 1.0, 1.5, 2.0, 2.5, 3.0
	alpha	0.5, 1.0, 1.5, 2.0, 2.5, 3.0
Plaid	row release	0.50, 0.54, 0.58, 0.62, 0.66, 0.70
	column release	0.50, 0.54, 0.58, 0.62, 0.66, 0.70
QUBIC	r	1, 3, 6
	c	0.9, 0.95, 0.99
	q	0.01, 0.06, 0.1, 0.2
QUBIC2	quantile threshold	0.02, 0.06, 0.1
	nr. clusters	4, 6, 10
	obj. function	KL dual, qubic1 dual
	consistency	1.0, 0.75
s4vd	pcerv	0.01, 0.05, 0.15
	pceru	0.01, 0.05, 0.15
	cutoff threshold min	0.6, 0.7
	cutoff threshold max	0.05, 0.15
NMF	k (rank)	5, 6, 7
	L1 penalty	0, 0.001, 0.01, 0.1
	# restarts	10, 25, 50

Table D: Biclustering benchmarking - Parameter combinations. For each algorithm the 36 parameter combinations used in the biclustering benchmarking are listed. Parameters that are left on the default values are excluded. In order to provide are more meaningful description, where possible a short description of the parameter is given instead of the parameter name. In cases where this is not possible, the parameter name as used by the algorithm is used.

Bicluster	Cells	Genes
**Megakaryocyte**	19	65
**B cell**	347	125
**Naive CD4+ T cell**	1088	122
**Dendritic cell**	41	74
**CD8+ T cell**	291	86
**Cytotoxic CD4+ T cell**	221	103
**Monocyte**	631	125

Table E: CAdir clustering results for the improved PBMC3k clustering.
