# Supplementary figures and images for: CAdir: Joint clustering of cells and genes for single-cell transcriptomics with visualization-driven cluster quality assessment
Source: PLoS Comput Biol. 2026 Jun 30;22(6):e1014418. doi: 10.1371/journal.pcbi.1014418 (PMC13349309; doi:10.1371/journal.pcbi.1014418)

**A**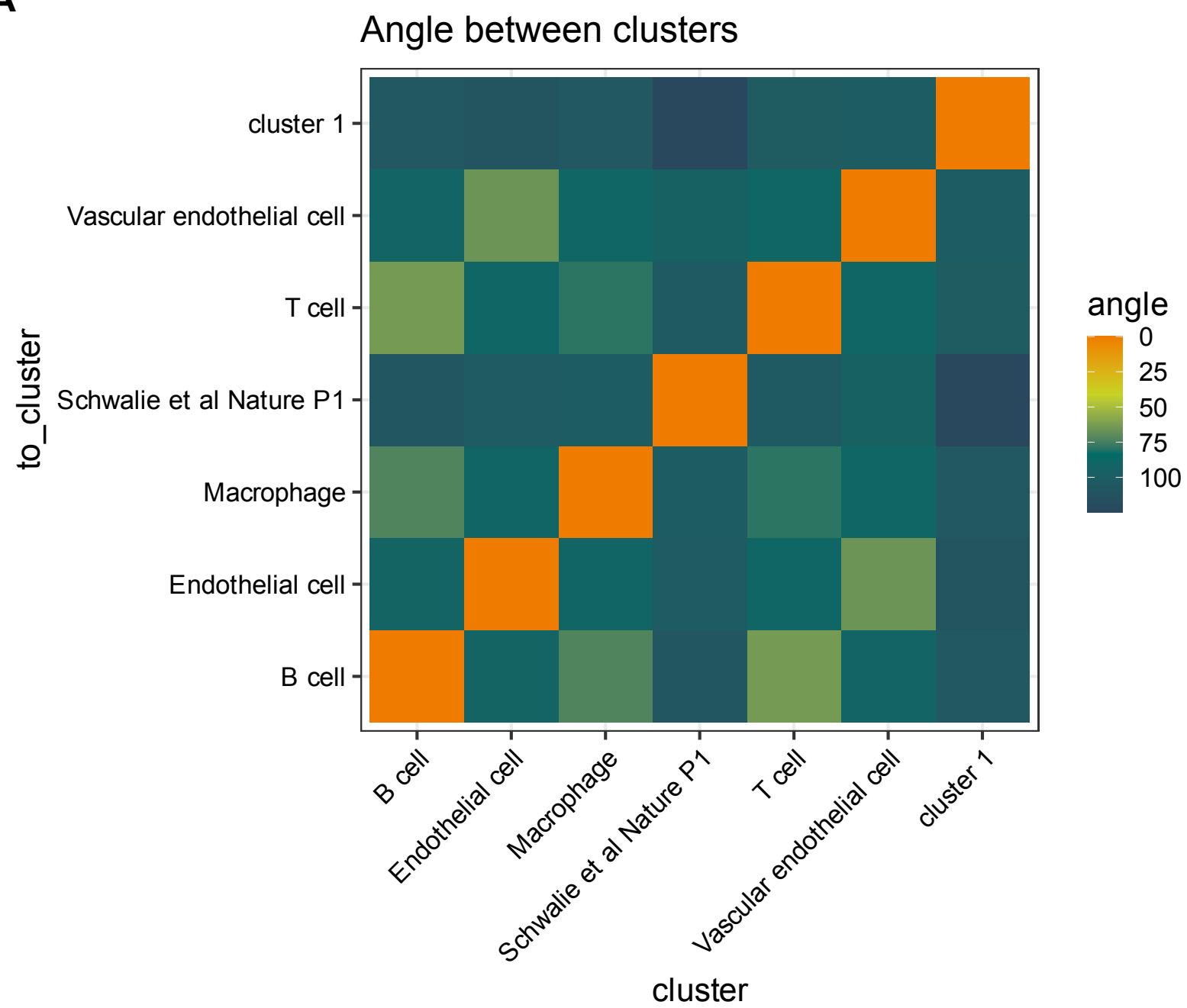**B**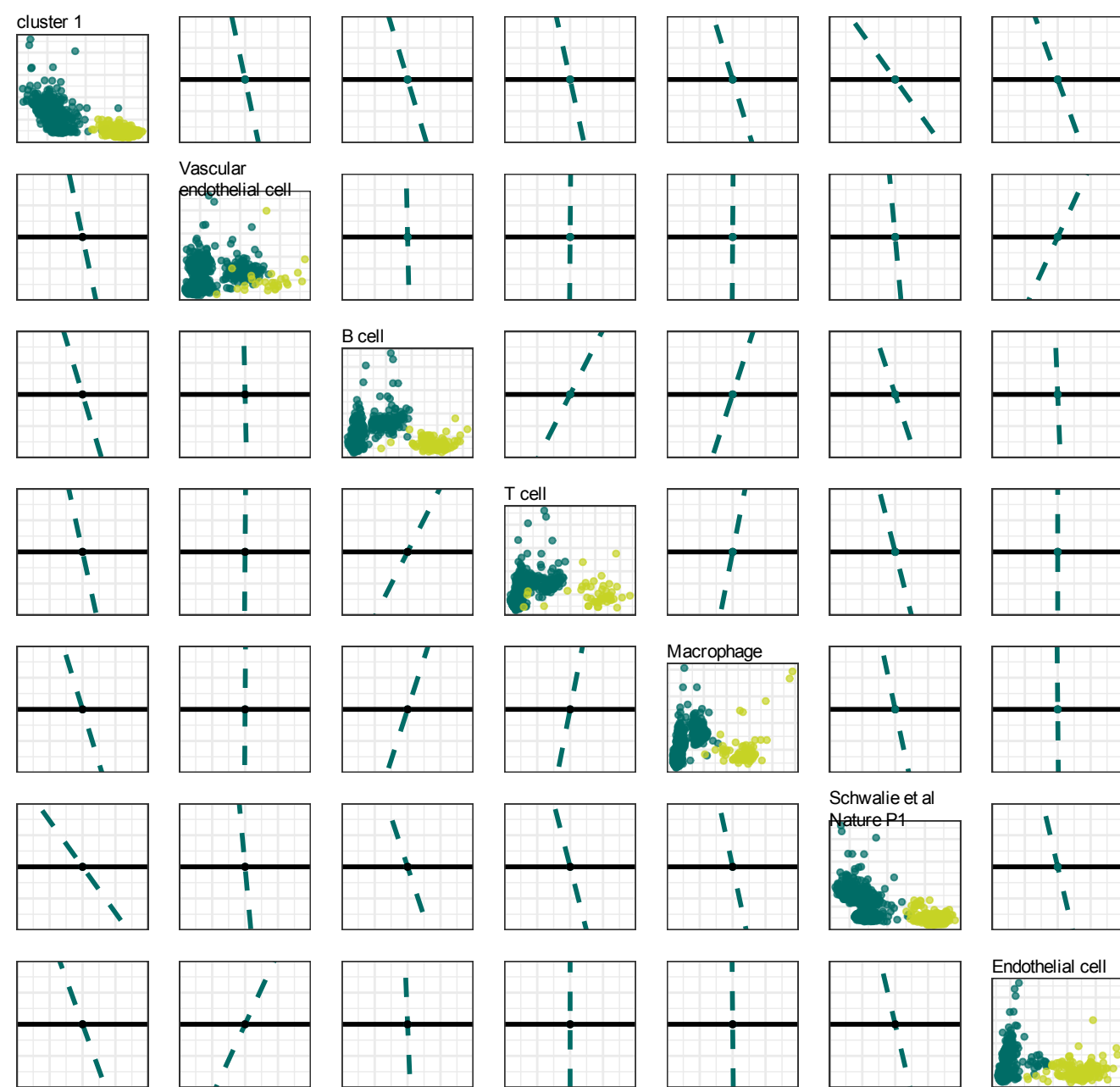**C**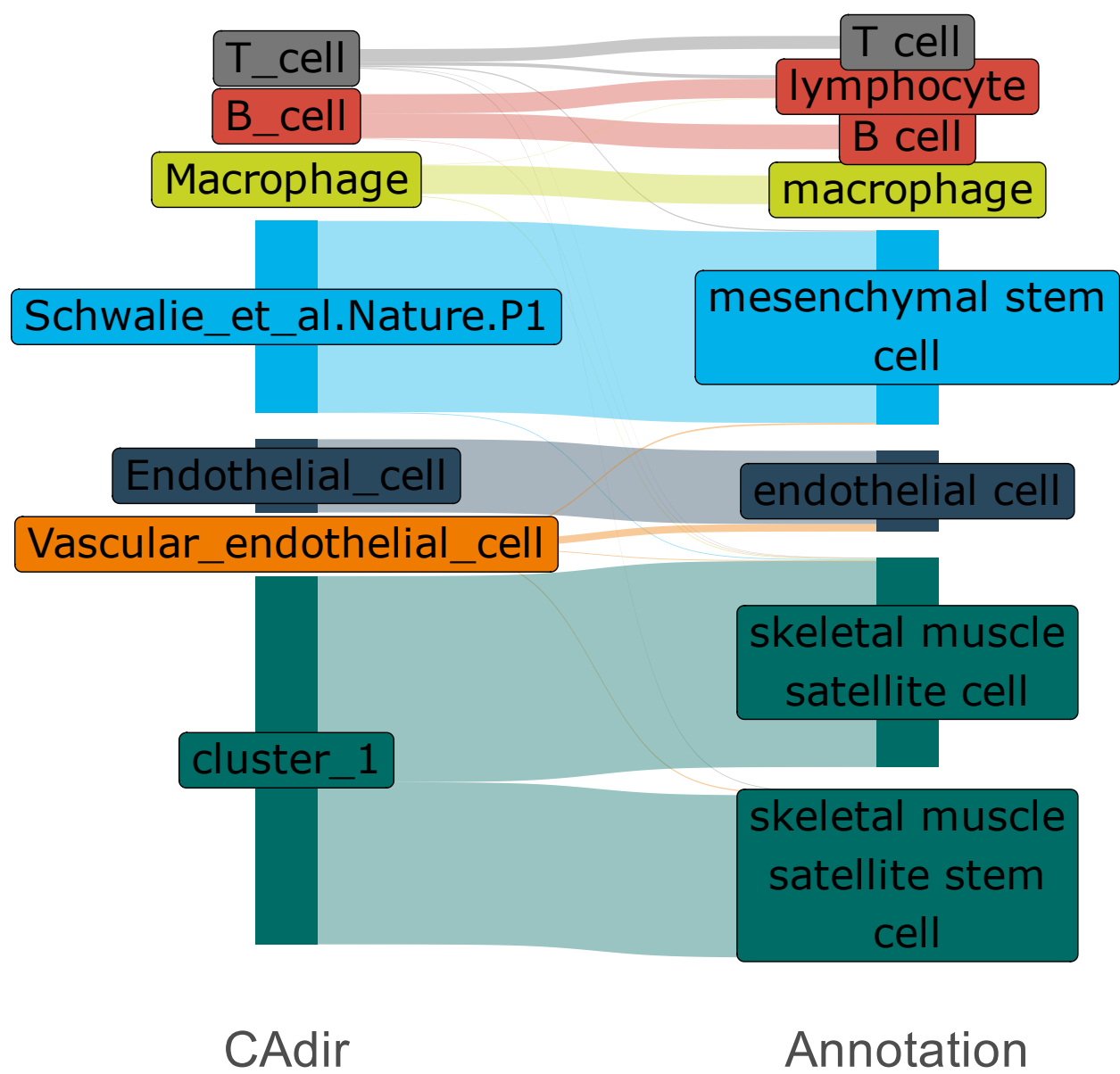**D**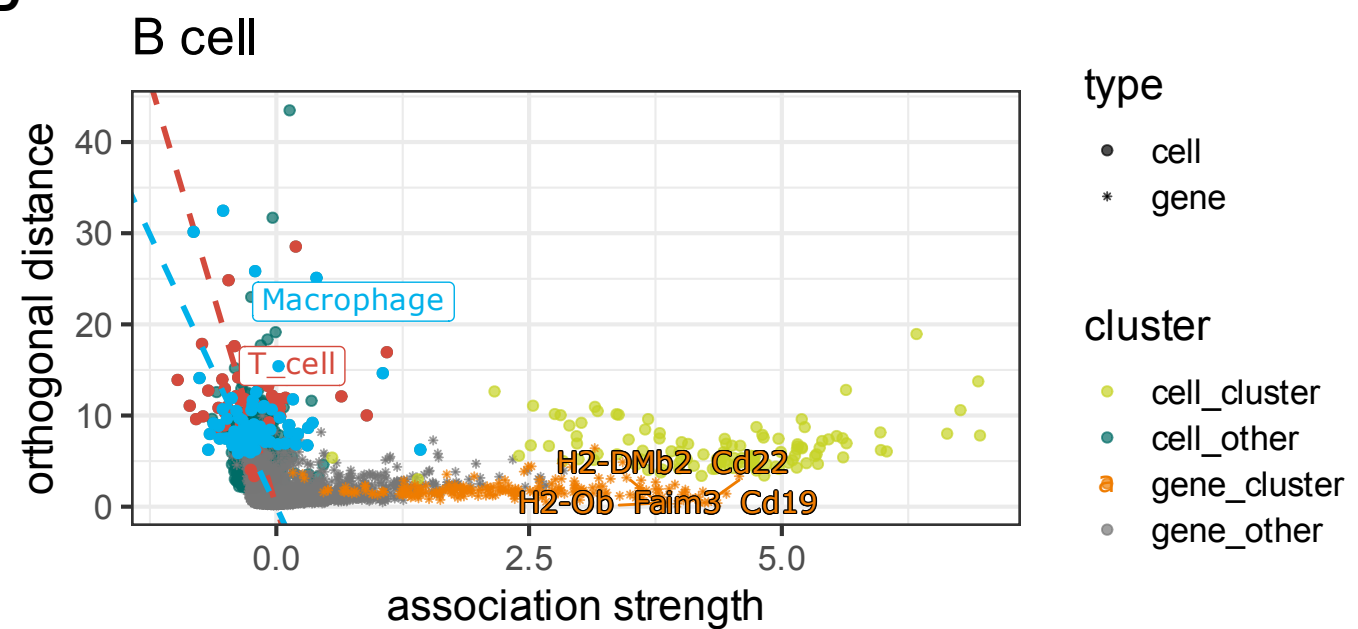**E**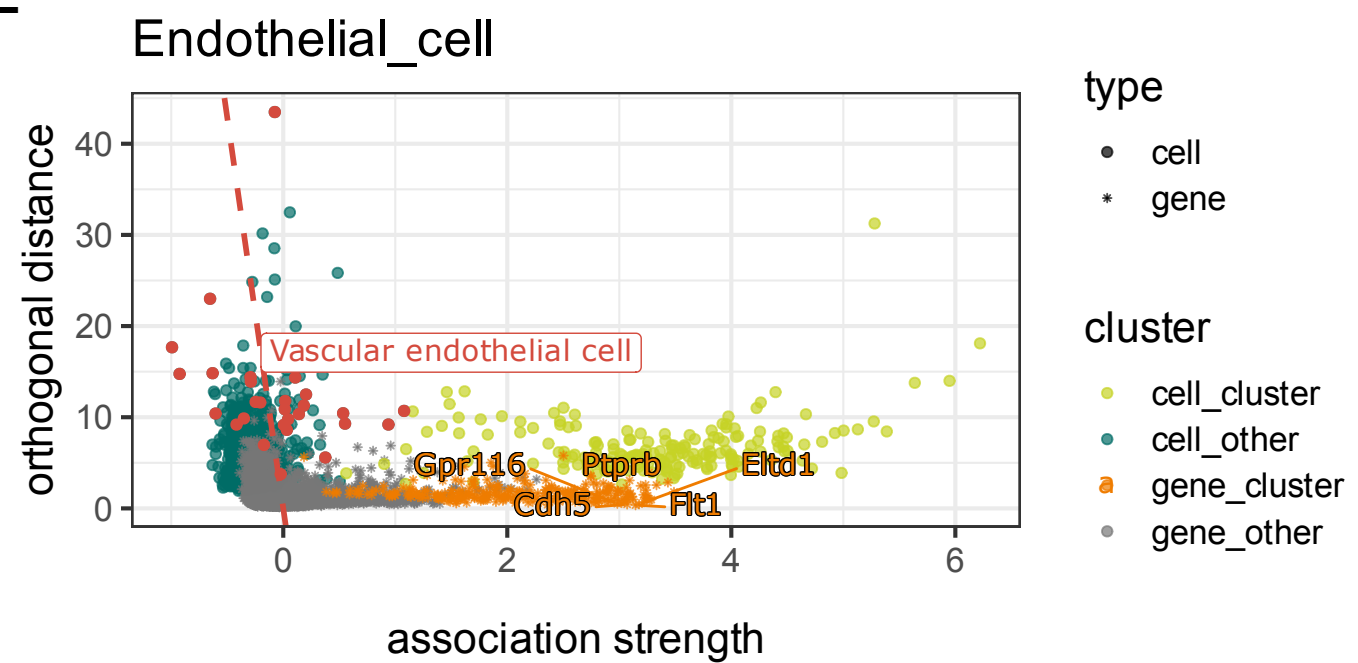

Supplement: S1 Fig — A, Matrix of the pairwise angles between the cluster directions. Lower angles indicate higher similarity between clusters. B, Each row shows an Association Plot of the respective cluster in the diagonal and lines that show the direction of the remaining other clusters projected into the Association Plot. C, Sankey plot of the annotated clustering obtained with CAdir (left) and the ground truth annotation (right). Association Plot with cells in standard coordinates for D, B cells and E, Endothelial cells. Closely associated clusters are colored in red and blue. (PDF) [file pcbi.1014418.s002.pdf]

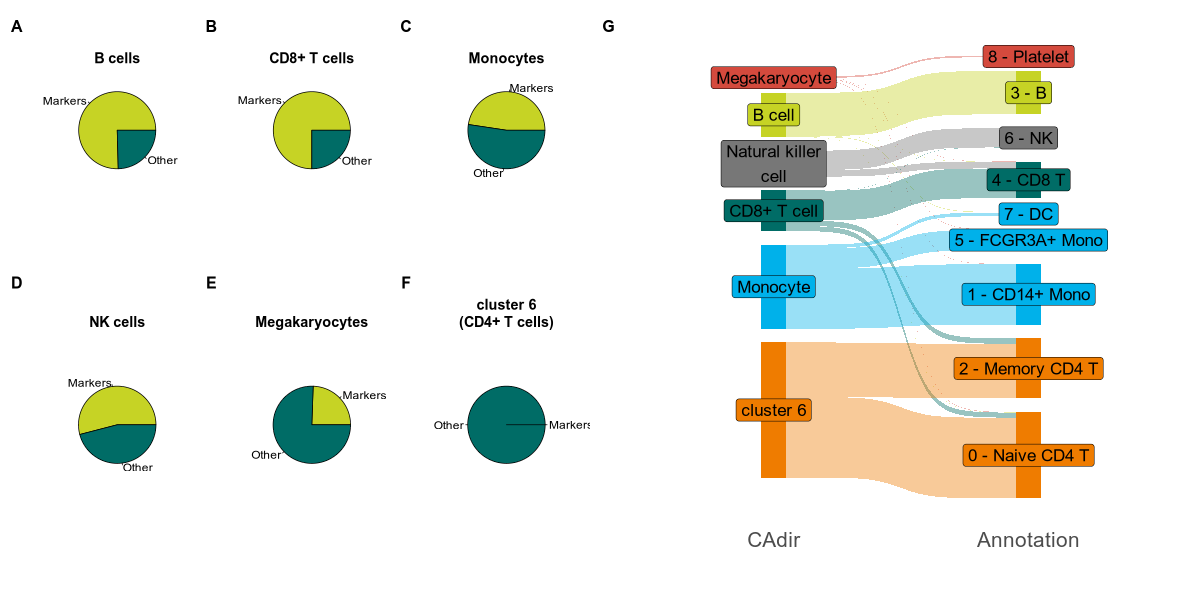

Supplement: S3 Fig — Clustering of the PBMC3k data using CAdir. A-E, Proportion of co-clustered genes with Sθ-score > 0 that are also contained in the CellMarker gene set used to annotate the cluster. F, For cluster 6 the gene set corresponding to the corresponding cell type from the annotation was used. G, Sankey plot of the clustering to show correspondence to the reference annotation. (PNG) [file pcbi.1014418.s004.png]

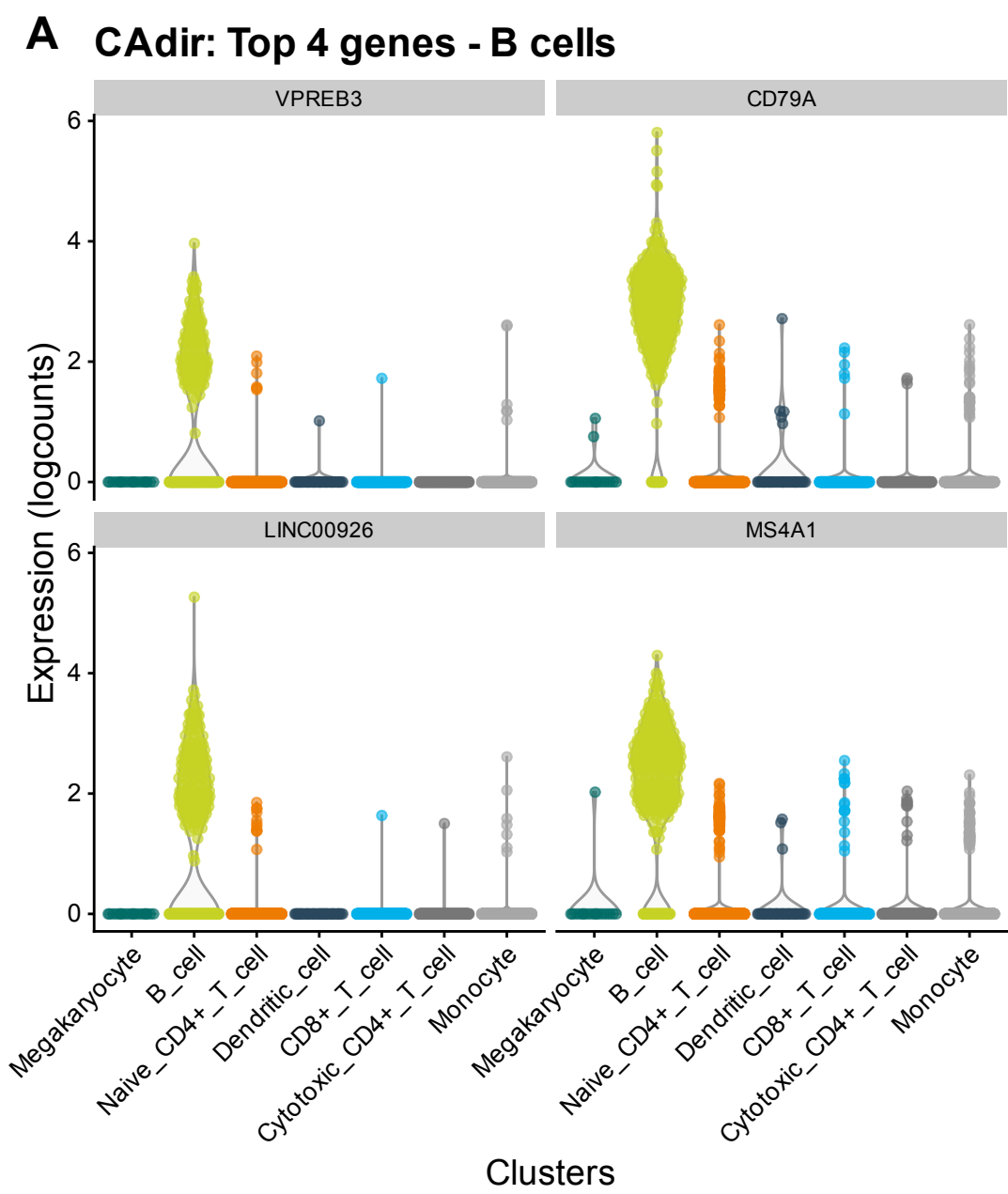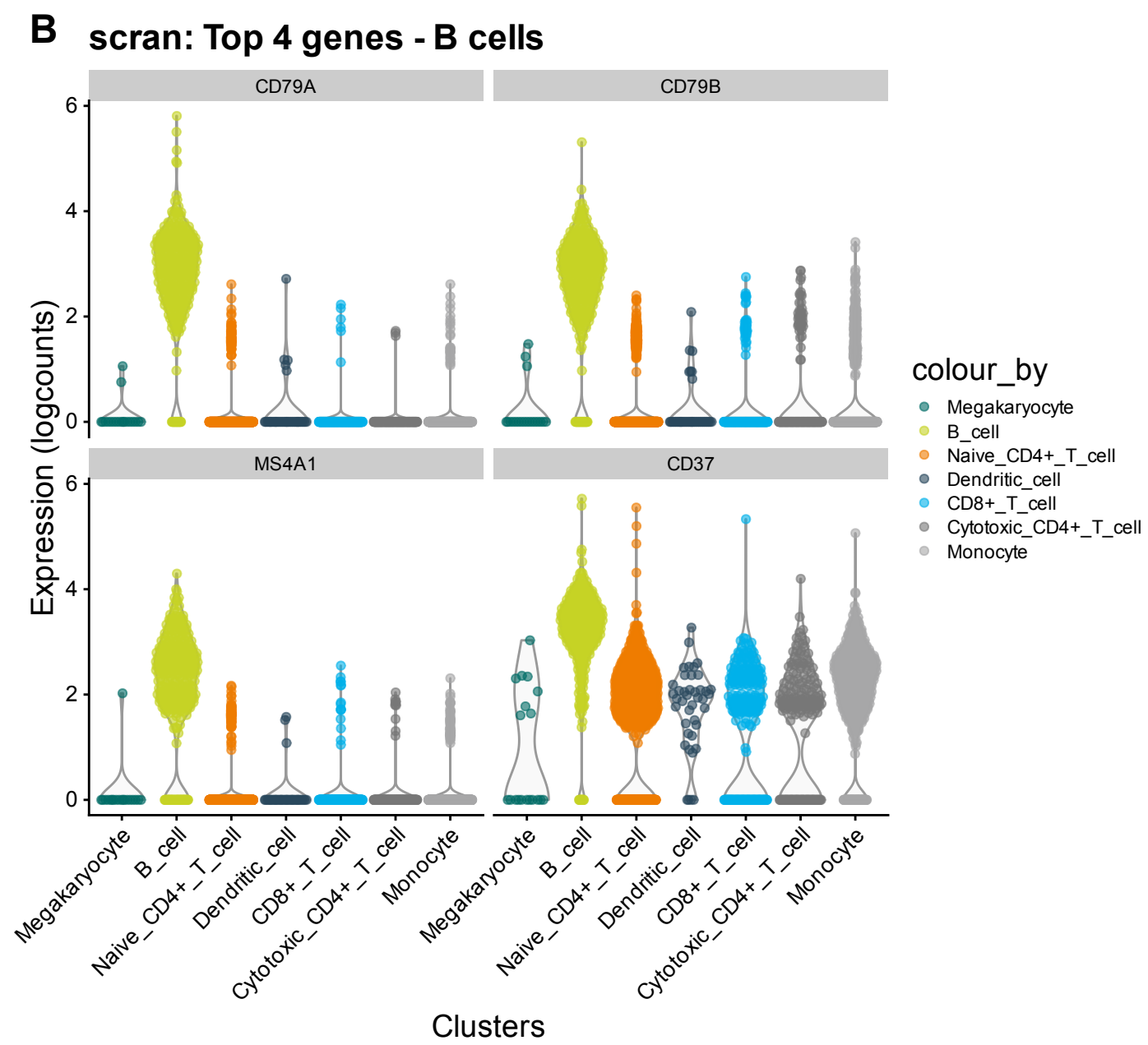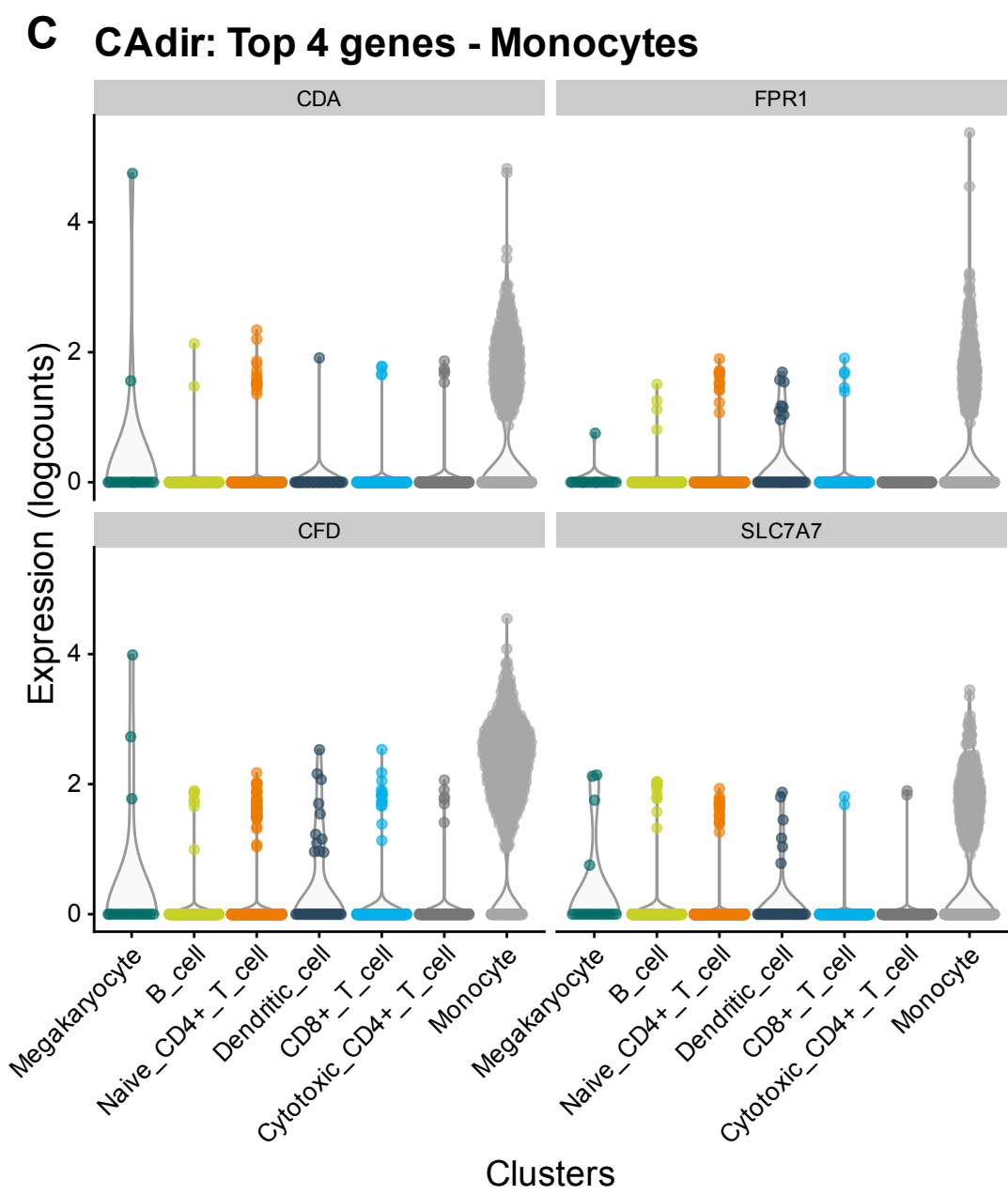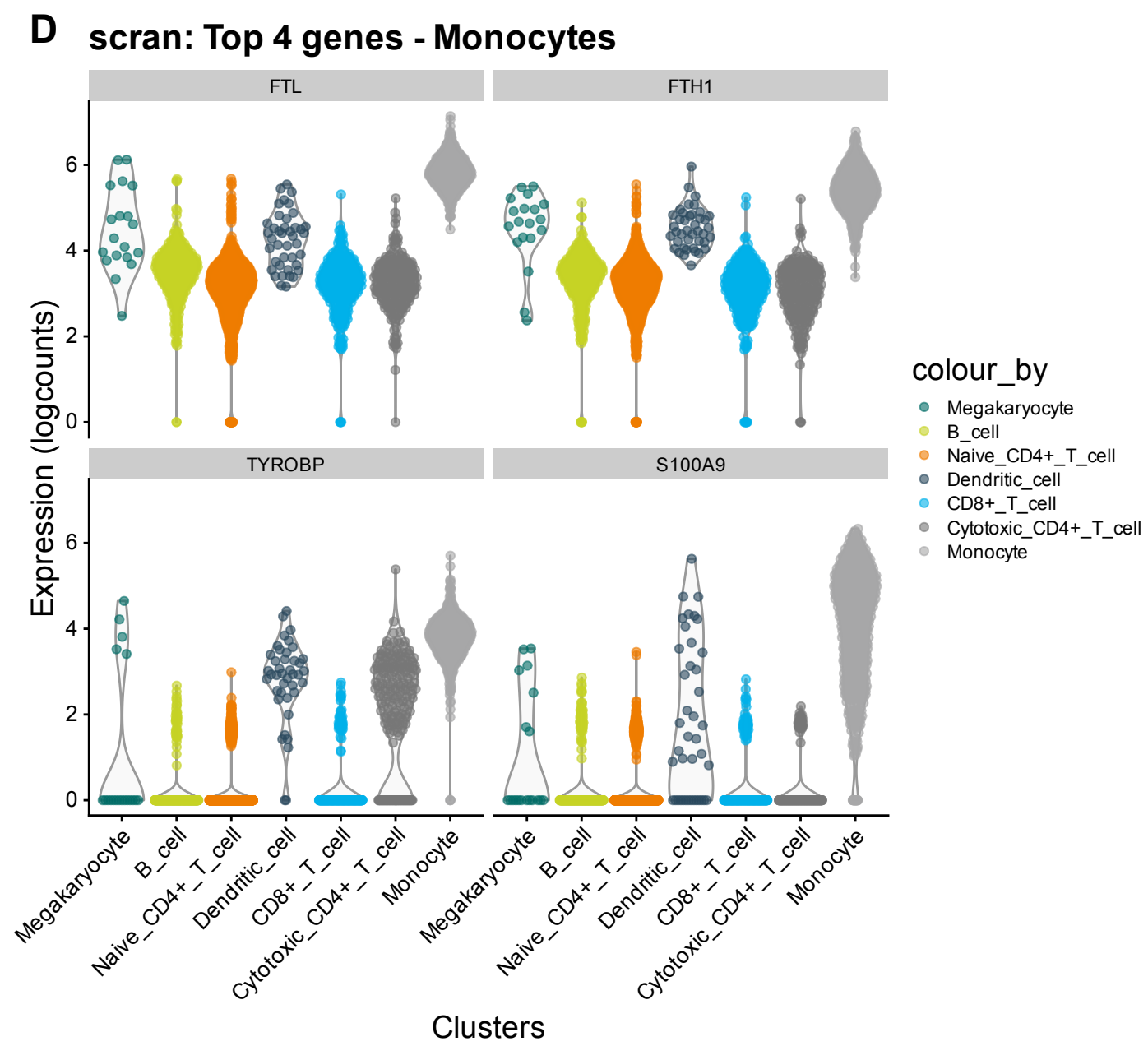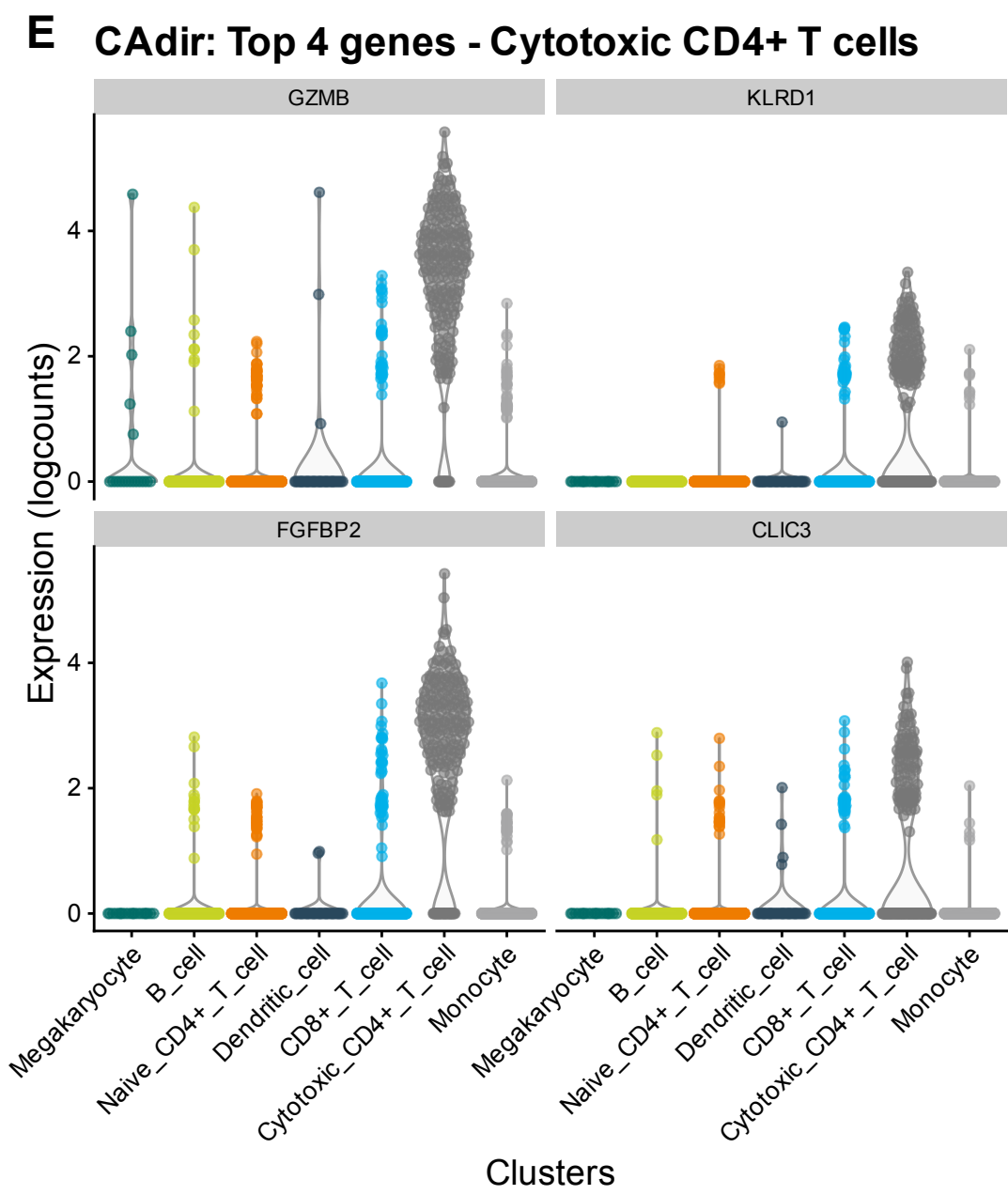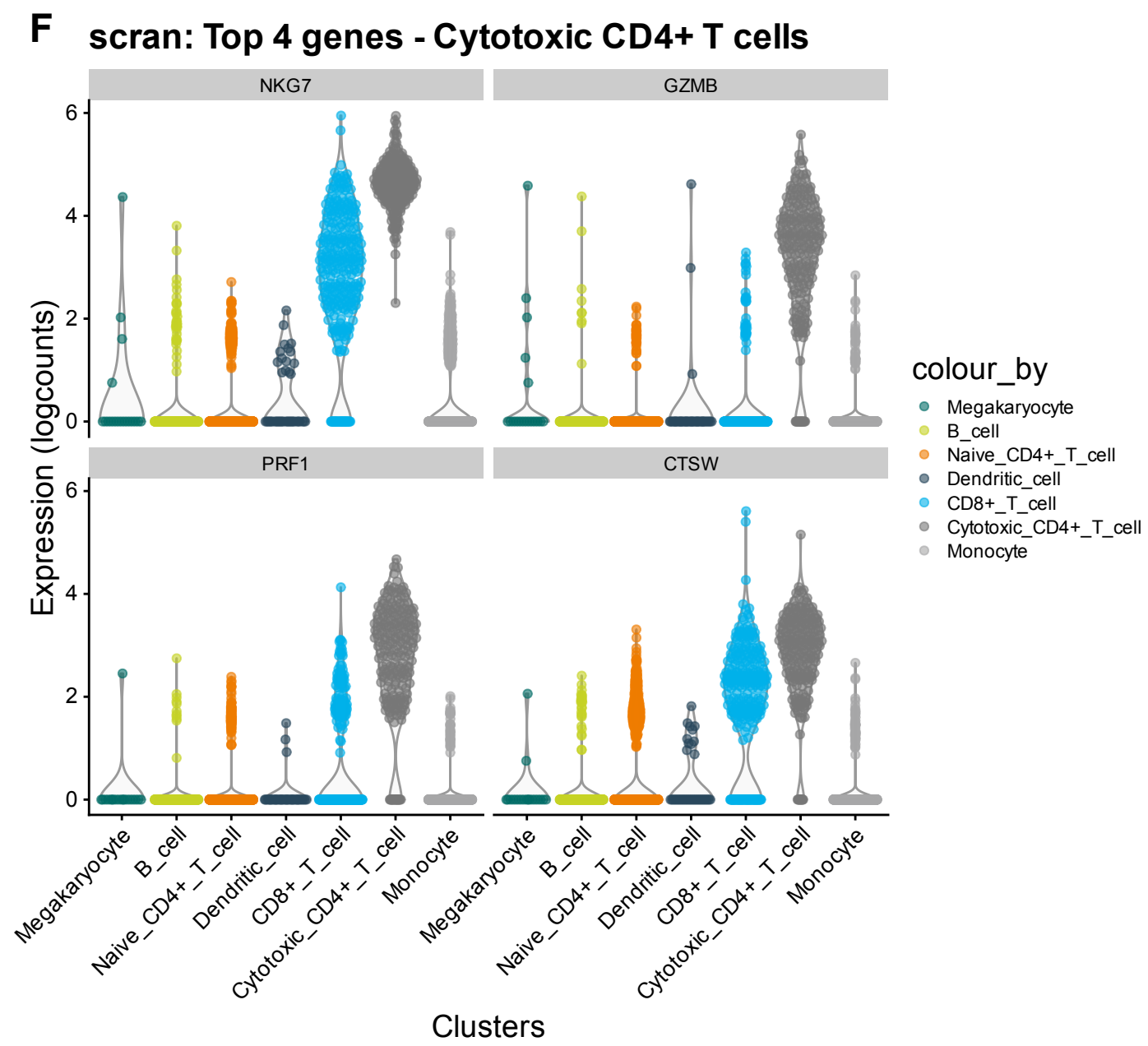

Supplement: S4 Fig — The violin plots show the expression for the 4 highest ranked genes using the Sθ-score for CAdir and the mean AUC for scran. A-B, Expression for the B cell cluster. C-D, Expression for the Monocytes cluster. E-F, Expression for the Cytotoxic CD4+ T cell cluster. (PDF) [file pcbi.1014418.s005.pdf]

**A** Mean NMI on simulated data

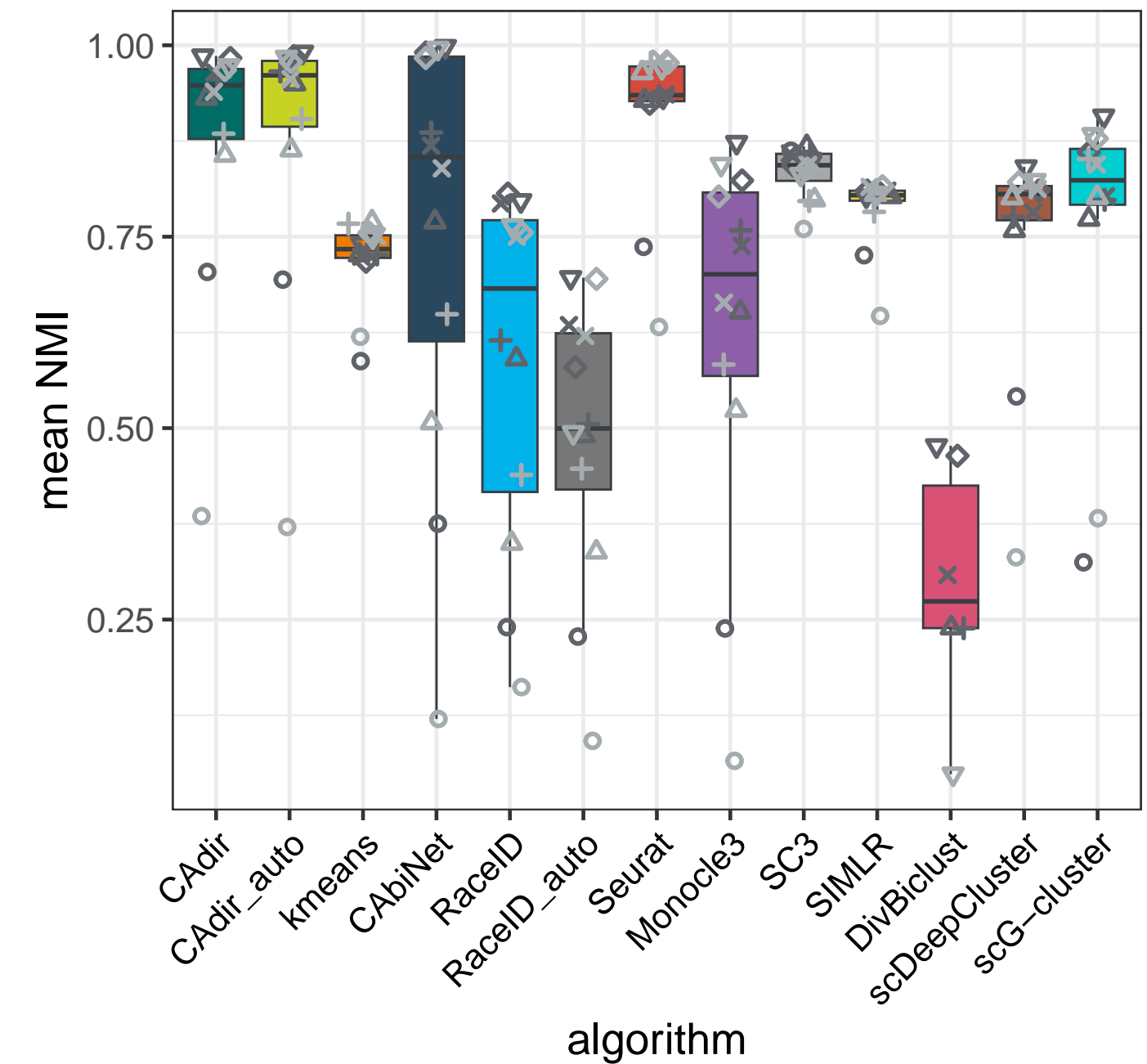

**B** Fraction of successful and failed runs

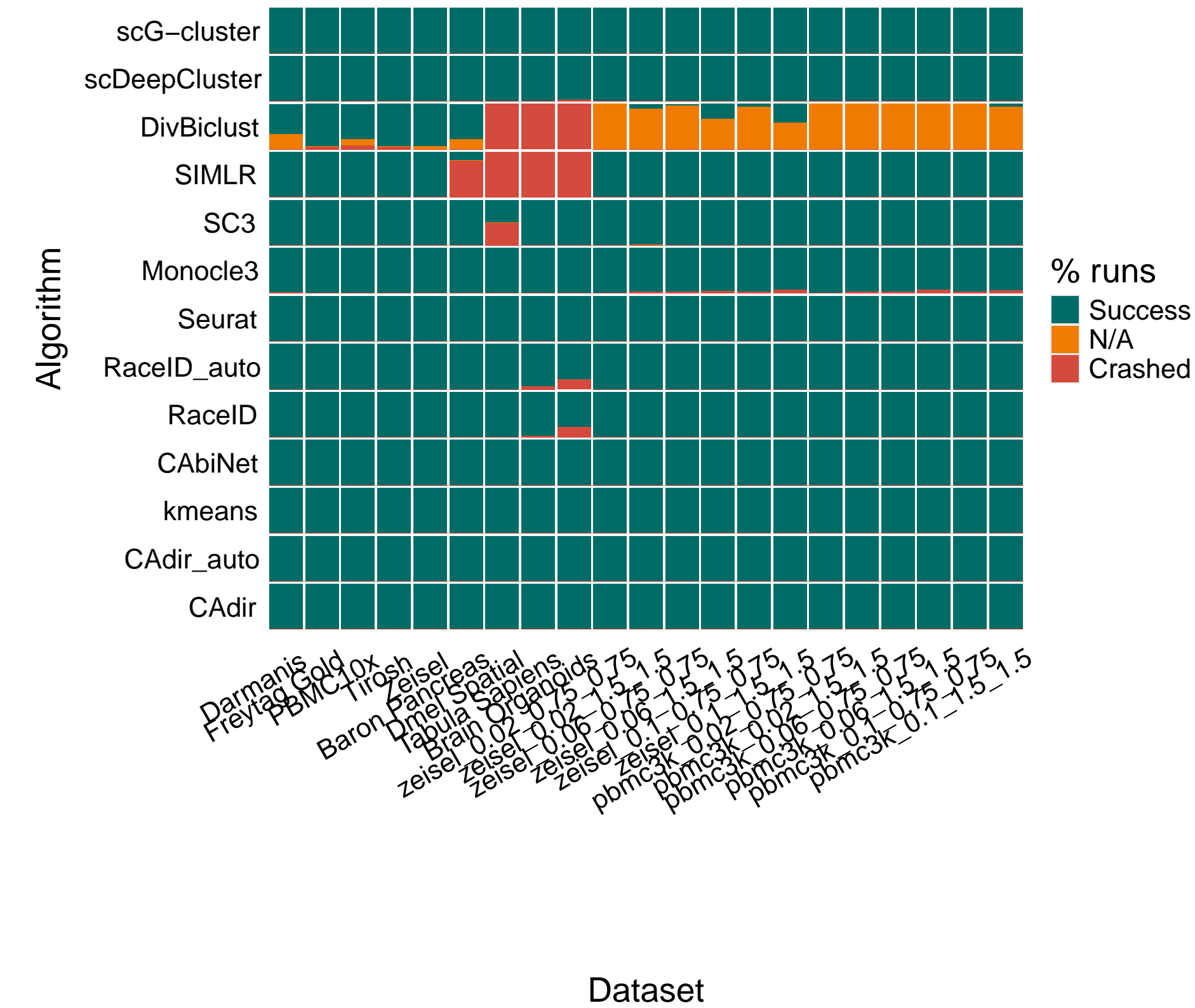

Supplement: S6 Fig — A, Mean Normalized Mutual Information over all 108 parameter combinations for each simulated data set. B, Fraction of successful (dark green) runs, runs that produced N/A (not assigned, orange) and crashed runs (red) for each algorithm and tested data set. (PDF) [file pcbi.1014418.s007.pdf]

**A****Tirosh**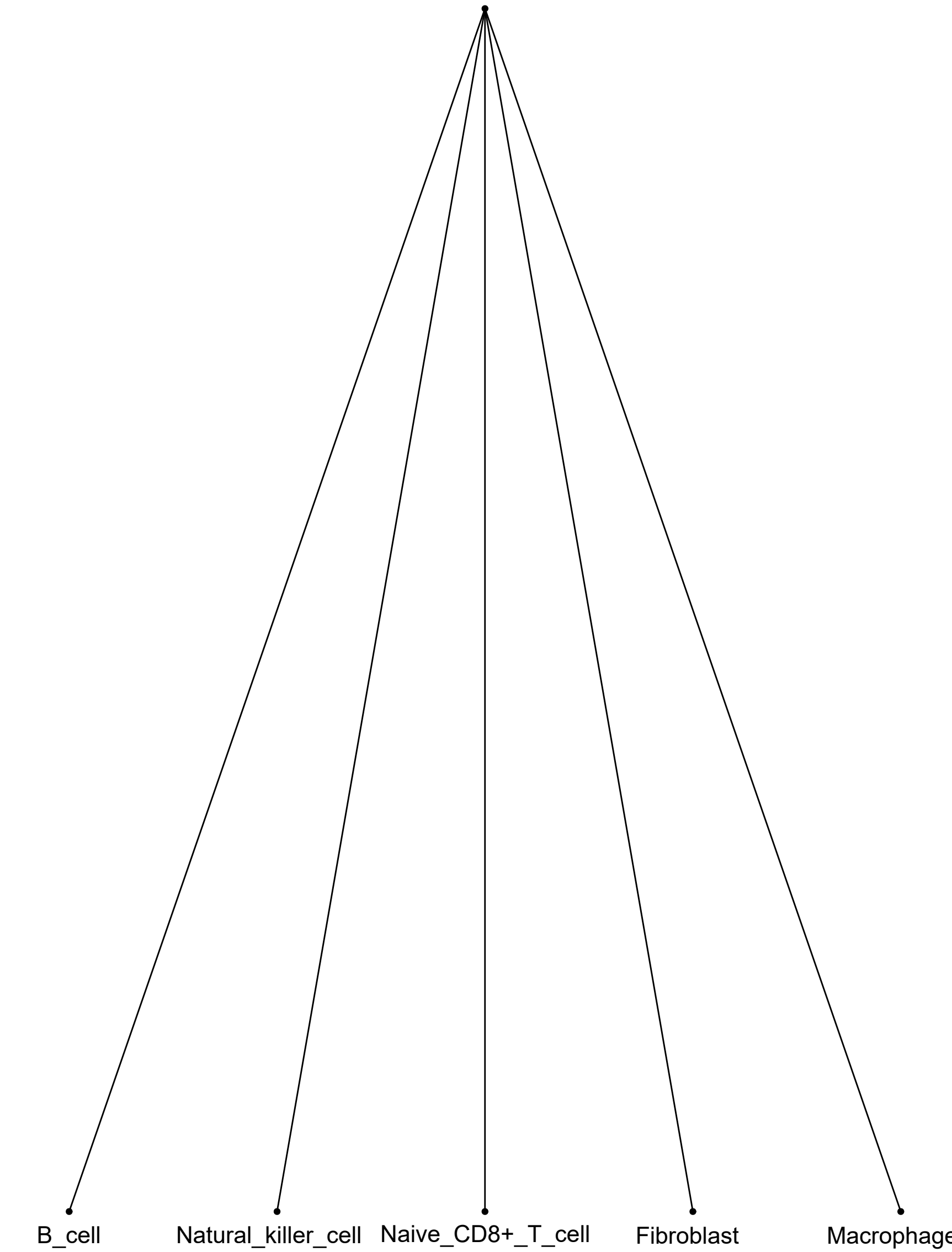**B****Brain Organoids**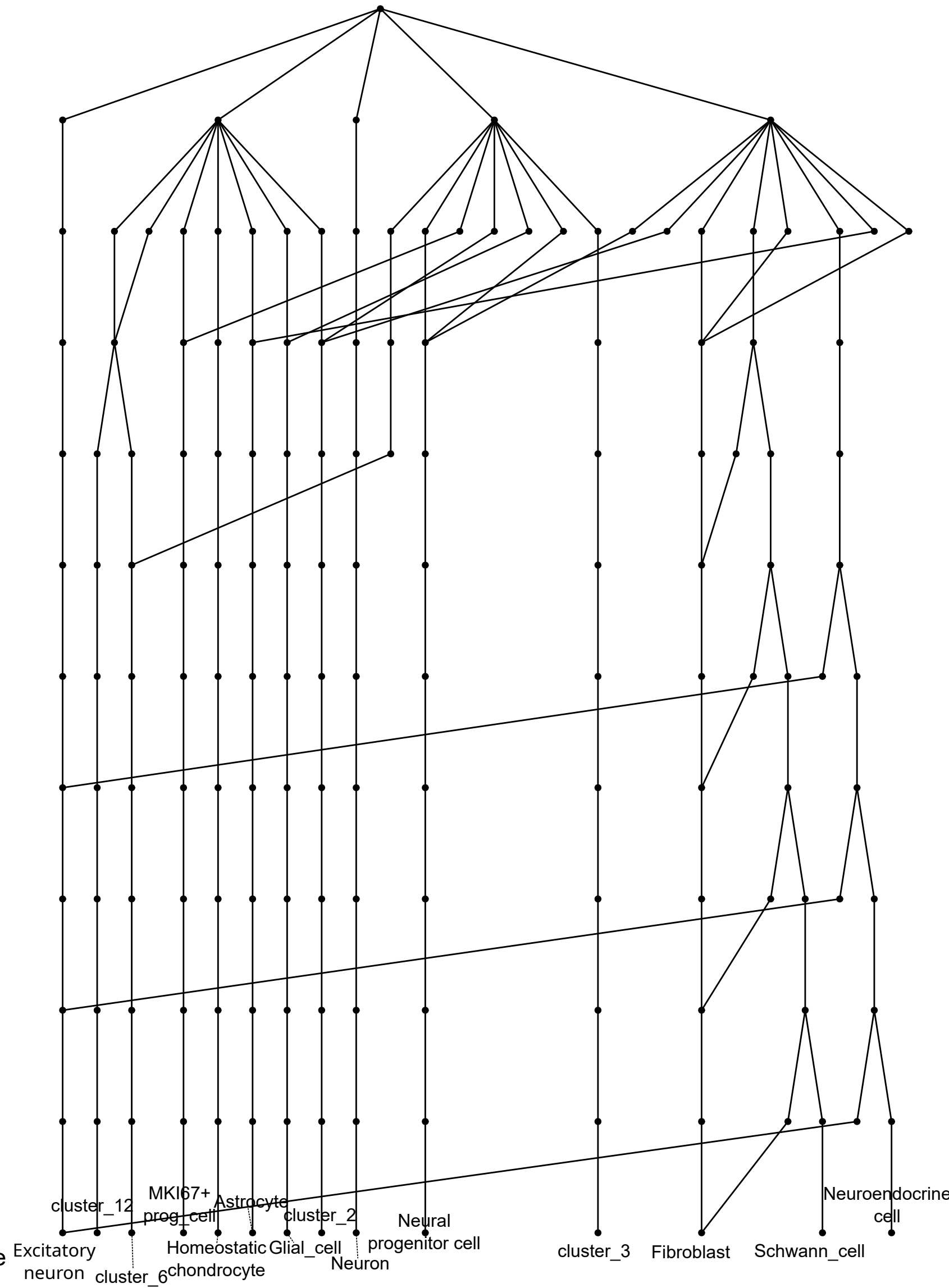**C****Tabula Sapiens**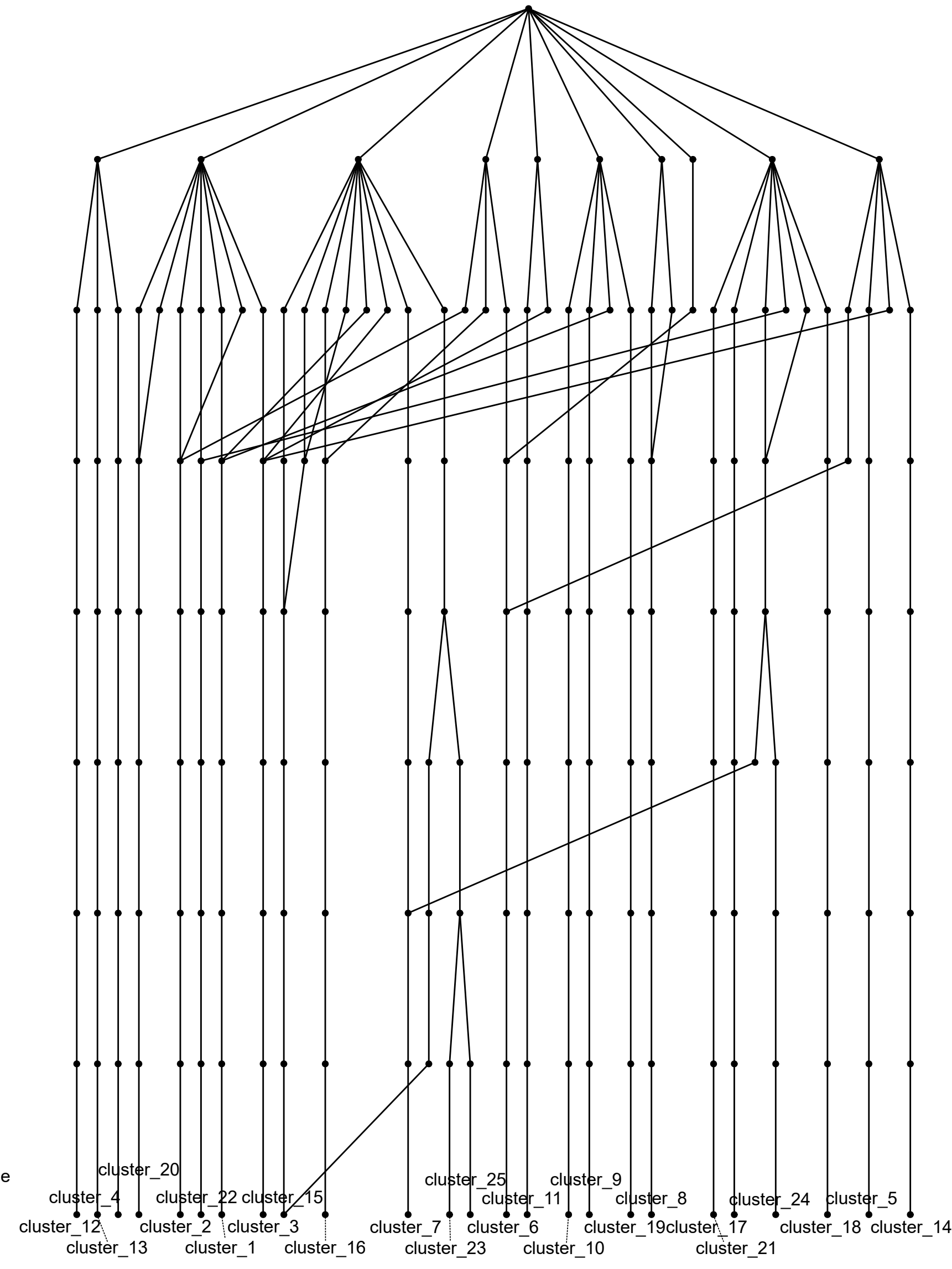

Supplement: S8 Fig — For the cell clustering parameter combination that achieved the best ARI score, split-merge graphs are plotted for the A, Tirosh B, Brain Organoids and C, Tabula Sapiens data set. (PDF) [file pcbi.1014418.s009.pdf]

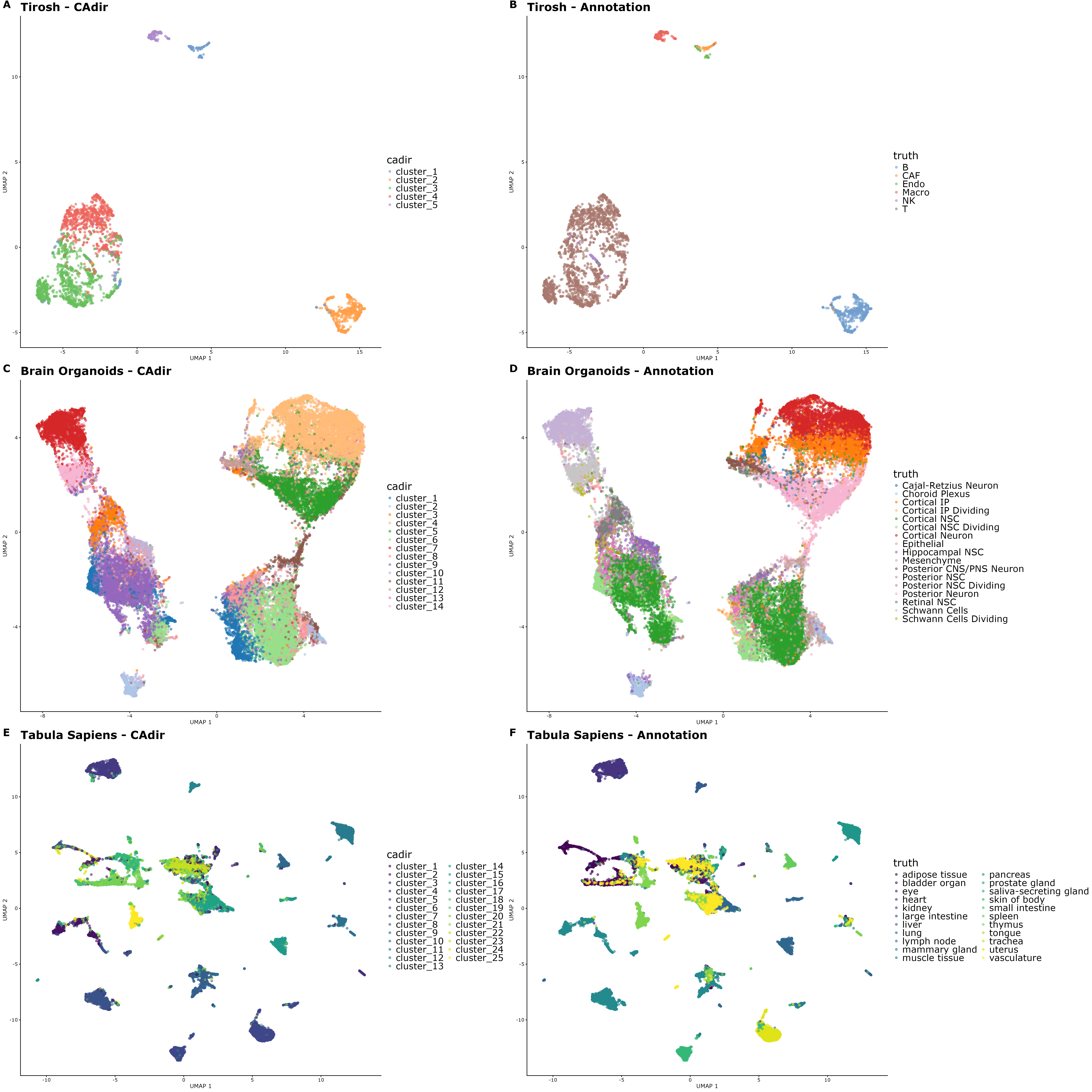

Supplement: S9 Fig — Using the best scoring parameter combination of the cell clustering comparison, UMAPs for both the CAdir clustering and the reference annotation are plotted for the A-B, Tirosh, C-D, Brain Organoids and E-F, Tabula Sapiens. These data sets were chosen to further understand why CAdir did not achieve the best clustering results in the benchmarking on these data sets. (PNG) [file pcbi.1014418.s010.png]

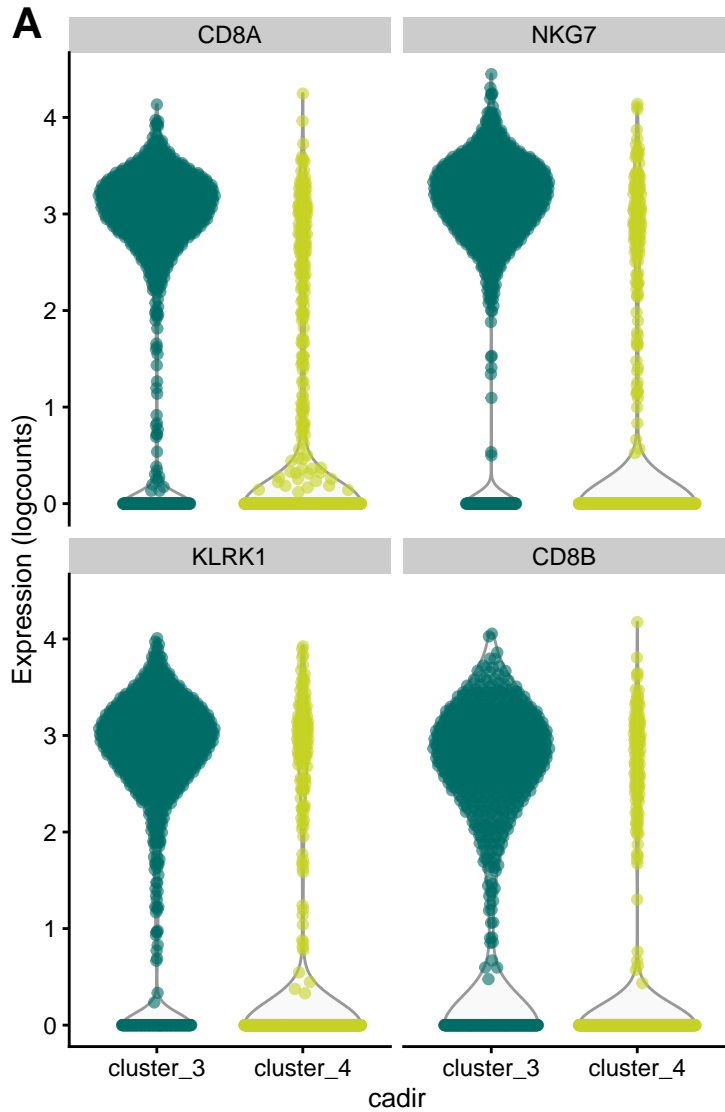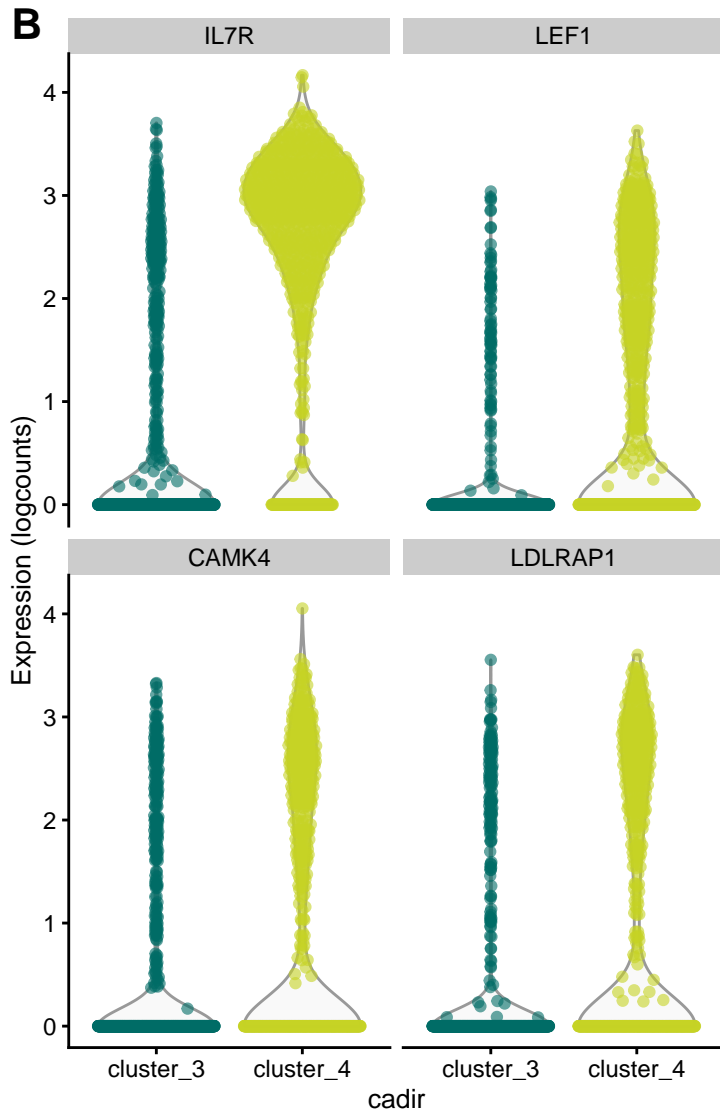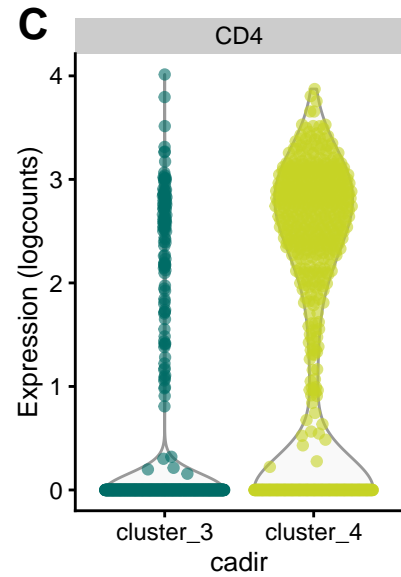

Supplement: S10 Fig — Expression levels of the 4 genes with the highest Sθ-score are shown for A, cluster 3 and B, cluster 4 using the best performing parameter combinations from the cell clustering benchmarking of CAdir on the Tirosh data set. C, Expression of CD4 in cluster 3 and 4. (PDF) [file pcbi.1014418.s011.pdf]

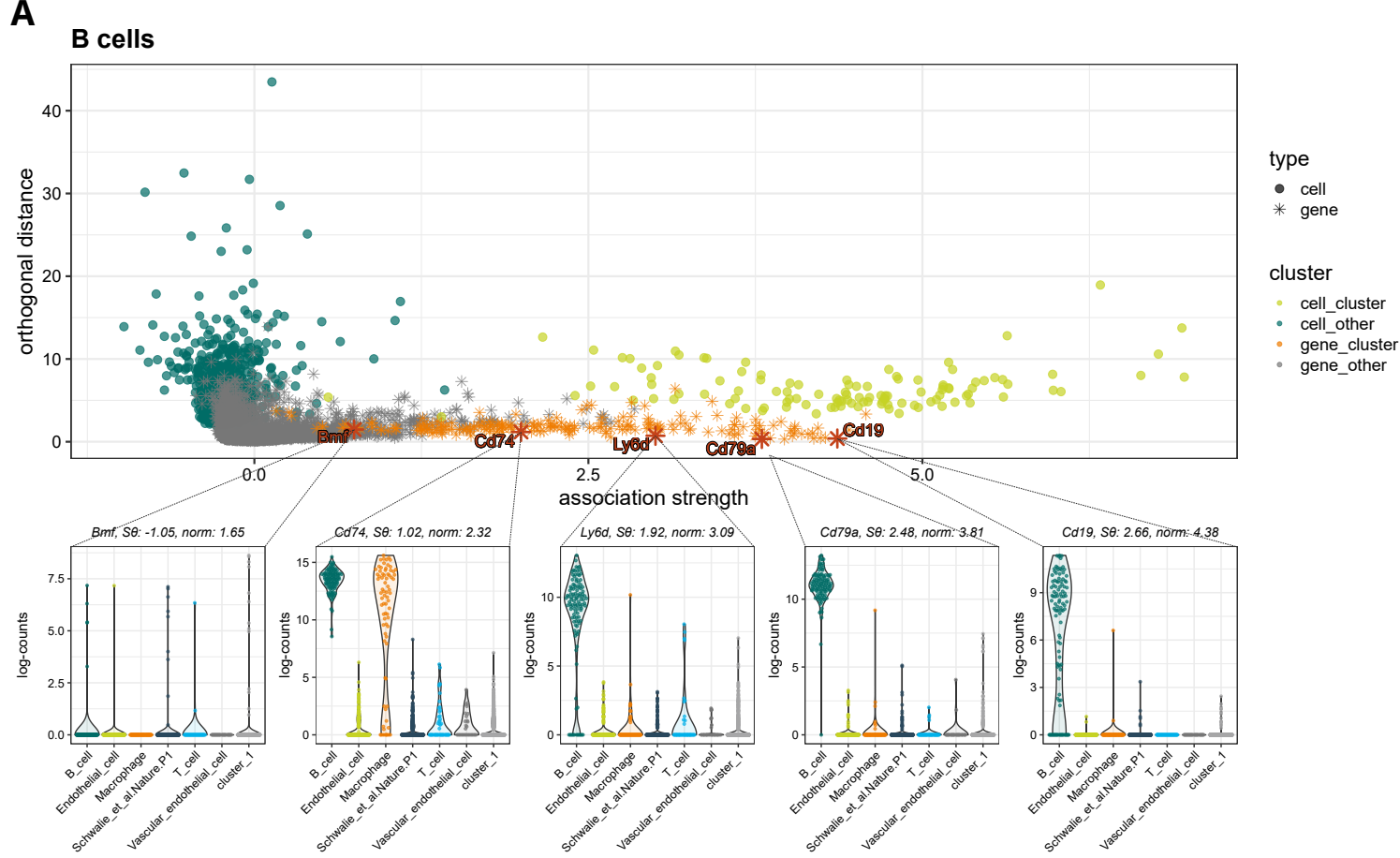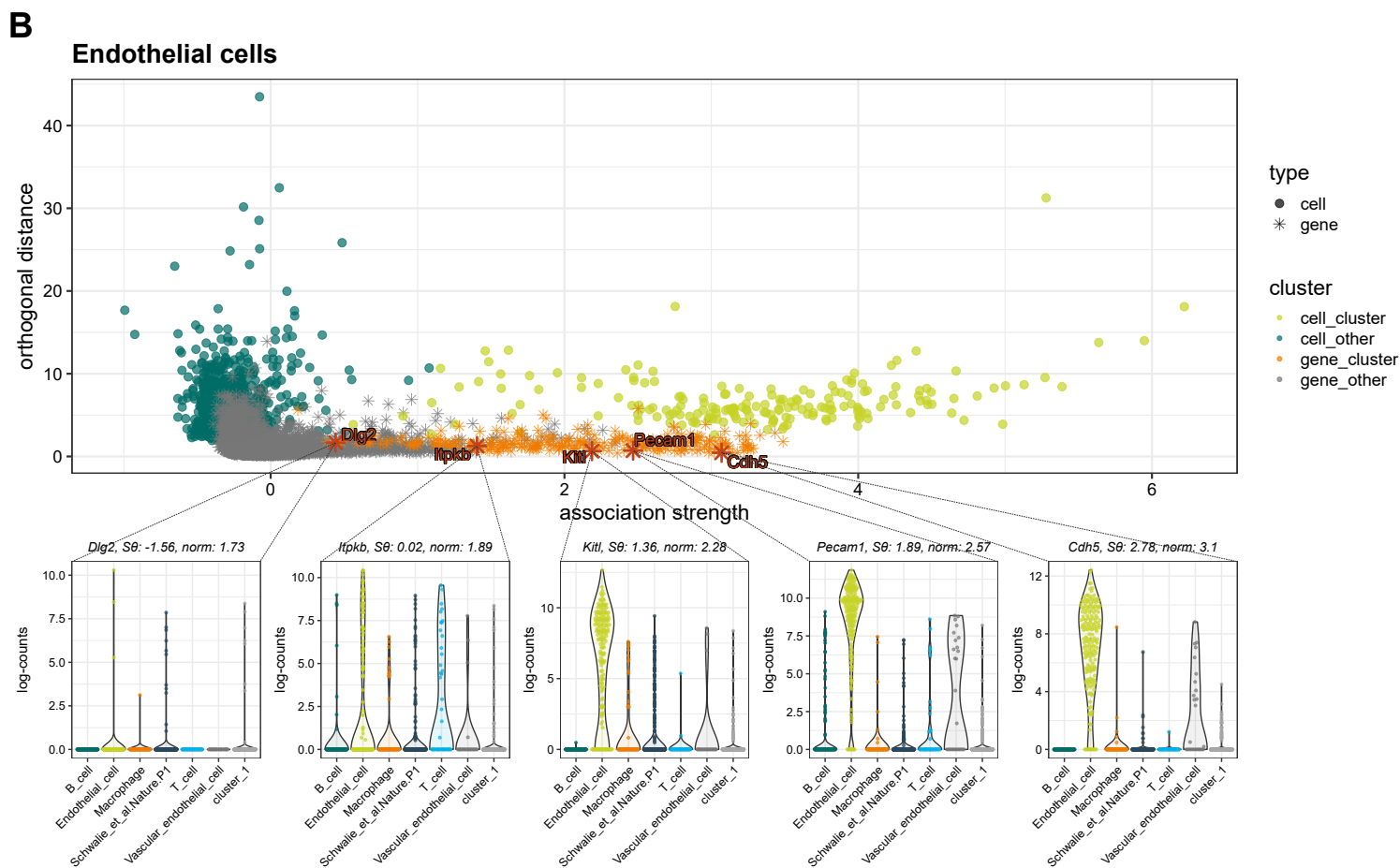

Supplement: S14 Fig — Association Plots of the A, B cell B, Endothelial cell clusters of the Tabula Muris Limb Muscle data. The selected genes increase in both their vector norm and Sθ-score from left to right. The violin plots for the selected genes show the log-normalized expression of the genes in the clusters. As shown, the further out the gene lies from the origin, the more cluster specific they are. (PDF) [file pcbi.1014418.s015.pdf]

# Detected clusters

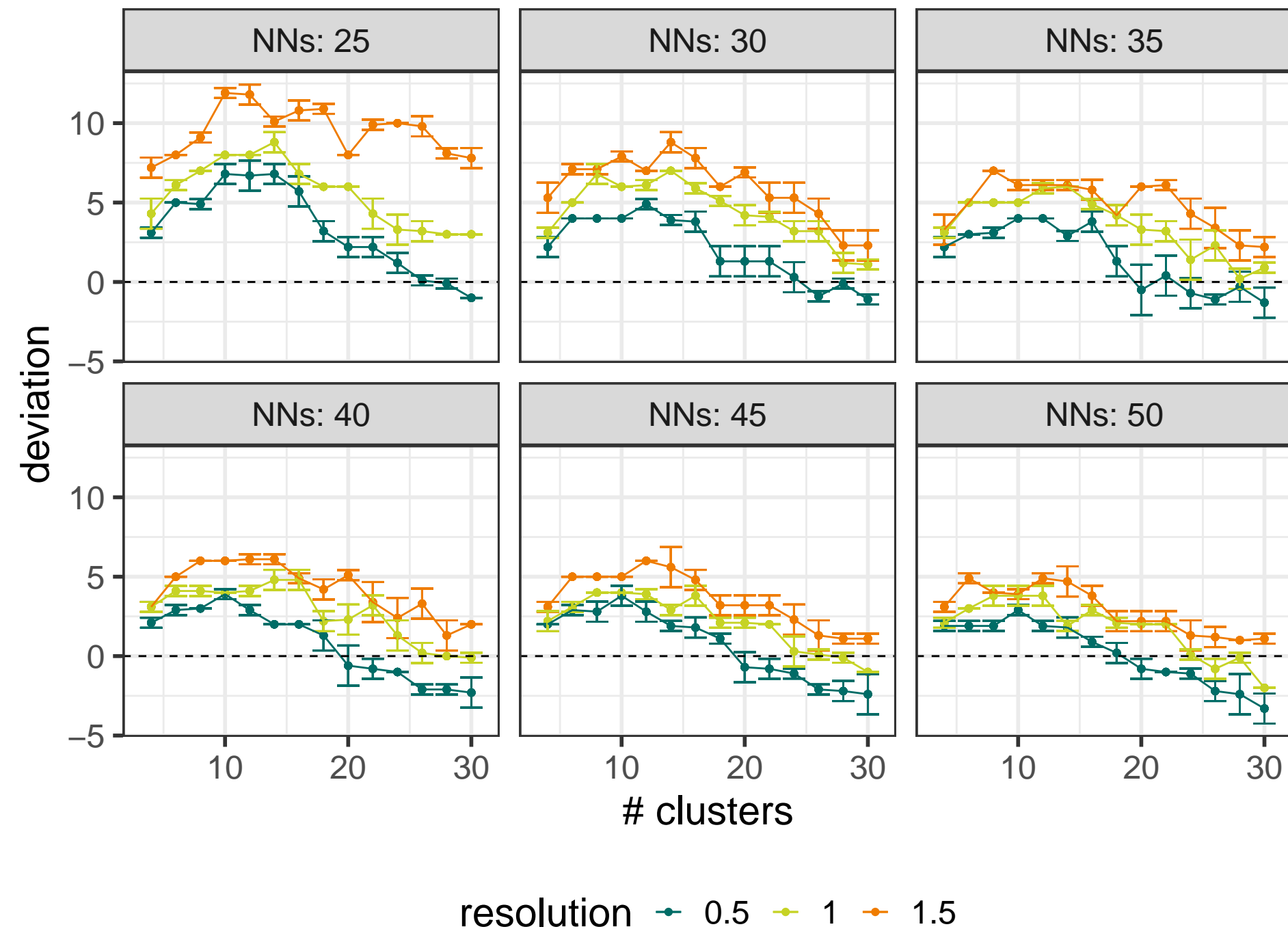

Supplement: S15 Fig — Deviation of the number of recovered clusters using Seurat (y-axis) compared to the number of randomly sampled clusters (x-axis) from the Tabula Muris cell atlas. The deviation was measured for different numbers of nearest neighbors (NNs) as well as for different Leiden clustering resolutions. (PDF) [file pcbi.1014418.s016.pdf]

**A**

Dropout Probability – Sparse Data

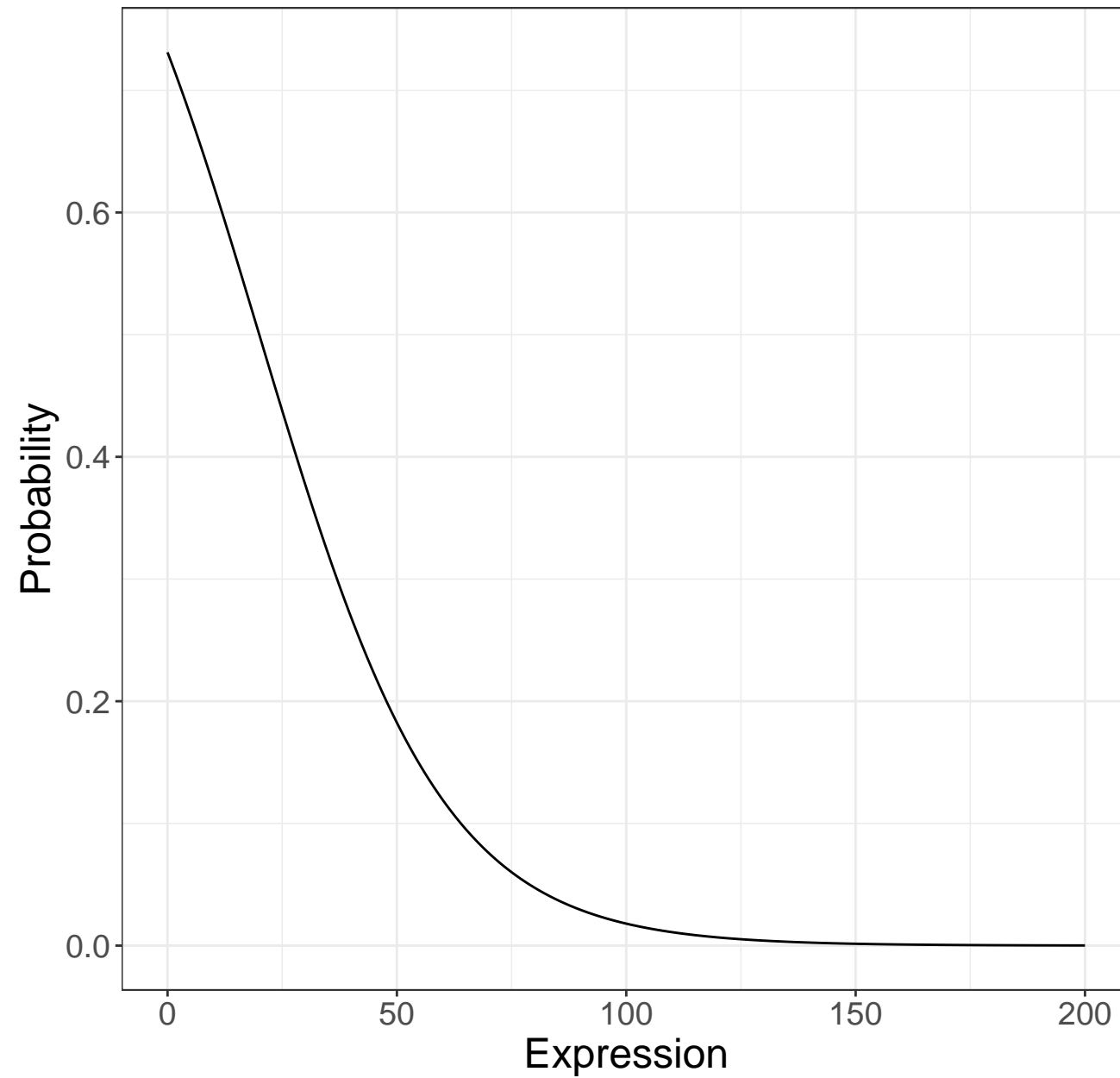**B**

Dropout Probability – Zeisel

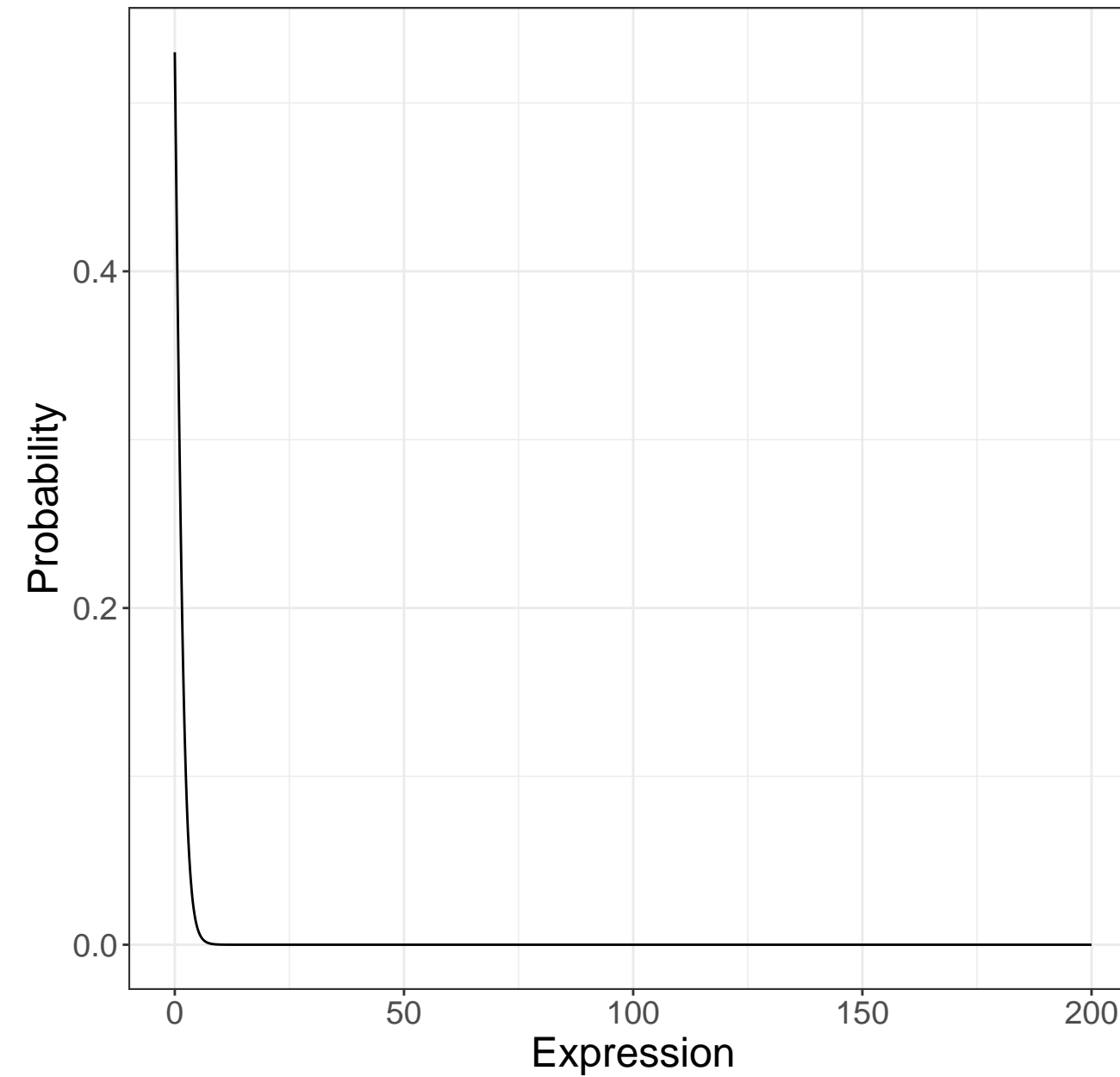**C**

Dropout Probability – PBMC3k

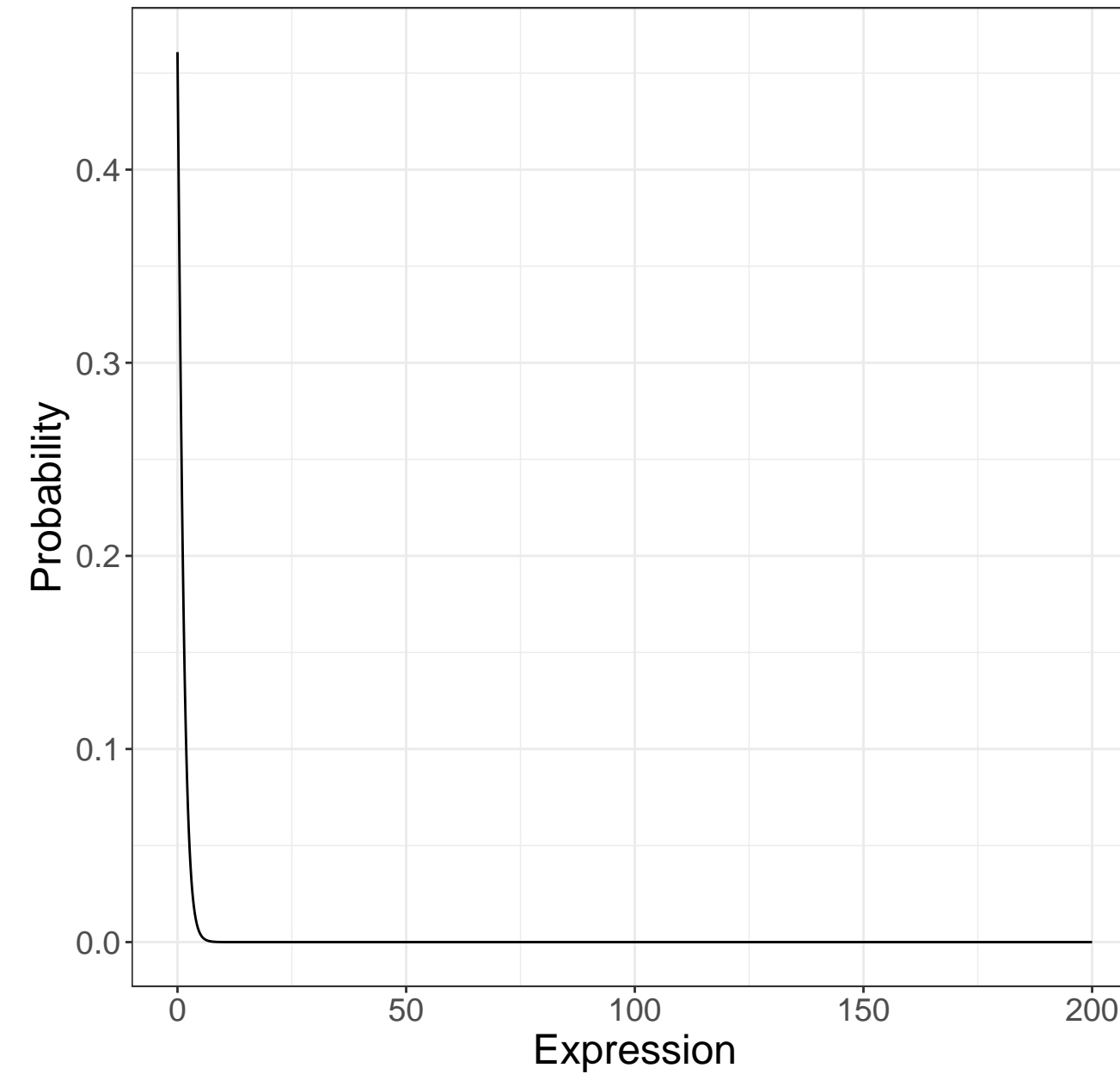

Supplement: S16 Fig — A, Logistic function with increased dropout probability. The simulation parameter dropout.mid that determines the point at which the probability for a gene to be a dropout is 0.5 is set to 20 and dropout.shape is set to -0.05 for a slower falloff. Logistic functions estimated from the data for b, the Zeisel and B, PBMC3k data set. (PDF) [file pcbi.1014418.s017.pdf]

**A**

Maximum ARI on simulated data

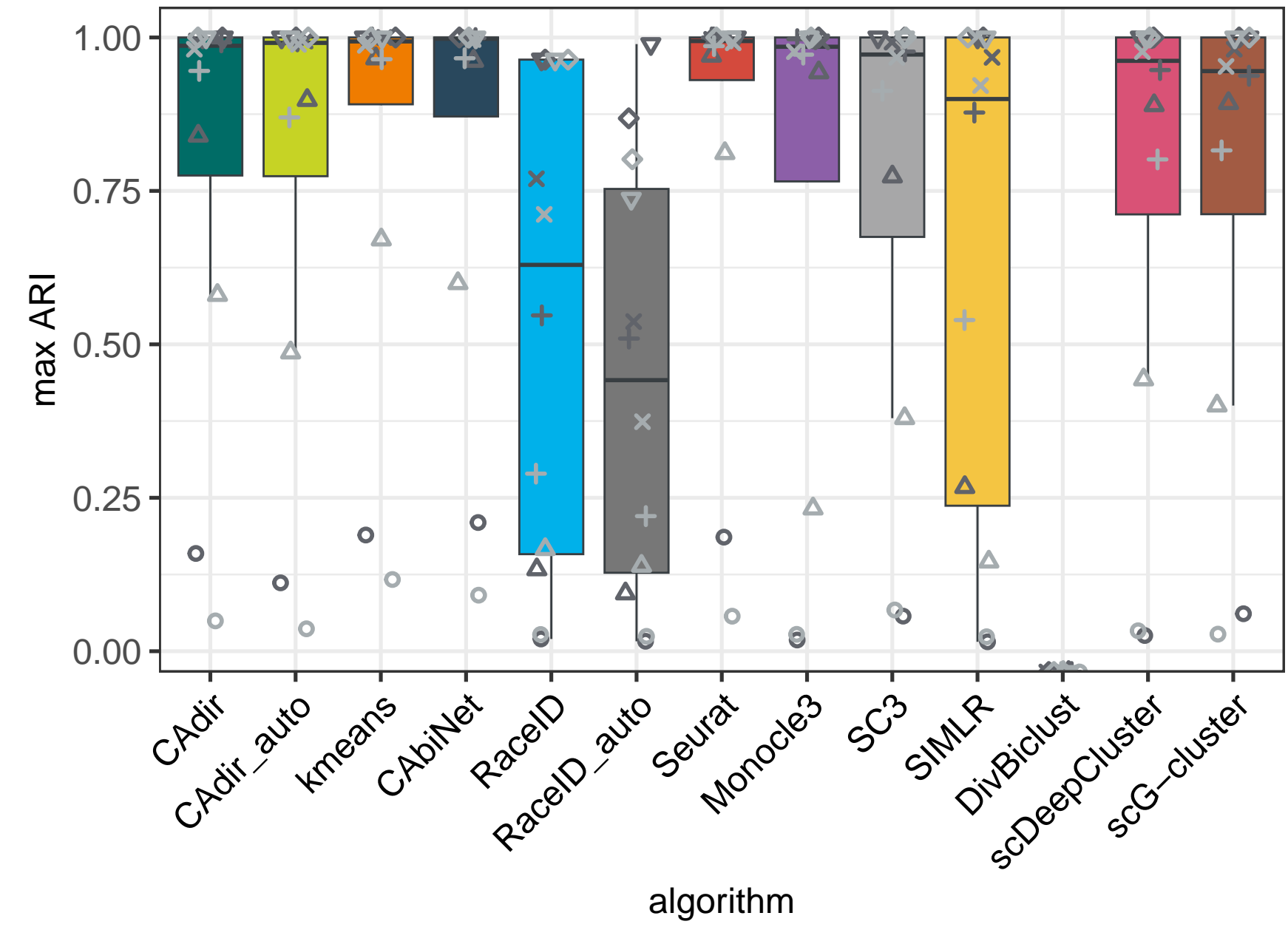**B**

Mean ARI on simulated data

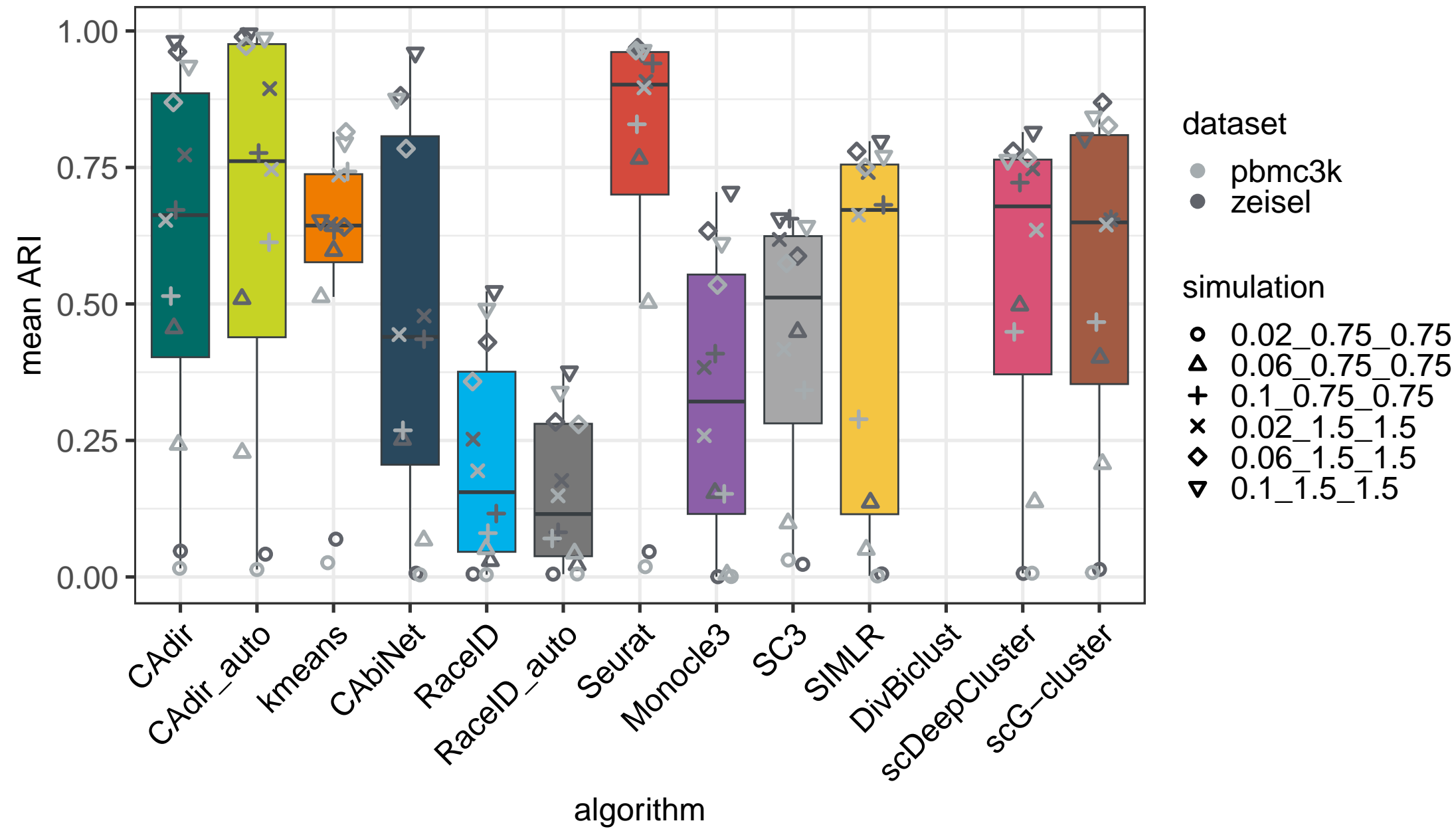**C**

Maximum CE

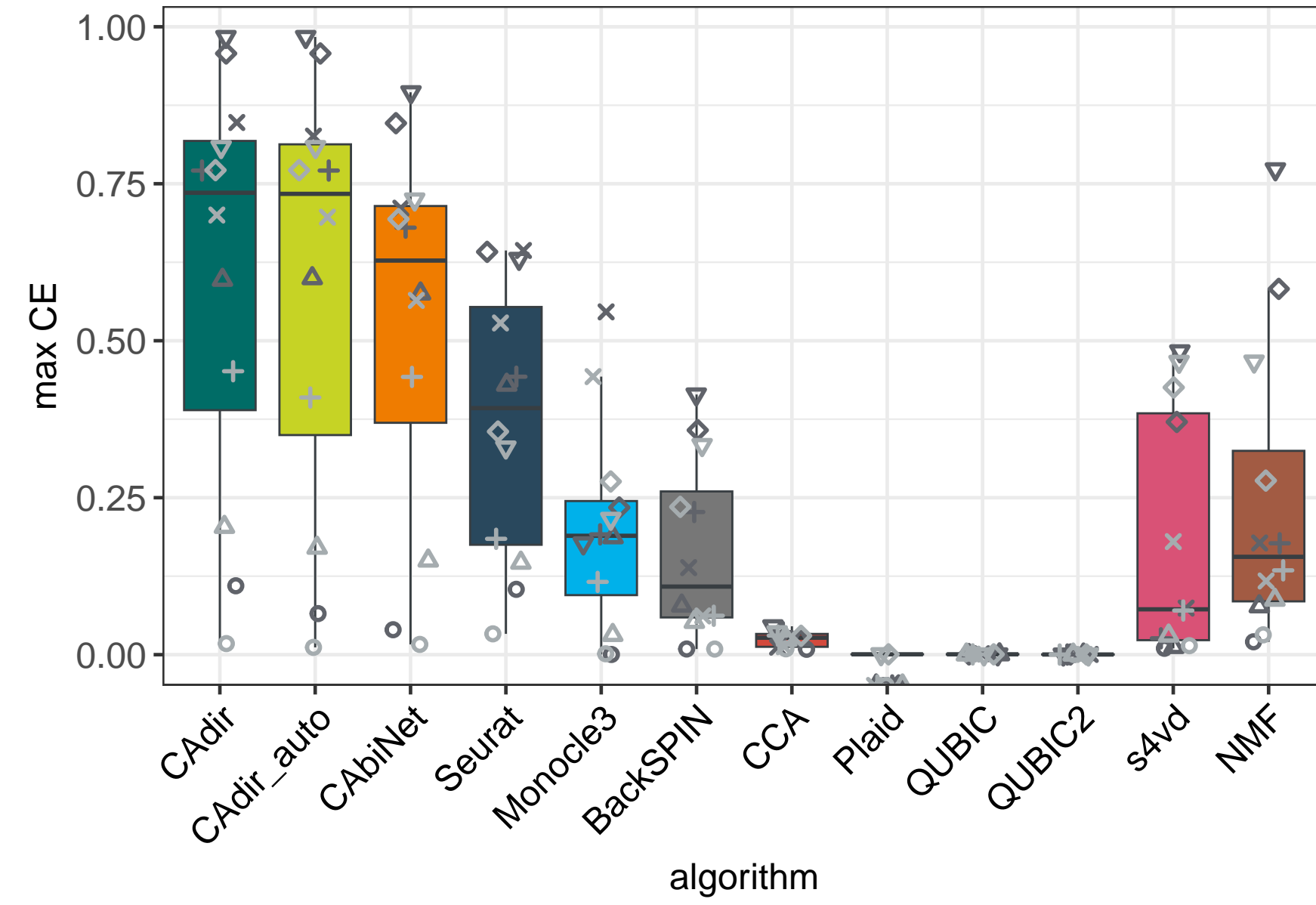**D**

Mean CE

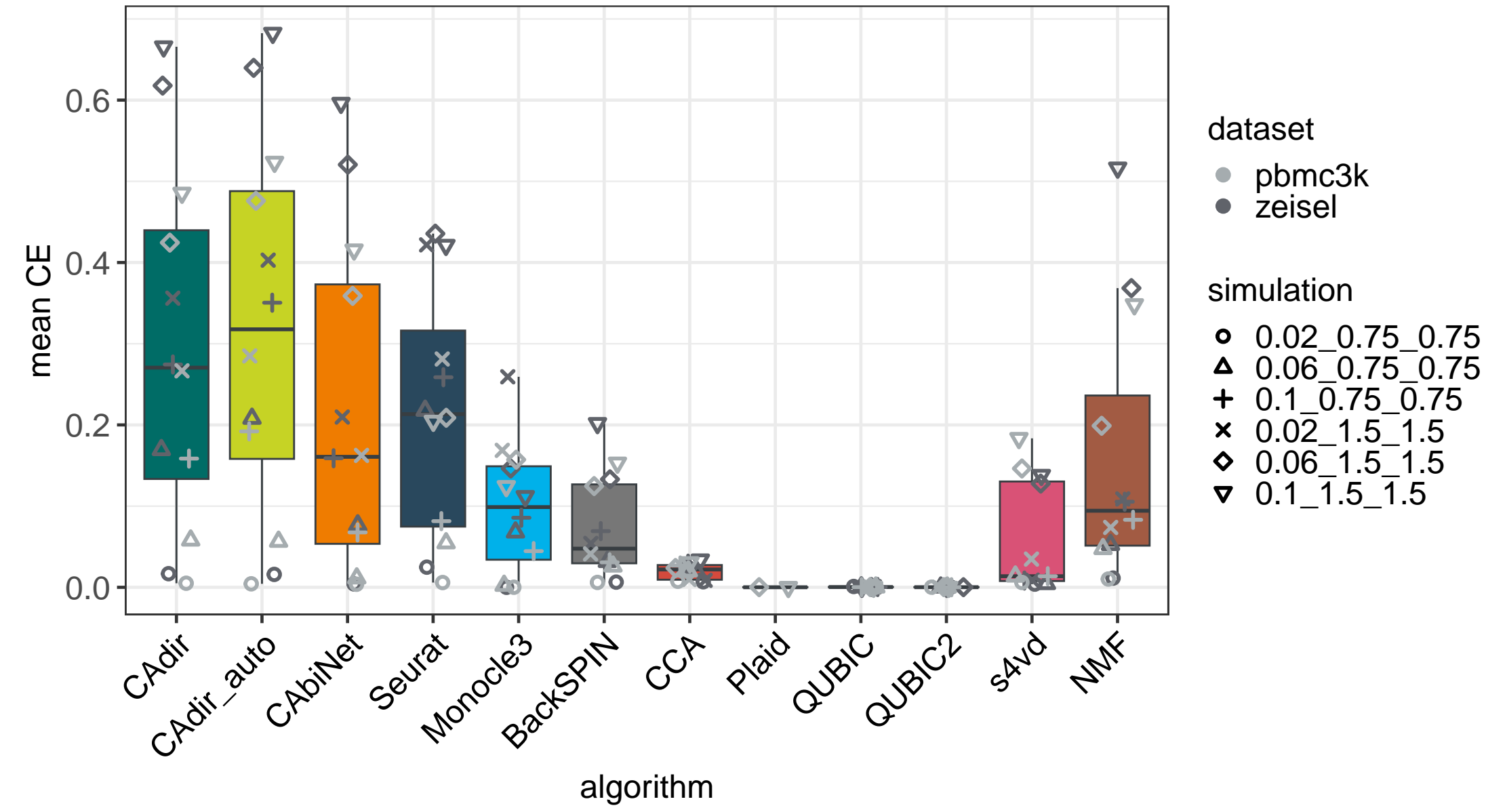

Supplement: S17 Fig — Biclustering benchmarking results for simulated data with increased gene dropout rate. The cell clustering is evaluated by A, the maximum ARI and B mean ARI of the cell clustering and the biclustering using the C, the maximum CE and D, the mean CE over all parameter combinations. Missing results for certain algorithms are because all (bi-)clustering runs for a data set either did not yield any interpretable results or all runs crashed (see, e.g., Plaid and DivBiclust). (PDF) [file pcbi.1014418.s018.pdf]

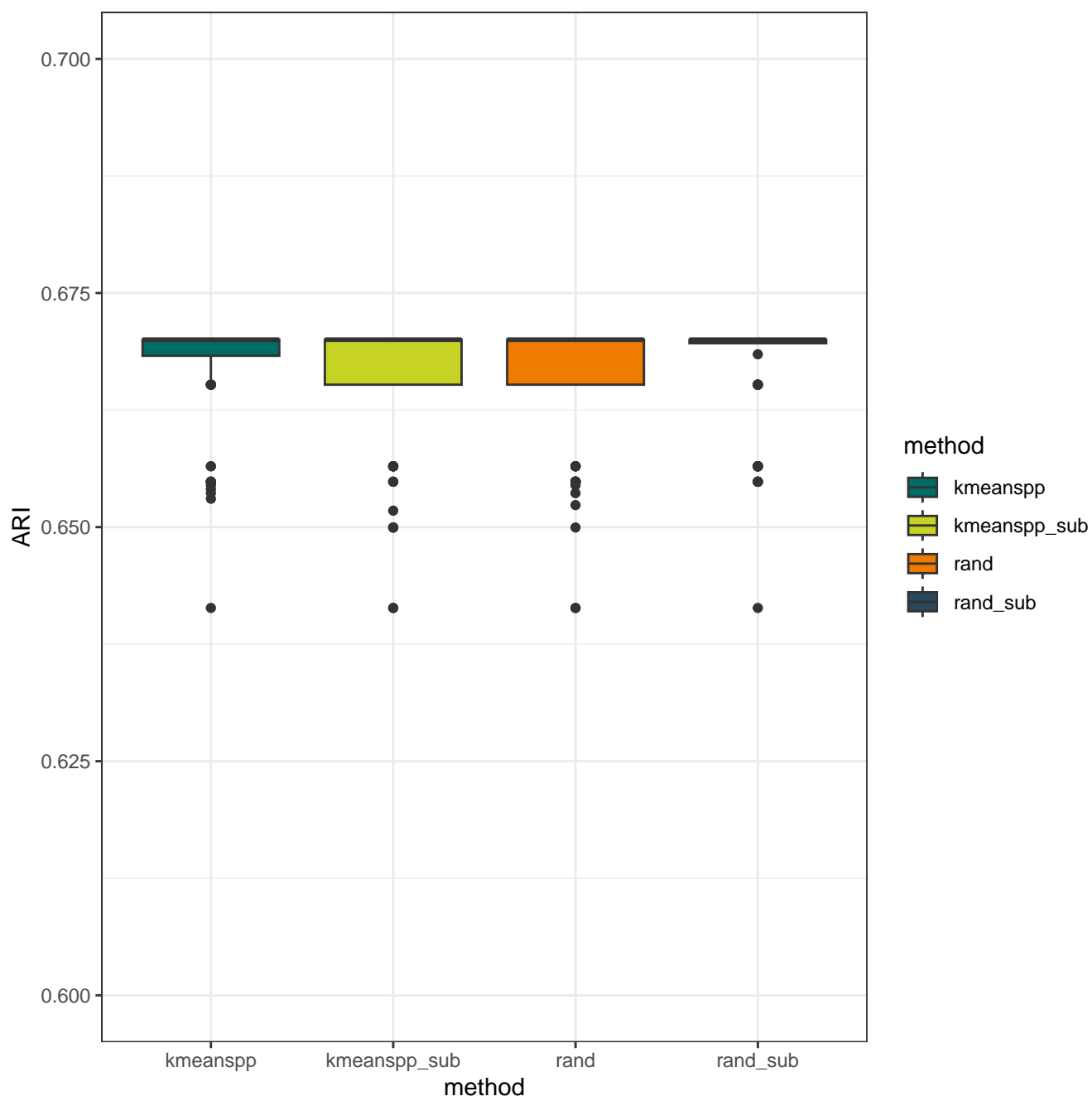

Supplement: S18 Fig — Adjusted Rand Index for clustering using kmeans++ (kmeansppp) and random initialization (rand) over 100 repetitions on the Tabula Muris Limb Muscle data for either k = 8 (ground truth) or k = 5 (kmeanspp_sub and rand_sub). (PDF) [file pcbi.1014418.s019.pdf]

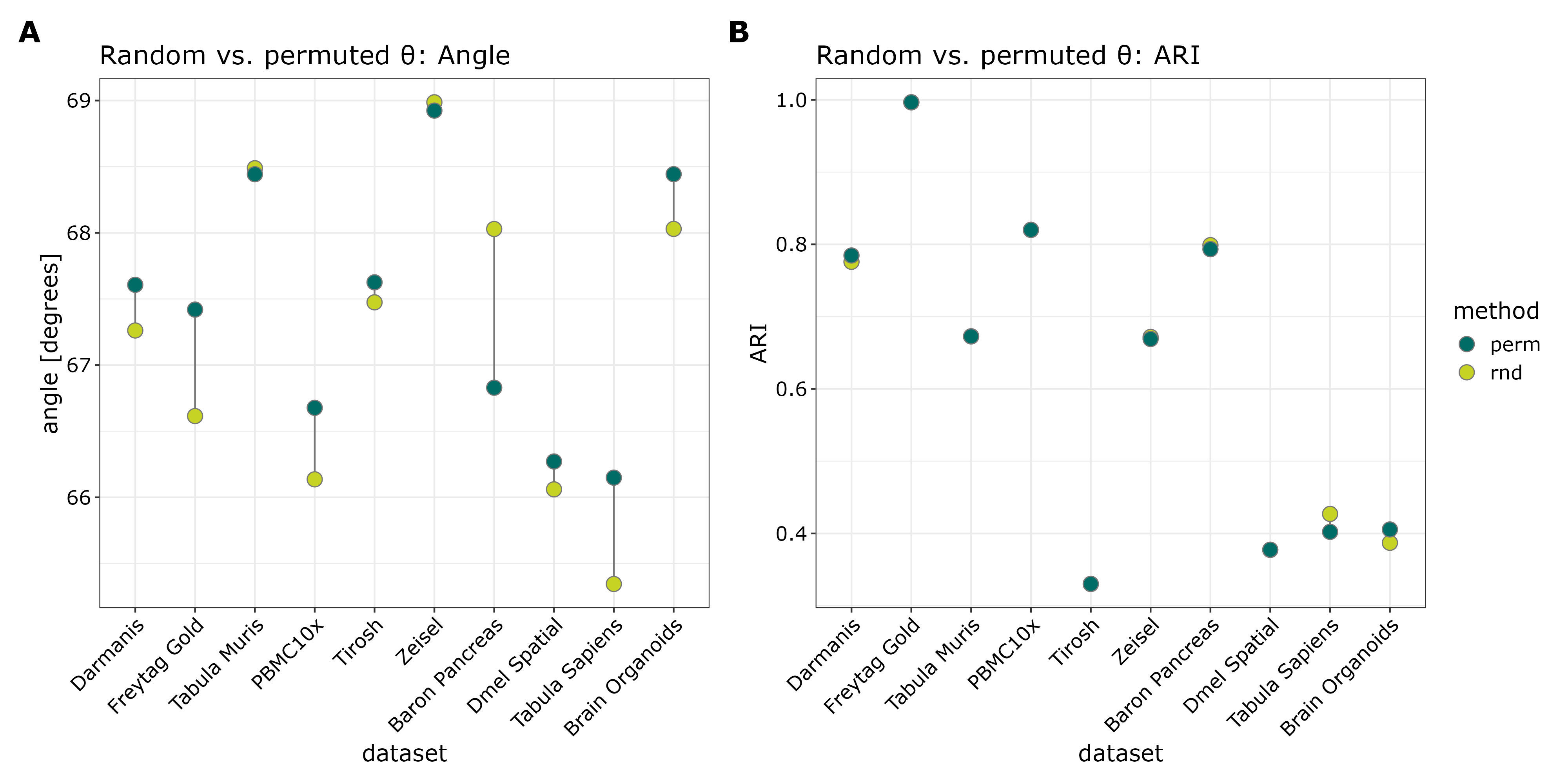

Supplement: S19 Fig — For each experimental data set used in the benchmarking, the cutoff angle θ is inferred either using the permutation based method (“perm”) or using randomized directions (“rand”). Panel A shows the inferred angle, whereas B shows the ARI of the clustering result using the inferred angle. (PNG) [file pcbi.1014418.s020.png]

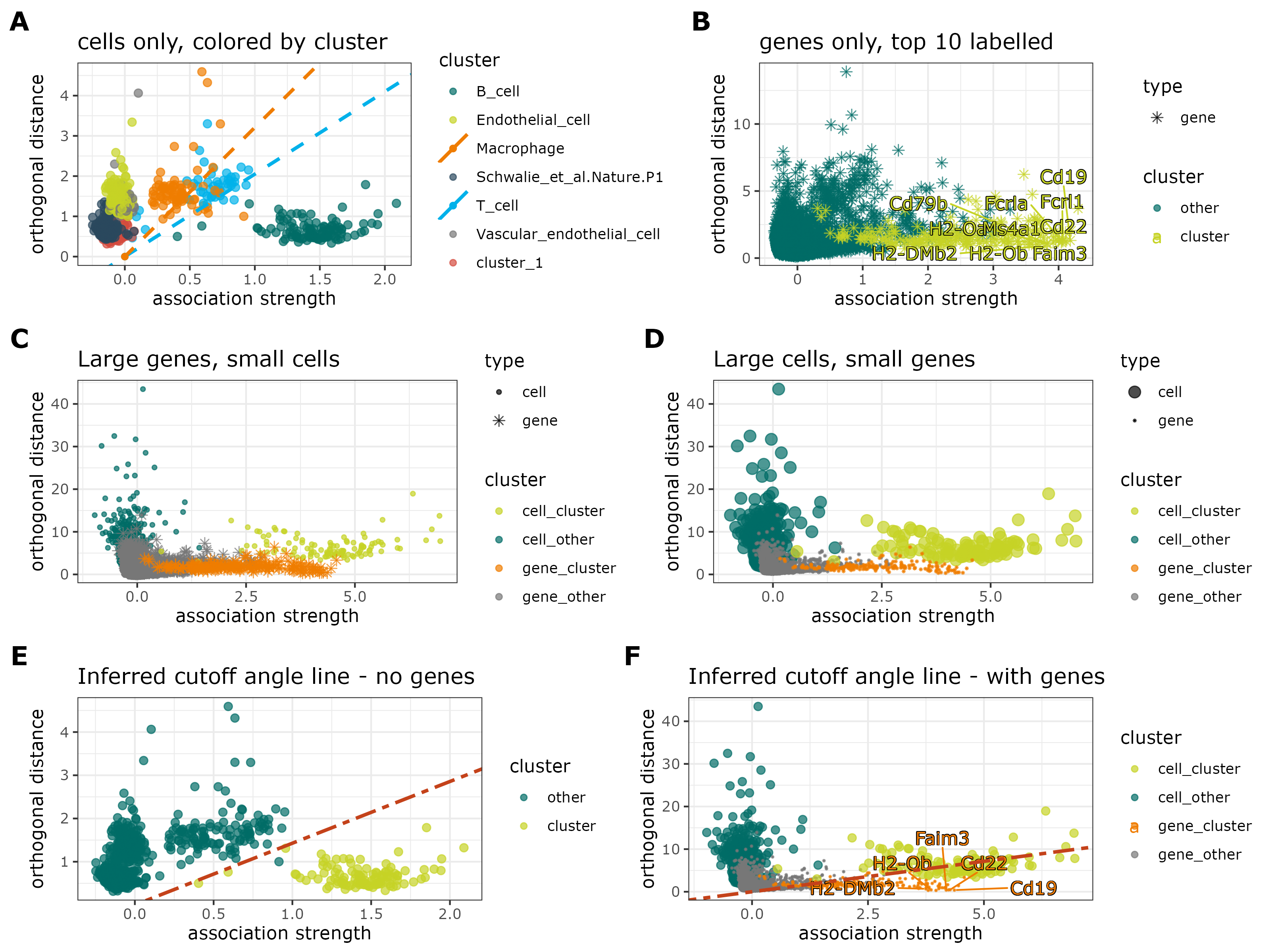

Supplement: S20 Fig — Association Plots for the B cell cluster of the Tabula Muris Limb Muscle data set. A, Cells are colored by their respective cluster and the T cell and Macrophage cluster directions are highlighted. B, Association Plot only showing the genes, with the top 10 most highly associated genes labelled. Depending on the main interest, the size of either C, the genes, or D, the cells can be increased to provide a better overview. Panel E shows the Association plot for the cells in principal coordinates. The red line has the same angle as the inferred cutoff angle θ. The clustered cells are clearly delineated by the cutoff line from other cell types. Similarly in F, cells and genes are plotted together. Genes that fall to the right and below the red cutoff angle line are the most cluster specific. (PNG) [file pcbi.1014418.s021.png]
