# Supplementary material for: CAdir: Joint clustering of cells and genes for single-cell transcriptomics with visualization-driven cluster quality assessment
Source: PLoS Comput Biol. 2026 Jun 30;22(6):e1014418. doi: 10.1371/journal.pcbi.1014418 (PMC13349309; doi:10.1371/journal.pcbi.1014418)
Supplement: S2 Fig — A, Association Plots of the corrected clustering of the PBMC3k data after removing outlier cells and genes. Clustered cells are colored in lime green and co-clustered genes in orange. Five genes with the highest Sθ-score are labelled. Other cells and genes are colored in dark green and grey respectively. B, Sankey plot comparing the corrected clustering (left) against the annotation obtained through the Seurat vignette (right). (PDF) [file pcbi.1014418.s003.pdf]

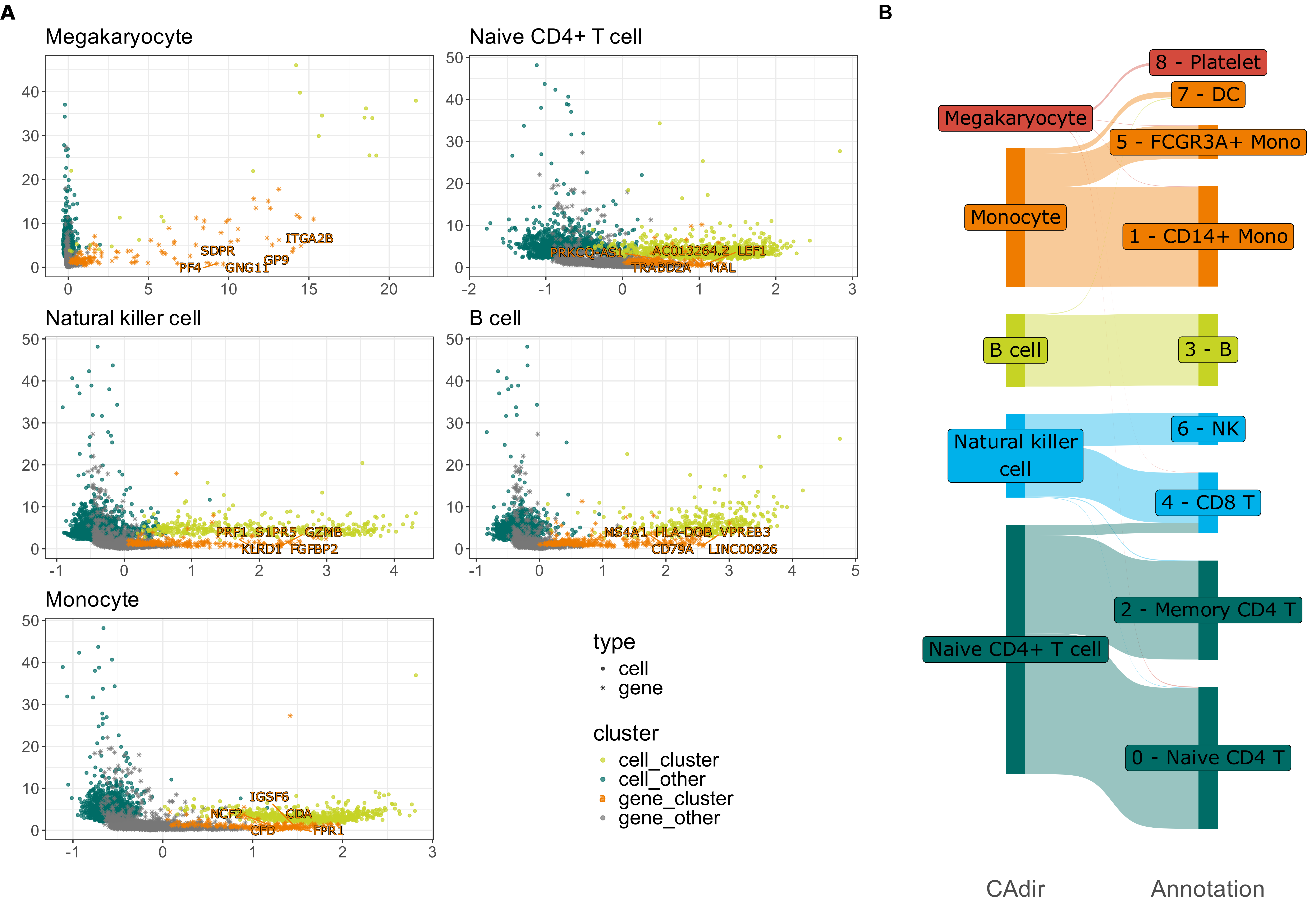
A
Megakaryocyte
SDPR
ITGA2B
GP9
PF4
GNG11
Naive CD4+ T cell
PRKCQ-AS1
AC013264.2
LEF1
TRABD2A
MAL
Natural killer cell
PRF1
S1PR5
GZMB
KLRD1
FGFBP2
B cell
MS4A1
HLA-DOB
VPREB3
CD79A
LINC00926
Monocyte
IGSF6
NCF2
CDA
CFD
FPR1
type
cell
gene
cluster
cell_cluster
cell_other
gene_cluster
gene_other
B
8 - Platelet
7 - DC
Megakaryocyte
5 - FCGR3A+ Mono
Monocyte
1 - CD14+ Mono
B cell
3 - B
6 - NK
Natural killer cell
4 - CD8 T
2 - Memory CD4 T
Naive CD4+ T cell
0 - Naive CD4 T
CAdir
Annotation
