# Supplementary material for: CAdir: Joint clustering of cells and genes for single-cell transcriptomics with visualization-driven cluster quality assessment
Source: PLoS Comput Biol. 2026 Jun 30;22(6):e1014418. doi: 10.1371/journal.pcbi.1014418 (PMC13349309; doi:10.1371/journal.pcbi.1014418)
Supplement: S5 Fig — The overlap between the co-clustered genes found by CAdir and scran’s scoreMarkers is determined by taking the same number of top ranked genes for both methods. A, Overlap between CAdir and scran using all co-clustered genes and B, using only the co-clustered genes with Sθ>0. (PDF) [file pcbi.1014418.s006.pdf]

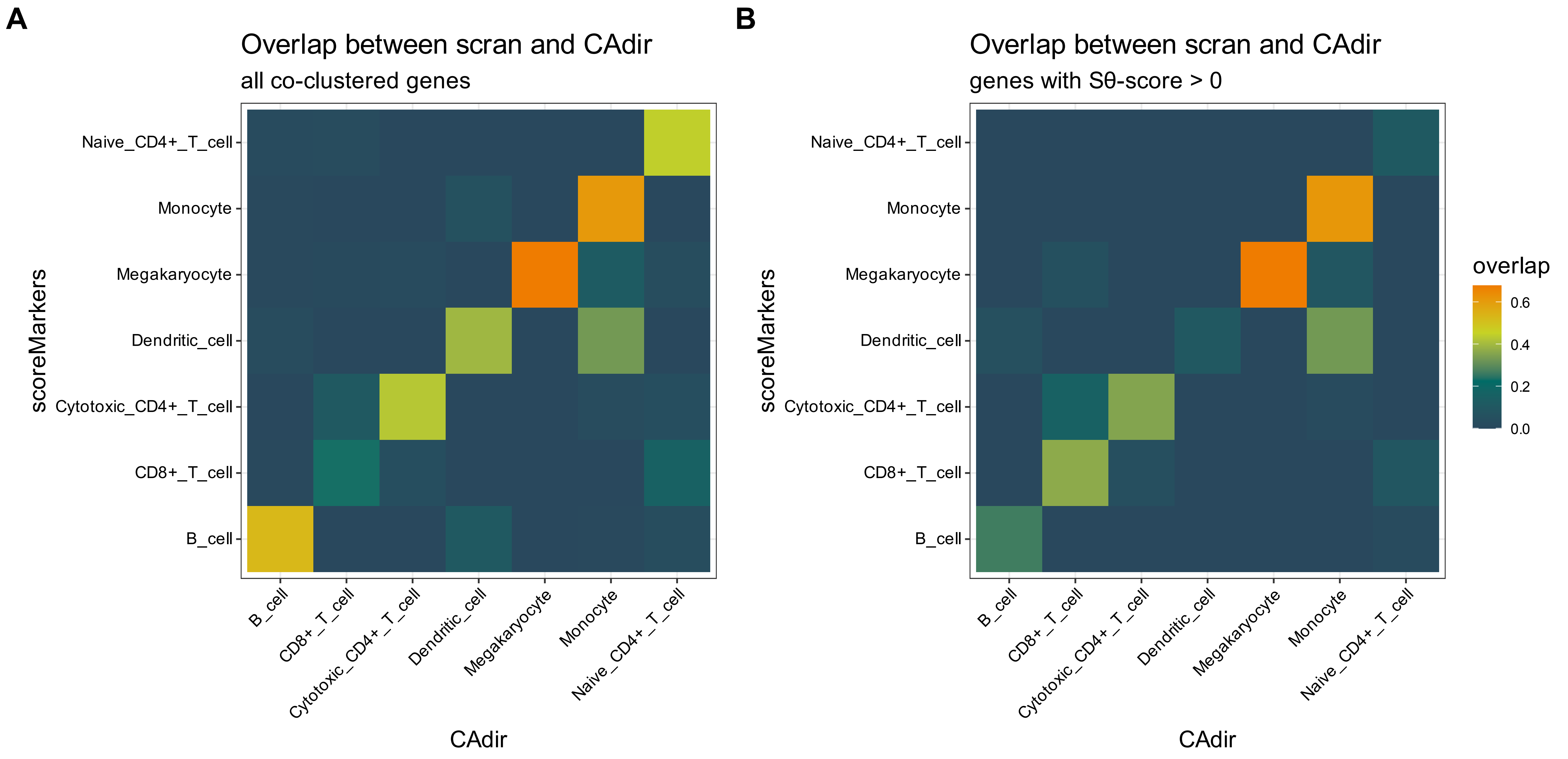
A
Overlap between scran and CAdir
all co-clustered genes
B
Overlap between scran and CAdir
genes with Sθ-score > 0
scoreMarkers
Naive_CD4+_T_cell
Monocyte
Megakaryocyte
Dendritic_cell
Cytotoxic_CD4+_T_cell
CD8+_T_cell
B_cell
CAdir
B_cell
CD8+_T_cell
Cytotoxic_CD4+_T_cell
Dendritic_cell
Megakaryocyte
Monocyte
Naive_CD4+_T_cell
overlap
0.6
0.4
0.2
0.0
