# Supplementary material for: CAdir: Joint clustering of cells and genes for single-cell transcriptomics with visualization-driven cluster quality assessment
Source: PLoS Comput Biol. 2026 Jun 30;22(6):e1014418. doi: 10.1371/journal.pcbi.1014418 (PMC13349309; doi:10.1371/journal.pcbi.1014418)
Supplement: S7 Fig — A, Computational runtime on simulated data of increasing size. Both x-axis and y-axis are in log10-scale for better interpretability. SC3 is marked in a dashed line as it is the only benchmarked cluster that does not cluster all cells in a conventional manner but instead only clusters 5000 cells and then attempts to assign the remaining cells to these clusters. B, Deviation of the number of retrieved clusters from the number of randomly sampled cell type clusters from the Tabula Muris cell atlas for different numbers of k used to initialize CAdir and different quantile cutoffs for the automatic angle determination (apl_q). Error bars indicate the standard deviation over 10 replicate runs. (PDF) [file pcbi.1014418.s008.pdf]

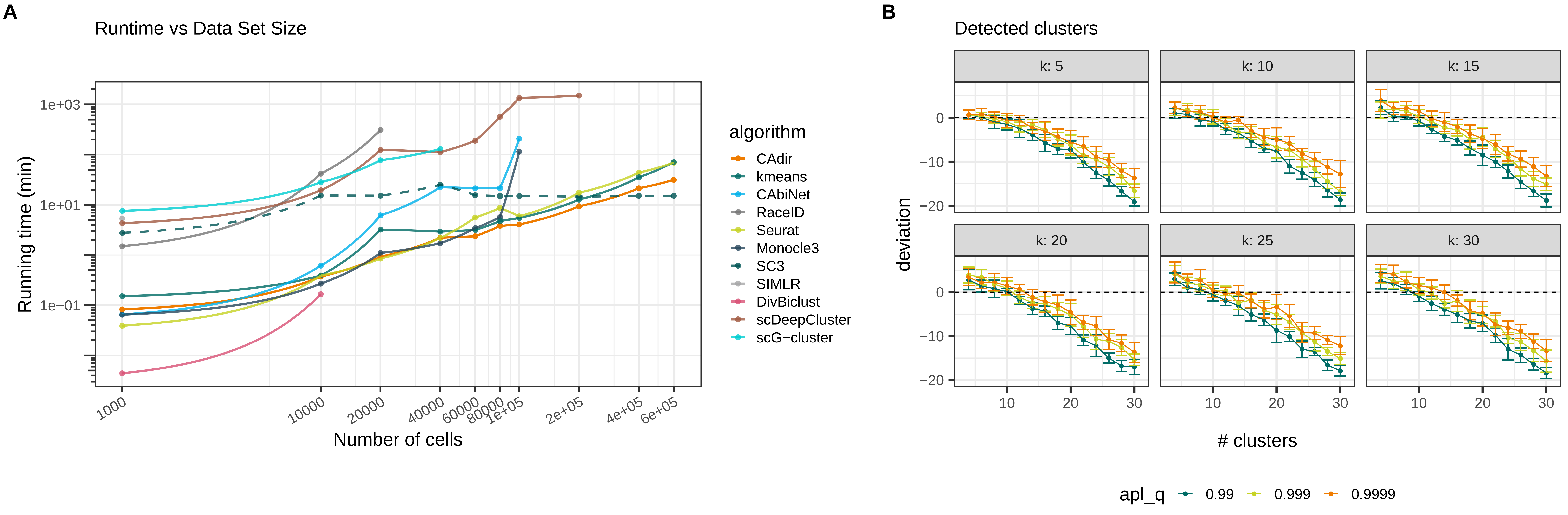
A
Runtime vs Data Set Size
Running time (min)
1e+03
1e+01
1e−01
1000
10000
20000
40000
60000
80000
1e+05
2e+05
4e+05
6e+05
Number of cells
algorithm
CAdir
kmeans
CAbiNet
RaceID
Seurat
Monocle3
SC3
SIMLR
DivBiclust
scDeepCluster
scG−cluster
B
Detected clusters
k: 5
k: 10
k: 15
k: 20
k: 25
k: 30
deviation
0
−10
−20
10
20
30
clusters
apl_q
0.99
0.999
0.9999
