# Supplementary material for: CAdir: Joint clustering of cells and genes for single-cell transcriptomics with visualization-driven cluster quality assessment
Source: PLoS Comput Biol. 2026 Jun 30;22(6):e1014418. doi: 10.1371/journal.pcbi.1014418 (PMC13349309; doi:10.1371/journal.pcbi.1014418)
Supplement: S11 Fig — Achieved ARI for A, the chosen number of CA dimensions and B, the Association Plot (APL) cutoff quantiles for experimental data sets. (PDF) [file pcbi.1014418.s012.pdf]

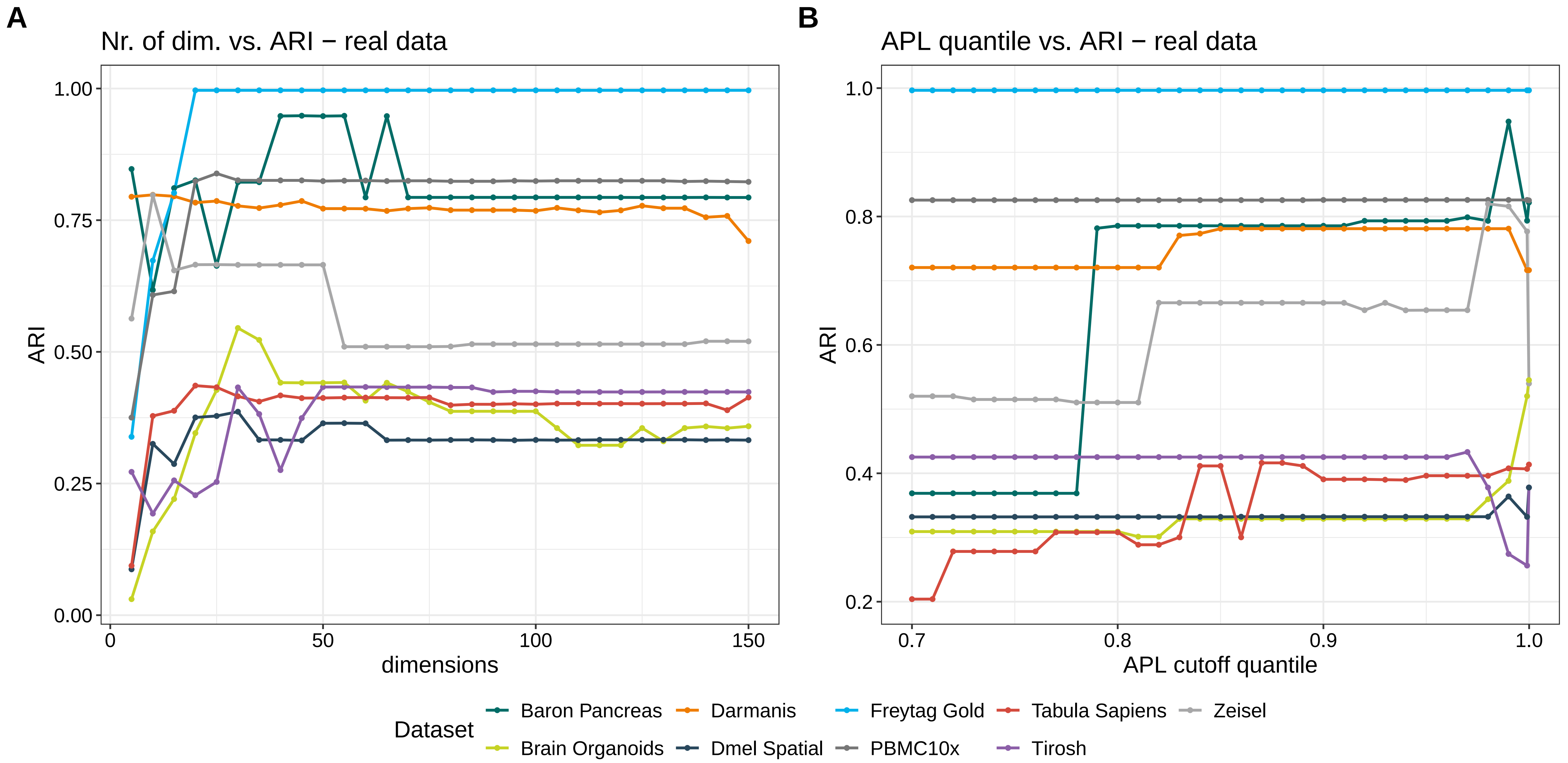
A
Nr. of dim. vs. ARI − real data
ARI
1.00
0.75
0.50
0.25
0.00
0
50
100
150
dimensions
B
APL quantile vs. ARI − real data
ARI
1.0
0.8
0.6
0.4
0.2
0.7
0.8
0.9
1.0
APL cutoff quantile
Dataset
Baron Pancreas
Darmanis
Freytag Gold
Tabula Sapiens
Zeisel
Brain Organoids
Dmel Spatial
PBMC10x
Tirosh
