# Supplementary material for: CAdir: Joint clustering of cells and genes for single-cell transcriptomics with visualization-driven cluster quality assessment
Source: PLoS Comput Biol. 2026 Jun 30;22(6):e1014418. doi: 10.1371/journal.pcbi.1014418 (PMC13349309; doi:10.1371/journal.pcbi.1014418)
Supplement: S12 Fig — A, Number of random directions in comparison to the inferred cutoff angle. B, Number of random directions compared to the achieved ARI. Overall, after approximately 100 iterations the inferred angle is stable and therefore a higher number of random directions does not change the resulting ARI meaningfully. (PDF) [file pcbi.1014418.s013.pdf]

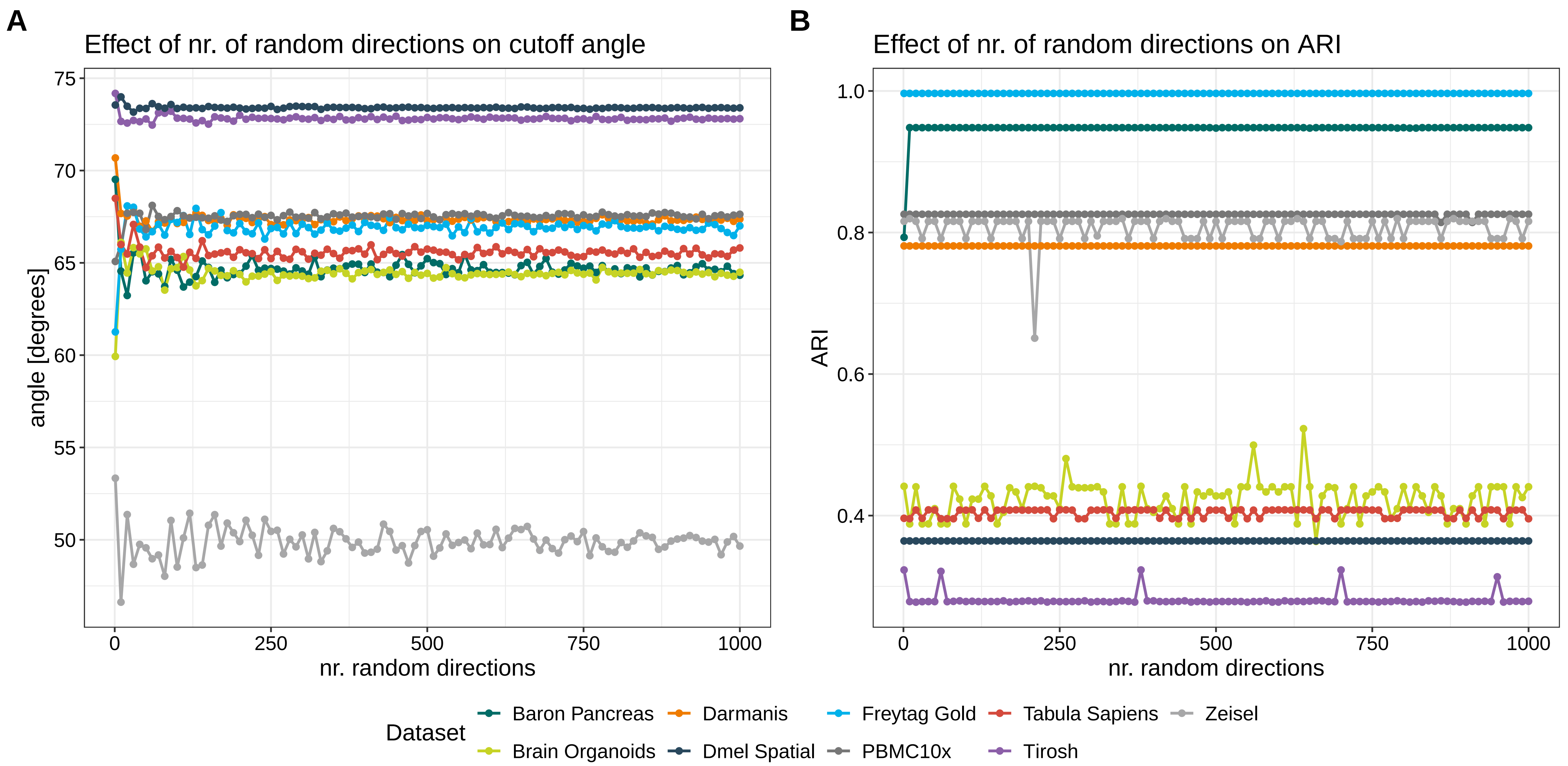
A
Effect of nr. of random directions on cutoff angle
angle [degrees]
75
70
65
60
55
50
0
250
500
750
1000
nr. random directions
B
Effect of nr. of random directions on ARI
ARI
1.0
0.8
0.6
0.4
0
250
500
750
1000
nr. random directions
Dataset
Baron Pancreas
Brain Organoids
Darmanis
Dmel Spatial
Freytag Gold
PBMC10x
Tabula Sapiens
Tirosh
Zeisel
