# Supplementary material for: CAdir: Joint clustering of cells and genes for single-cell transcriptomics with visualization-driven cluster quality assessment
Source: PLoS Comput Biol. 2026 Jun 30;22(6):e1014418. doi: 10.1371/journal.pcbi.1014418 (PMC13349309; doi:10.1371/journal.pcbi.1014418)
Supplement: S13 Fig — Obtained cell clustering ARI on A, experimental data and B, simulated data for 1–50 CAdir iterations. The same comparison was also performed for C, experimental and D, simulated data for the Dirclust sub-step. (PDF) [file pcbi.1014418.s014.pdf]

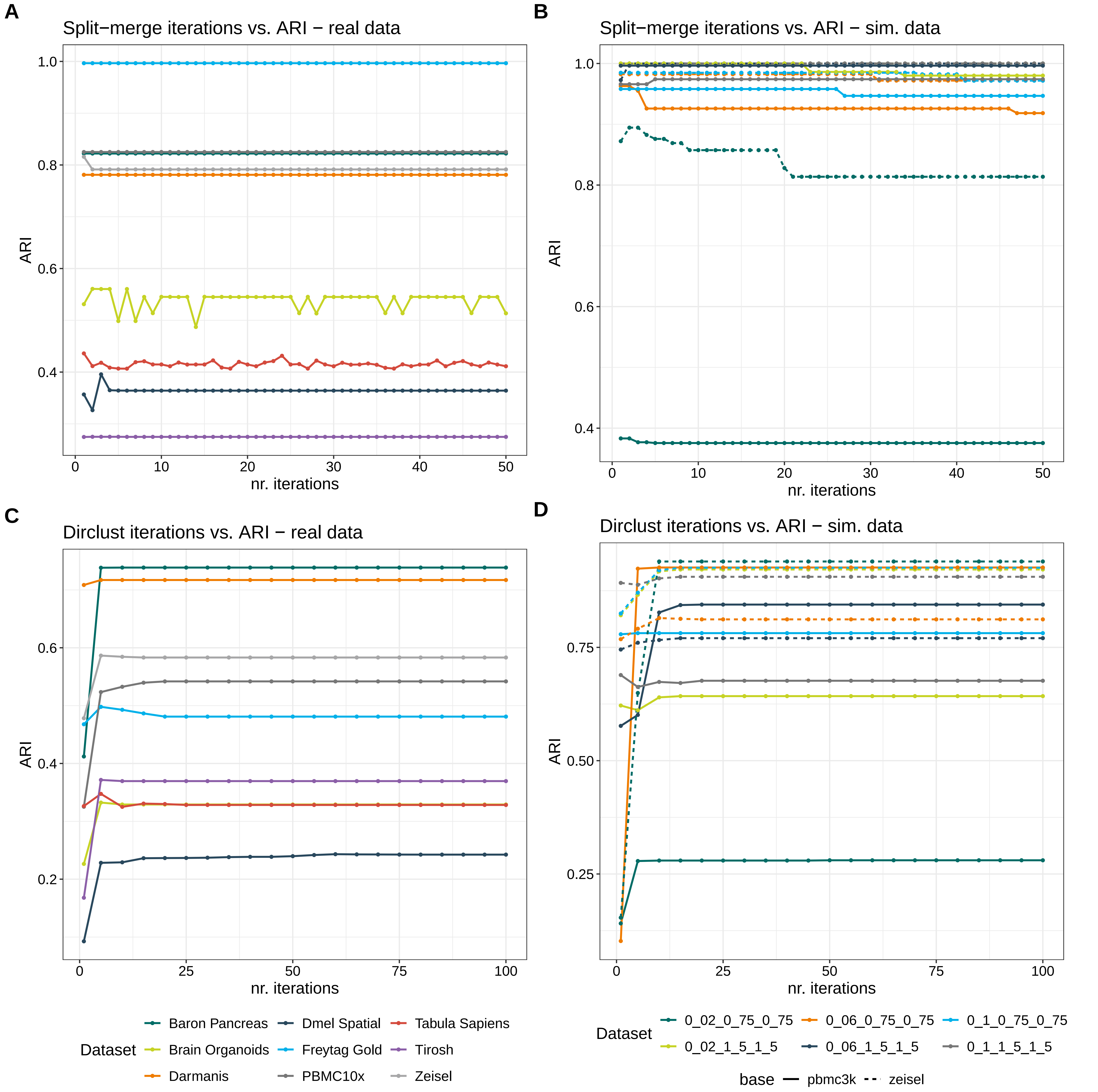

A
Split−merge iterations vs. ARI − real data
B
Split−merge iterations vs. ARI − sim. data
C
Dirclust iterations vs. ARI − real data
D
Dirclust iterations vs. ARI − sim. data
ARI
nr. iterations
Dataset
Baron Pancreas
Dmel Spatial
Tabula Sapiens
Brain Organoids
Freytag Gold
Tirosh
Darmanis
PBMC10x
Zeisel
0_02_0_75_0_75
0_06_0_75_0_75
0_1_0_75_0_75
0_02_1_5_1_5
0_06_1_5_1_5
0_1_1_5_1_5
base
pbmc3k
zeisel
